# Self-Healable, Injectable Hydrogel with Enhanced Clotrimazole Solubilization as a Potential Therapeutic Platform for Gynecology

**DOI:** 10.1021/acs.biomac.2c00691

**Published:** 2022-09-08

**Authors:** Monika Gosecka, Daria Jaworska-Krych, Mateusz Gosecki, Ewelina Wielgus, Monika Marcinkowska, Anna Janaszewska, Barbara Klajnert-Maculewicz

**Affiliations:** † Centre of Molecular and Macromolecular Studies, Polish Academy of Sciences, Sienkiewicza 112, 90-363 Lodz, Poland; ‡ Department of General Biophysics, Faculty of Biology and Environmental Protection, University of Lodz, 141/143 Pomorska Street, 90-236 Lodz, Poland

## Abstract

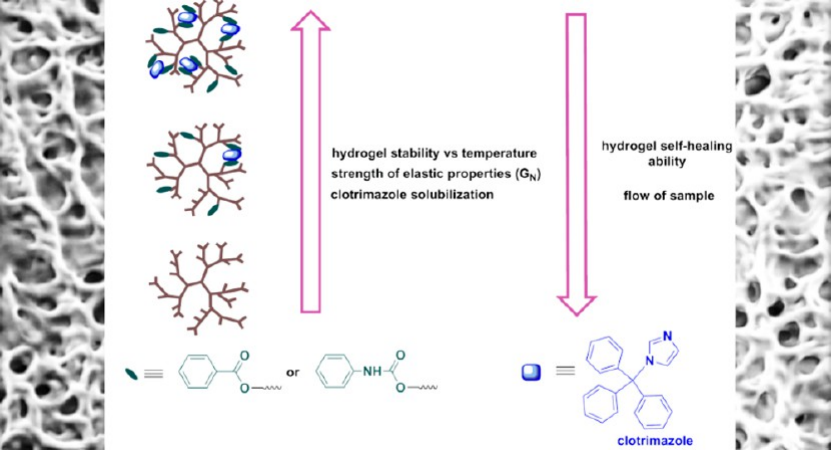

Injectable, self-healing hydrogels with enhanced solubilization of hydrophobic drugs are urgently needed for antimicrobial intravaginal therapies. Here, we report the first hydrogel systems constructed of dynamic boronic esters cross-linking unimolecular micelles, which are a reservoir of antifungal hydrophobic drug molecules. The selective hydrophobization of hyperbranched polyglycidol with phenyl units in the core via ester or urethane bonds enabled the solubilization of clotrimazole, a water-insoluble drug of broad antifungal properties. The encapsulation efficiency of clotrimazole increases with the degree of the HbPGL core modification; however, the encapsulation is more favorable in the case of urethane derivatives. In addition, the rate of clotrimazole release was lower from HbPGL hydrophobized via urethane bonds than with ester linkages. In this work, we also revealed that the hydrophobization degree of HbPGL significantly influences the rheological properties of its hydrogels with poly(acrylamide-*ran*-2-acrylamidephenylboronic acid). The elastic strength of networks (*G*_N_) and the thermal stability of hydrogels increased along with the degree of HbPGL core hydrophobization. The degradation of the hydrogel constructed of the neat HbPGL was observed at approx. 40 °C, whereas the hydrogels constructed on HbPGL, where the monohydroxyl units were modified above 30 mol %, were stable above 50 °C. Moreover, the flow and self-healing ability of hydrogels were gradually decreased due to the reduced dynamics of macromolecules in the network as an effect of increased hydrophobicity. The changes in the rheological properties of hydrogels resulted from the engagement of phenyl units into the intermolecular hydrophobic interactions, which besides boronic esters constituted additional cross-links. This study demonstrates that the HbPGL core hydrophobized with phenyl units at 30 mol % degrees via urethane linkages is optimal in respect of the drug encapsulation efficiency and rheological properties including both self-healable and injectable behavior. This work is important because of a proper selection of a building component for the construction of a therapeutic hydrogel platform dedicated to the intravaginal delivery of hydrophobic drugs.

## Introduction

Vulvovaginal candidiasis is one of the most frequent gynecological infections.^1^ Currently, the standard treatment of gynecological infections is the intravaginal administration of suppositories, which is very often an inefficient process, since the content of the suppository is commonly released uncontrolled and the amount of absorbed drug is limited. The lack of control over the drug delivery process may ultimately cause the disease recurrence in even more serious forms, e.g., due to drug resistance development, which consequently leads to chronic inflammation, miscarriage, and infertility. The current challenge in intravaginal therapies lies in the improvement of the drug formulations, which should extend the retention time of the carrier with the afflicted area in the vagina, assuring controlled delivery of drug molecules and increasing the drug bioavailability.

In the last decade, hydrogels, i.e., soft and porous cross-linked materials, became promising candidates for drug carriers dedicated to intravaginal therapies as an alternative to suppositories.^1^ In the design of the hydrogels for gynecological applications, apart from the biocompatibility, hydrophilicity, the rheological properties facilitating its administration, and retention of the drug carrier, the possibility of the network degradation after the fulfillment of its function should be taken into account. Therefore, dynamic hydrogels, i.e., networks constructed of reversible cross-links, are the most suitable systems for the construction of drug carriers. The reversible character of cross-links can assure not only the (self)-healing ability, injectability, and controlled diffusivity but also the gradual decomposition, which are of great importance in view of the intravaginal applications.^2−4^

The significant challenge in the design of hydrogel-based drug carriers in intravaginal therapies is, however, the discrepancy between the hydrogel hydrophilicity and hydrophobic character of numerous medicines routinely applied in gynecology. It is mainly overcome by the utilization of micelles, which core solubilizes hydrophobic drugs, whereas the hydrophilic shell keeps the molecules soluble in the aqueous medium.^5−8^ In addition, this strategy can also reduce drug side effects and protect drug molecules against possible degradation.^9^ The main disadvantage of using standard micelles in view of the network construction, however, is their instability at a concentration below cmc of the amphiphilic copolymers or under the shear force,^10^ which leads to the degradation of micelles. Unimolecular micelles, i.e., dendrimer-type,^11−13^ star-shaped,^14^ or hyperbranched^15,16^ amphiphilic macromolecules, in which all chains are covalently bound, overcome the limitations of standard micelles. Among hyperbranched polymers, hyperbranched polyglycidol, HbPGL, thanks to its biocompatibility,^15−21^ low viscosity,^22^ high hydrophilicity,^15^ and numerous functional groups,^22^ according to us, deserves a particular interest in view of the formation of hydrogel-based carriers for gynecology applications. Diol groups present in the terminal units (approx. 30 mol % of all constitutional units) in the macromolecule corona can form reversible cross-links with boronic acid moieties, generating both self-healable and injectable dynamic hydrogel systems.^2,16,29^ A tree-like structure of HbPGL delivers nanosized pockets for low-molecular drug molecules.^16,23,24^ The efficient encapsulation of water-insoluble drug solubilization in HbPGL, however, requires the prior hydrophobization of monohydroxyl linear units (approx. 40 mol % of all constitutional units)^25^ located in the HbPGL core.^26−29^ This synthetic strategy leads to the formation of unimolecular micelles, with the inner part being a reservoir for hydrophobic molecules, and a diol-rich shell that provides the solubility of the whole construct in water. The content of hydrophobic units incorporated into the HbPGL core, however, has to be carefully adjusted to assure the solubility of macromolecules in water. Until now, the hydrophobization of the HbPGL core was only performed with biphenyl derivatives achieving the encapsulation of nimodipine and pyrene.^27,28^ The solubility of biphenyl-enriched HbPGL derivatives in water was, however, significantly limited. Macromolecules, which over 45 mol % of all monohydroxyl units, i.e., above 18 mol % of all repeating units in the macromolecule, were modified and were insoluble in water.^26^ To overcome this limitation and assure a uniform hydrophobic environment in the HbPGL core for effective drug encapsulation, we decided to modify the HbPGL core with a smaller aromatic system, incorporating phenyl moieties. Since most of the antimicrobial drugs applied in gynecological therapies are water-insoluble and contain an aromatic ring in the structure, we expected HbPGL with a phenyl-enriched core to be effective in encapsulating drugs according to the principle “like dissolves like”. In this study, we used clotrimazole, 1-((2-chlorophenyl)diphenylmethyl)-1*H*-imidazole, a highly hydrophobic drug that exhibits a broad spectrum of antifungal activity, to show the potential of the HbPGL with the phenyl-rich core for the encapsulation of water-insoluble drugs.

The unimolecular micelles based on the internally hydrophobized hyperbranched polyglycidol, however, have never been applied to the formation of dynamic hydrogels based on boronic ester cross-links. Thus, the influence of the hydrophobization of the HbPGL core on the rheological properties of such hydrogels is unknown. In the case of hydrogels built from linear water-soluble macromolecules with partially hydrophobized repeating units, the enhanced toughness in comparison to the hydrogels constructed of the unmodified polymer has been reported.^30,31^ The association of hydrophobic moieties in the form of micelles, apart from the intentionally applied cross-linking mechanism, played the role of additional temporary junction zones and thus influenced the network properties.^30^ Hydrogels prepared by the micellar cross-linking copolymerization of acrylamide and *N*,*N*′-methylenebisacrylamide cross-linker in the presence of hydrophobic comonomers such as *N*-butyl, *N*-hexyl, *N*-octyl, and *N*,*N*-dihydroxyacrylamide showed fine-tuned toughness by adjusting the fraction of hydrophobic units and the length of hydrophobic chains.^31^ Moreover, the hydrogel of high mechanical stability constructed entirely on hydrophobic associations was demonstrated by Mihajlovic et al.^32^ The multiblock copolymer of hydrophilic poly(ethylene glycol) and hydrophobic dimer fatty acid (DFA) was arranged into the three-dimensional (3D) network thanks to the self-assembling of DFA units in water, which played the role of micellar-like cross-links.

In this study, we focus on the influence of the degree of hydrophobization of the HbPGL core with phenyl groups incorporated via ester or urethane linkages on the rheological properties of hydrogels constructed with poly(acrylamide-*ran*-2-acrylamidephenylboronic acid), such as injectability, flow behavior, and self-healing properties. The optimization of a degree of HbPGL core hydrophobization is necessary to attain the hydrogel platform, displaying the ability to encapsulate clotrimazole and suitable rheological characteristics important for gynecological applications. To the best of our knowledge, it is the first report on the formation of hydrogels composed of the internally hydrophobized HbPGL dedicated as drug carriers for antimicrobial gynecological therapies. Our proposed hydrogel systems can overcome the limitations of currently accessible commercial drug formulations in gynecological therapies.

## Experimental Section

### Materials

Glycidol and 1,1,1-tris(hydroxymethyl)propane were purchased from Sigma-Aldrich. 2,2-Dimethoxypropane, benzoyl chloride, and phenyl isocyanate were purchased from Alfa Aesar. Anhydrous pyridine was purchased from Acros Organics. PTSA (Sigma-Aldrich) was dried with benzene. Glycidol was dried with 4 Å molecular sieves and distilled under reduced pressure. Comonomer 2-acrylamidephenylboronic acid pinacol ester, 2-AAPBAE, was synthesized according to the procedure reported in ref (33). The α,α′-azobis(isobutyronitrile), AIBN (Fluka), was recrystallized from methanol. Clotrimazole (Sigma-Aldrich) was used as received. Dialysis tubes (SnakeSkin TM 3.5 K MWCO) were purchased from Thermo Fisher Scientific. Surfactant-free cellulose acetate (SFCA, 0.8 μm) filters were purchased from Sartorius. Deionized water was prepared in SolPure XIO P (Elkar, Poland), where conductivity was equal to 0.055 μS. Na_2_HPO_4_ and KH_2_PO_4_ were purchased from Chempur. Tween80 was purchased from Karl Roth. Acetonitrile for HPLC-super gradient was purchased from POCH.

### Synthesis of Hyperbranched Polyglycidol (HbPGL)

The synthesis of hyperbranched polyglycidol was carried out in a thermostated glass reactor equipped with a steel mechanical stirrer under an argon atmosphere. Ten percent of hydroxyl groups of 1,1,1-tris(hydroxymethyl)propane (94 mg; 7 × 10^–4^ mol) were converted into alcoholates in tetrahydrofuran (THF) using NaH (2.1 × 10^–4^ mol). Twenty-five milliliters of glycidol was dropped into the reactor at a rate of 2 mL/h, and the polymerization was conducted for 24 h at 95 °C. The product was dissolved in methanol, twice precipitated into acetone, and dried. Then, the polymer was dissolved in deionized water and dialyzed using dialysis tubes.

**Scheme 1:**
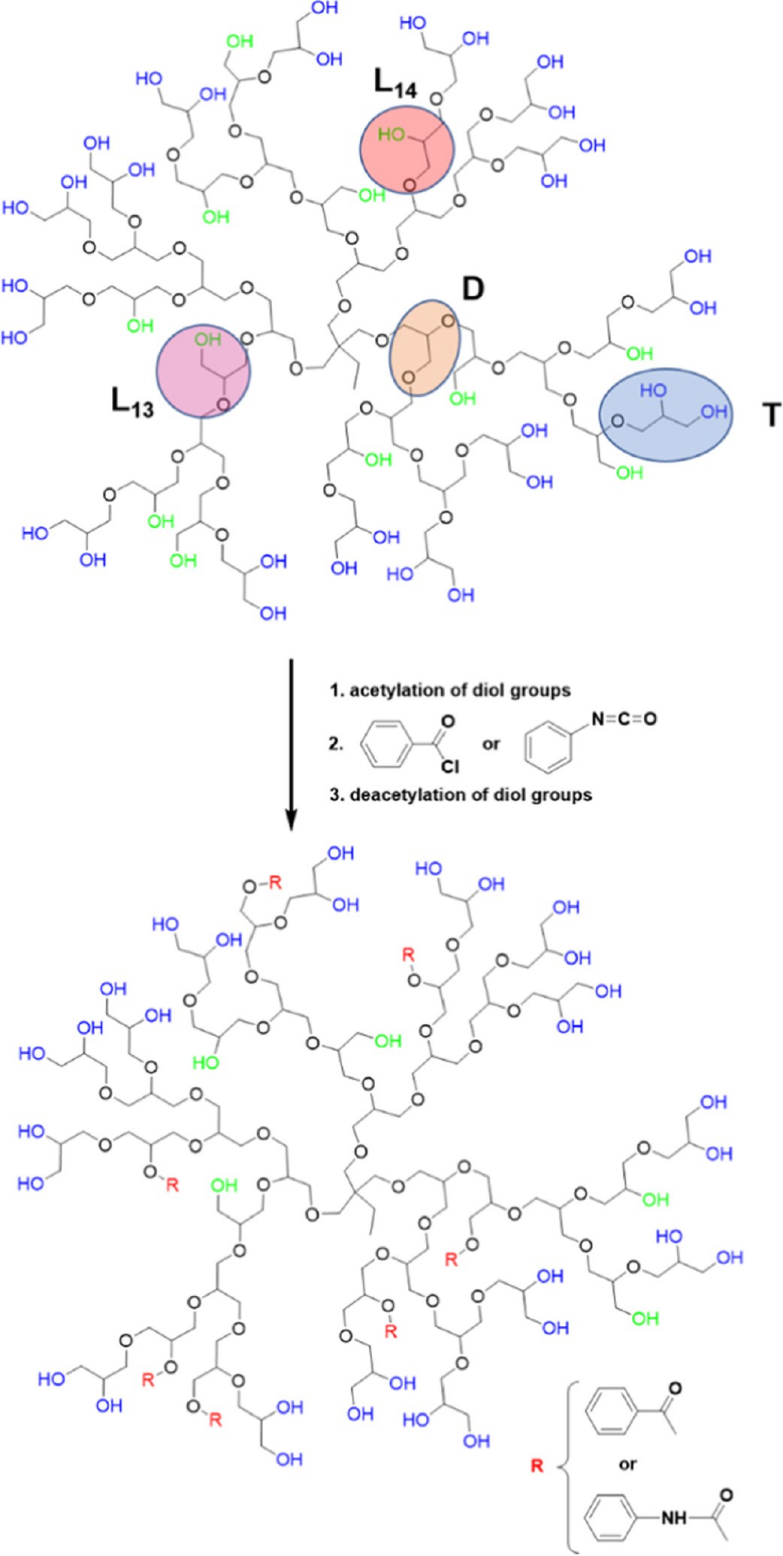
Synthetic Route Leading to the Internal Hydrophobization of HbPGL with Phenyl Units Incorporated *via* Ester or Urethane Bonds L_13_ and L_14_ denote the linear constitutional units of HbPGL, whereas D and T units correspond to dendritic and terminal units, respectively.

Degree of branching (DB) of synthesized neat HbPGL was 0.56. The molar fraction of dendritic (**D**) and linear constitutional units **L**_**13**_ and **L**_**14**_ bearing monohydroxyl groups was 0.27 and 0.40, respectively, whereas the molar fraction of terminal units (**T**) containing diol moieties was 0.33. **D**, **L**_**13**_, **L**_**14**_, and **T** units are denoted in Scheme 1. The weight average molecular weight and the molecular weight distribution were *M*_w_ = 7800 and *Đ* = 1.70, respectively.

### Synthesis of Hyperbranched Polyglycidol Acetal (AC-HbPGL)

The mixture of HbPGL (24 g, 0.127 mol of diol units), 2,2-dimethoxypropane (156 mL, 1.27 mol), and PTSA (0.322 g, 1.86 mmol) was ultrasonicated for 3 h at 40 °C. Then, the crude product was diluted with chloroform and extracted three times with saturated Na_2_CO_3_ solution to remove PTSA. The organic phase was dried over MgSO_4_ and dialyzed in chloroform for 24 h. The product was dried under a vacuum and analyzed with ^1^H and ^13^C NMR spectroscopies in DMSO-*d*_6_ to confirm that all diol groups are protected.

^1^H NMR (500 MHz, MeOD-*d*_4_) δ (ppm): 4.28 (CH–T-Acet); 4.08 (CH_2_–T-Acet); 3.89 (CH_2_–T-Acet); 3.85–3.40 (HbPGL backbone); 1.41 (CH_3_–T-Acet); 1.36 (CH_3_–T-Acet); 0.93 (CH_3_-initiator).

^13^C NMR (500 MHz, MeOD-d_4_) δ (ppm): 109.10–109.02 (C–T-Acet); 80.09 (CH–L_13_); 78.87–78.52 (CH–D); 74.95–74.75 (CH–T-Acet); 72.66 (CH_2_–2 L_14_); 72.13 (CH_2_–T-Acet); 71.12 (CH_2_–2D); 69.51 (CH_2_–L_13_); 69.24 (CH–L_14_); 66.31 (CH_2_–T-Acet); 61.43 (CH_2_–L_13_); 25.94–24.50 (CH_3_–T-Acet).

### Hydrophobization of AC-HbPGL Core with Benzoate Groups (Ester Derivative)

AC-HbPGL was dried with benzene by azeotropic distillation under argon before the modification. Then, the polymer was dissolved in pyridine in the argon atmosphere and the resulting solution was chilled to 0 °C. Benzoyl chloride was slowly added. After dropping the entire amount of benzoyl chloride, the solution was allowed to reach room temperature and left to stir for 24 h. The solvent was evaporated, and the product was dissolved in dimethyl sulfoxide (DMSO) and dialyzed for 24 h with at least three solvent exchanges. Then, DMSO was removed by evaporation under reduced pressure. The degree of modification was calculated based on the relation between the integration of acetal protons (in the range of the chemical shift between 1.15 and 1.35 ppm) and the integration of aromatic protons of benzoyl groups in the range from 7.30 to 8.10 ppm in ^1^H NMR spectra. Using different amounts of benzoyl chloride to hydrophobize the HbPGL core, the polymers of different degrees of modification were obtained. After the determination of the hydrophobization degree, the diol groups were deprotected by adding the aqueous solution of 0.1 M HCl to the polymer solution in DMSO and stirred overnight. Finally, the mixture was dialyzed against deionized water and analyzed with ^1^H, ^13^C INVGATE, DEPT, and ^1^H DOSY NMR spectroscopy. The characteristics of HbPGL hydrophobized *via* ester linkages are given in Table 1.

**Table 1:** Characteristics of Internally Hydrophobized HbPGL Macromolecules Synthesized in the Reaction with Benzoyl Chloride

polymer	molar fraction of hydrophobized constitutional units bearing monohydroxyl groups	molar fraction of hydrophobized constitutional units	number of hydrophobic moieties per one macromolecule	solubility in water at RT	*T*_g_ (°C) (DSC)
**HbPGL_BE4**	4.1	1.6	2	soluble	–24.7
**HbPGL_BE15**	14.8	5.9	6	soluble	–26.4
**HbPGL_BE20**	20.4	8.2	8	soluble	–22.3
**HbPGL_BE27**	27.5	11.0	12	soluble	–16.8
**HbPGL_BE37**	37.2	14.9	16	soluble	–18.7
**HbPGL_BE49**	49.4	19.8	21	soluble	–10.2
**HbPGL_BE58**	57.6	23.0	24	soluble	–3.6
**HbPGL_BE74**	74.1	29.6	31	limited solubility	1.7
**HbPGL_BE81**	81.1	32.4	34	limited solubility	0.4

The description of ^1^H and ^13^C NMR spectra of a final product, i.e., after diol group deprotection.

^1^H NMR (500 MHz, DMSO-*d*_6_) δ (ppm): 7.95–7.50 (aromatic protons); 5.25 (CH, L_14_ hydrophobized); 4.90–4.35 (OH groups); 4.23 (CH_2_, L_14_ hydrophobized); 4.00–3.00 (HbPGL backbone).

^13^C NMR (500 MHz, DMSO-*d*_6_) δ (ppm): 166.11, 165.77 (C=O, ester), 133.81 (4-C, Ar), 130.21–129.19 (1-C, 2-C, 3-C, 5-C, 6-C), 80.26 (CH–L_13_-hydrophobized), 78.61–78.31 (CH–D), 73.29 (CH_2_–2 L_14_), 72.10–71.23 (CH_2_–T, D), 70.94 (CH–T), 69.97 (CH_2_–L_13_-hydrophobized), 69.31 (CH_2_–L_13_), 69.02 (CH–L_14_-hydrophobized), 63.53 (CH_2_–T), 61.43 (CH_2_–L_13_).

### Hydrophobization of AC-HbPGL Core with Phenyl Carbamate Groups (Urethane Derivative)

AC-HbPGL was dried with benzene by its distillation under argon prior to the modification. Then, the polymer was dissolved in pyridine in the argon atmosphere and the resulting solution was stirred and heated to 50 °C. The phenyl isocyanate was slowly added to the solution, and the reaction was conducted for 24 h. Then, the mixture was dialyzed against DMSO. DMSO was removed by evaporation under reduced pressure. The degree of modification was estimated using ^1^H NMR spectroscopy based on the comparison of the relation of the integration of acetal protons (in the range of the chemical shift between 1.15 and 1.35 ppm) and aromatic protons in the range from 6.75 to 7.60 ppm. Using different amounts of phenyl isocyanate to modification of the HbPGL core, the polymers of different degrees of hydrophobization were obtained. After the determination of the hydrophobization degree, the diol groups were deprotected by adding an aqueous solution of 0.1 M HCl to the polymer solution in DMSO and stirring overnight. Finally, the mixture was dialyzed against deionized water and analyzed with ^1^H, ^13^C INVGATE, DEPT, and ^1^H DOSY NMR. The characteristics of hydrophobized HbPGL *via* urethane linkages are given in Table 2.

**Table 2:** Characteristics of Internally Hydrophobized HbPGL Macromolecules Synthesized in the Reaction with Phenyl Isocyanate

polymer	molar fraction of hydrophobized constitutional units bearing monohydroxyl groups	molar fraction of hydrophobized constitutional units	number of hydrophobic moieties per one macromolecule	solubility in water at RT	*T*_g_ (°C) (DSC)
**HbPGL_PC4**	3.9	1.6	2	soluble	–20.2
**HbPGL_PC16**	16.3	6.5	7	soluble	–16.6
**HbPGL_PC31**	31.3	12.5	13	soluble	–8.2
**HbPGL_PC55**	55.0	22.0	24	limited solubility	8.6
**HbPGL_PC82**	82.2	32.9	34	insoluble	25.0

The description of ^1^H and ^13^C NMR spectra of a final product, i.e., after diol group deprotection.

^1^H NMR (500 MHz, DMSO-*d*_6_) δ (ppm): 9.71 (NH); 7.45–6.97 (aromatic protons); 4.75 (CH, L_14_-hydrophobized); 4.80–4.35 (OH groups); 4.20 (CH_2_, L_14_-hydrophobized); 3.80–3.20 (HbPGL backbone).

^13^C NMR (500 MHz, MeOD-d_4_): 154.72, 154.34 (C=O, urethane), 140.36 (1-C, Ar), 130.01 (3-C, 5-C, Ar), 123.68 (4-C), 119.48 (2-C, 6-C, Ar), 81.18 (CH–L_1,3_-hydrophobized), 79.41–79.13 (CH–D), 74.12 (CH_2_–2 L_1,4_), 72.91–72.10 (CH_2_–T, 2D), 71.75 (CH–T), 70,98 (CH_2_–L_1,3_), 70,62 (CH_2_–L_1,3_-hydrophobized), 70.13 (CH–L_1,4_-hydrophobized), 69.84 (CH–L_1,4_), 64.34 (CH_2_–T), 62.15 (CH_2_–L_1,3_).

### Synthesis of Poly(AM-*ran*-2-AAPBA) and Poly(acrylamide-*ran*-2-acrylamidephenylboronic Acid) with 9.0 mol % of 2-AAPBA

An acrylamide copolymer with a 9.0 mol % 2-AAPBA content has been synthesized using a conventional radical copolymerization of acrylamide (2 g; 28.10 mmol) and 2-acrylamidephenylboronic acid pinacol ester (0.613 g; 2.24 mmol) initiated with AIBN. The initial molar ratio of comonomers to AIBN was 220:1. Polymerization was carried out in 15 mL of dimethylformamide (DMF)/dioxane mixture (5:1 v/v) at 70 °C for 16 h. The polymerization mixture was diluted in water, and the copolymer was precipitated into acetone and dried. Next, the copolymer was dissolved in an alkaline solution of NaOH (1 wt %) and dialyzed against deionized water using a 1000 MW cutoff dialysis membrane, at first against the alkaline aqueous solution and then against water, which was changed several times to reach the neutral pH. Dialysis was necessary to hydrolyze pinacol boronic ester units and remove the released pinacol. ^1^H NMR spectrum revealing the molar composition of the synthesized copolymer is presented in the Supporting Information (SI) in Figure S1. *M*_n_ = 43,000 (*Đ* = 1.80) was determined based on the GPC spectrum (Figure S2).

### Solubilization of Clotrimazole within Unimolecular Micelles Based on the Internally Hydrophobized Polyglycidol

A stock solution of clotrimazole (9 mg/mL) in methanol was prepared. A total of 75 mg of each internal hydrophobized polyglycidol was dissolved in 1 mL of methanol. Three milliliters of clotrimazole solution was added to the copolymer solution. The mixture was stirred for 6 h. Subsequently, methanol was allowed to evaporate at 37 °C overnight. The dry polymer–drug content was suspended in 10 mL of deionized water. The suspension was filtered using a 0.8 μm SFCA filter and lyophilized. The amount of drug loaded in the HbPGL-based micelles was determined with ^1^H NMR spectroscopy.

### Drug Release Experiment

A sample of a hydrophobized polymer (HbPGL_BE37 or HbPGL_PC31) containing 1.3 mg of clotrimazole was dissolved in 9 mL of phosphate-buffered saline (PBS) pH = 5.6 and transferred into a regenerated cellulose dialysis membrane (MWCO = 3500) with a magnetic stirrer inside. The dialysis bag was then immersed in 250 mL of PBS pH = 5.6 with 1% of Tween80 (v/v). At given time points, 20 mL of the solution was collected and replaced with 20 mL of fresh PBS/Tween80 solution. Subsequently, 3 × 50 mL of dichloromethane was added to each of the collected samples to extract clotrimazole from the aqueous phase. The organic phases were dried over MgSO_4_ for 30 min while stirring and then liberated from dichloromethane by evaporation under reduced pressure. The dry product was dissolved in 4 mL of acetonitrile and filtered using a 0.2 μm poly(tetrafluoroethylene) (PTFE) filter.

The quantification of the released clotrimazole was determined by an ACQUITY UPLC I-Class chromatography system equipped with a binary solvent pump and a photodiode array detector (Waters Corp., Milford, MA). The separation of an analyte was achieved using an ACQUITY UPLC BEH C18 column (100 × 2.1 mm, 1.7 μm) maintained at a 45 °C temperature. The mobile phase was prepared by mixing 0.1% formic acid (A) and 0.1% formic acid in acetonitrile (B). The elution gradient was 32% B (0–1.0 min), 32–95% B (1.0–3.0 min), 95–95% B (3.0–3.5 min), 95–32% B (3.5–3.52 min), and 32–32% B (3.52–7.0 min). The flow rate was 0.45 mL/min, and the injection volume was 4 μL. The optimal absorption wavelength for clotrimazole was determined and set at 195 nm. The initial stock calibration solution of standards was created with a concentration of 1 mg/mL in acetonitrile.

The stock solution was serially diluted with acetonitrile to obtain working solutions at several concentration levels. The calibration curves were prepared at seven different concentrations of clotrimazole solutions and were linear over a concentration range from 0.78 to 50 μg/mL with a correlation coefficient of >0.999. The system was controlled using MassLynx software (Version 4.1), and data processing (peak area integration, construction of the calibration curve) was performed by a TargetLynx program.

### Cell Culture

Dermal microvascular endothelium cells (HMEC-1) were grown in an MCDB131 medium supplemented with hydrocortisone, l-glutamine, and epidermal growth factor (VEGF). Human cervical cancer endothelial (HeLa) cells were grown in Dulbecco’s modified Eagle’s medium (DMEM). Ten percent fetal bovine serum (FBS) and streptomycin (100 mg/mL) were added to all cell culture media. The cells were grown in T-75 culture flasks at 310 K in an atmosphere containing 5% CO_2_. The cells were subcultured every 2 or 3 days. Cells were harvested and used in experiments after obtaining an 80–90% confluence.

The number of viable cells was determined by the trypan blue exclusion assay with the use of a Countess Automated Cell Counter (Invitrogen, Carlsbad, CA). Cells were seeded in 96-well plates at 1.5 × 10^4^ cells/well in 100 μL of an appropriate medium. After seeding, the plates were incubated for 24 h in a humidified atmosphere containing 5.0% CO_2_ at 310 K to allow cells to attach to the plates.

### Determination of Cytotoxicity

The cytotoxicity study was carried out for neat HbPGL, and its hydrophobized derivatives were obtained by the phenyl moiety incorporation via ester (HbPGL_BE4, HbPGL_BE15, HbPGL_BE20, HbPGL_BE37) or urethane bonds (HbPGL_PC4, HbPGL_PC15, HbPGL_PC31) on the cell viability determined by the usage of the 3-(4,5-dimethylthiazol-2-yl)-2,5-diphenyltetrazolium bromide (MTT) assay.

Briefly, to the 96-well plates containing cells at a density of 1.5 × 10^4^ cells/well in medium, different concentrations (0.1, 1, 10, 50, 100 μM) of all compounds were added. Cells were incubated with the polymers for 24 and 48 h in a 310 K humidified atmosphere containing 5% CO_2_. After the incubation period, cells were washed with 50 μL of phosphate-buffered saline (PBS). Next, 50 μL of a 0.5 mg/mL solution of MTT in PBS was added to each well and cells were further incubated under normal culture conditions for 3 h. After incubation, the residue MTT solution was removed and the obtained formazan precipitate was dissolved in DMSO (100 μL/well). The conversion of the tetrazolium salt (MTT) to a colored formazan by mitochondrial and cytosolic dehydrogenases is a marker of cell viability. Before the absorbance measurement plates were shaken for 1 min and the absorbance at 570 nm was measured on the PowerWave HT Microplate Spectrophotometer (BioTek).

### Statistical Analysis

For statistical significance testing, one-way analysis of variance (ANOVA) for concentration series and post hoc Tukey’s test for pairwise difference testing were used. In all tests, *p*-values <0.05 were considered to be statistically significant. Data are presented as arithmetic mean ± standard deviation (SD). The cytotoxicity values were related to the untreated control (*p < 0.05, **p < 0.01, ***p < 0.005, ****p < 0.0001) as well as between unmodified hyperbranched polyglycidol and modified ones at the same compound concentration (^#^*p* < 0.05, ^##^*p* < 0.01, ^###^*p* < 0.005, ^####^*p* < 0.0001).

### Hydrogel Formation

Hydrogels were prepared by mixing 0.25 mL of an aqueous solution containing 0.05 g of poly(AM-*ran*-2-AAPBA) with 0.15 mL of an aqueous solution containing 0.055 g of each HbPGL-based sample.

### NMR Spectroscopy

^1^H and ^13^C INVGATED NMR spectra were recorded using a Bruker Avance NEO AV 400 MHz. ^1^H DOSY measurements were carried out at 295 K on a Bruker Avance III 500 spectrometer equipped with a 5 mm BBI probe head with a z-gradient coil and a GAB/2 gradient unit capable of producing B0 gradients with a maximum strength of 50 G/cm. The BCU-05 cooling unit, managed by the BVT3300 variable temperature unit, was used for temperature control and stabilization. The spectrometer was controlled with a PC computer running under Windows 7 (64 bit) OS with the TopSpin 3.1 program.

For measurements, each sample was stabilized at 295 K for at least 10 min before data accumulation, and the ^1^H π/2 pulse length was checked and adjusted carefully for each sample. The standard Bruker pulse program dstebpgp3s was selected for measurements using double stimulated echo for convection compensation and LED (Longitudinal Eddy Current Delay) using bipolar gradient pulses for diffusion and three spoil gradients. The shape of all gradient pulses was sinusoidal, the gradient spoil pulse was 0.6 ms, the delay for gradient recovery was set at 0.2 ms, and the LED was set at 5 ms and held constant in all experiments. The gradient pulse (small delta; δ/p30) was kept constant throughout the whole series of temperature measurements, and the diffusion time (big delta; Δ/D20) was changed to achieve the desired signal attenuation at the maximum gradient strength. The DOSY experiments were run in pseudo-2D mode with gradients varied exponentially from 5% up to 95%, typically in 16 steps, with 16 scans per step. Spectra were processed by TopSpin 3.1 software supplied by the spectrometer manufacturer. The 1 Hz line broadening Lorentzian function was applied, and each row was phased and baseline-corrected before executing the Fourier transformation in the F2 dimension. Diffusion coefficient values, for resolved ^1^H signals, were extracted from the T1/T2 analysis module of the TopSpin 3.1 program.

NMR spectra of polymer solutions were recorded at deuterated solvents (DMSO-*d*_6_, MeOD-*d*_1_).

### Gel Permeation Chromatography (GPC)

The average molecular weight of HbPGL was determined with gel permeation chromatography (GPC) using a Shimadzu Pump LC-20AD and a Shimadzu SIL-20A HT Autosampler. A refractometer RI-Optilab-T-rex-Wyatt and a laser photometer DAWN 8+ (Wyatt Technology) were used as detectors. *N*,*N*′-Dimethylformamide was used as an eluent at a flow rate of 0.8 mL/min at 25 °C. *M*_w_ = 7800, *M*_w_/*M*_n_ =1.70.

### Differential Scanning Calorimetry (DSC)

DSC analysis of polymers was performed on a 2920 modulated DSC (TA Instruments) at a heating and cooling rate of 10 °C/min. Samples were annealed at 100 °C before the cooling/heating loop was applied.

### Rheology

Gel formation was confirmed with oscillation frequency sweep tests carried out in the linear viscoelastic regime using a parallel plate–plate geometry of 8 mm diameter with a 0.3 mm gap on a Thermoscientific HAAKE MARS 40 rheometer. A strain sweep measurements for the hydrogel samples were performed at a frequency of 1 Hz in the range of strain from 0.02 to 2000%. Frequency sweep tests were carried out at 10, 25, and 40 °C in the linear viscoelastic regime. Temperature sweep tests in the range from 10 to 50 °C were performed at a frequency of 1 Hz and 1% of strain using the continuous heating program with a heating rate of 5 °C/min.

## Results and Discussion

### Hydrophobization of Hyperbranched Polyglycidol

For the study focused on the influence of hydrophobization of the hyperbranched polyglycidol core on the properties of synthesized macromolecules and their hydrogels, we applied a polymer where the molecular weight determined based on GPC was 7800 (DP*_n_* = 105). HbPGL polymers of such molecular weight are routinely obtained *via* anionic polymerization of glycidol conducted in bulk.^25^ The total molar fraction of linear units (L_13_ and L_14_) in the interior of HbPGL (Scheme 1) bearing monohydroxyl moieties, which can be hydrophobized, was 0.40. The process of HbPGL core hydrophobization required the protection of 1,2-diol moieties of terminal units (33 mol % of all repeating units) to avoid the modification of the macromolecular corona. It was achieved by the protection of diol groups in the form of acetals in the reaction with solketal catalyzed with PTSA. We synthesized a set of HbPGLs differing in a number of phenyl moieties incorporated *via* ester or urethane bonds in the macromolecular core, applying benzoyl chloride or phenyl isocyanate (Scheme 1), respectively. The characteristics of hydrophobized HbPGLs with carboxyphenyl and phenyl carbamate groups, respectively, are given in Tables 1 and 2, respectively.

The covalent immobilization of phenyl units within the HbPGL core was confirmed based on ^1^H, ^13^C INVGATED NMR, and ^1^H DOSY NMR spectroscopy in DMSO-*d*_6_ (Figures S3–S70). The degree of hydrophobization of monohydroxylated repeating units was determined for HbPGL acetals based on the comparison of the integration of dimethylacetal groups of terminal units (in the range between 1.15 and 1.35 ppm) with the integration of phenyl protons at the chemical shift in the region from 7.30 to 8.10 ppm for ester (Figures S3–S11) and from 6.75 to 7.60 ppm in the case of urethane derivatives (Figures S39–S46). In the case of HbPGL hydrophobized via urethane bonds, the conversion was also confirmed by signals at 9.65 ppm coming from NH protons of urethane bonds in the ^1^H NMR spectrum (Figures S39–S46). Deprotection of diol groups in the terminal units for both ester and urethane derivatives was confirmed based on ^1^H and ^13^C NMR spectra (Figures 1, S12–S29, and S47–S62). In addition, the ^1^H DOSY NMR analysis of deprotected HbPGL derivatives showed that the values of the diffusion coefficients of protons corresponding to the aromatic protons (in the region 6.80–7.80 ppm for urethane derivatives and 7.20–8.10 ppm for ester derivative) and protons of HbPGL backbone (3.20–4.00 ppm) were the same, which confirmed that all phenyl moieties are covalently immobilized via ester/urethane linkages with HbPGL and that all modified polymers were free of unreacted compounds (Figures S30–S38 and S63–S70). The detailed analysis of ^13^C INVGATED NMR spectra of hydrophobized HbPGLs revealed the reduction of the integration of signals corresponding to carbon atoms of linear units, i.e., L_13_ and L_14_ in comparison to neat HbPGL, along with the appearance of signals of carbonyl groups that reacted with both primary and secondary alcohols in L_13_ and L_14_ units, respectively, in the case of functionalization with benzoyl chloride and phenyl isocyanate.

**Figure 1:**
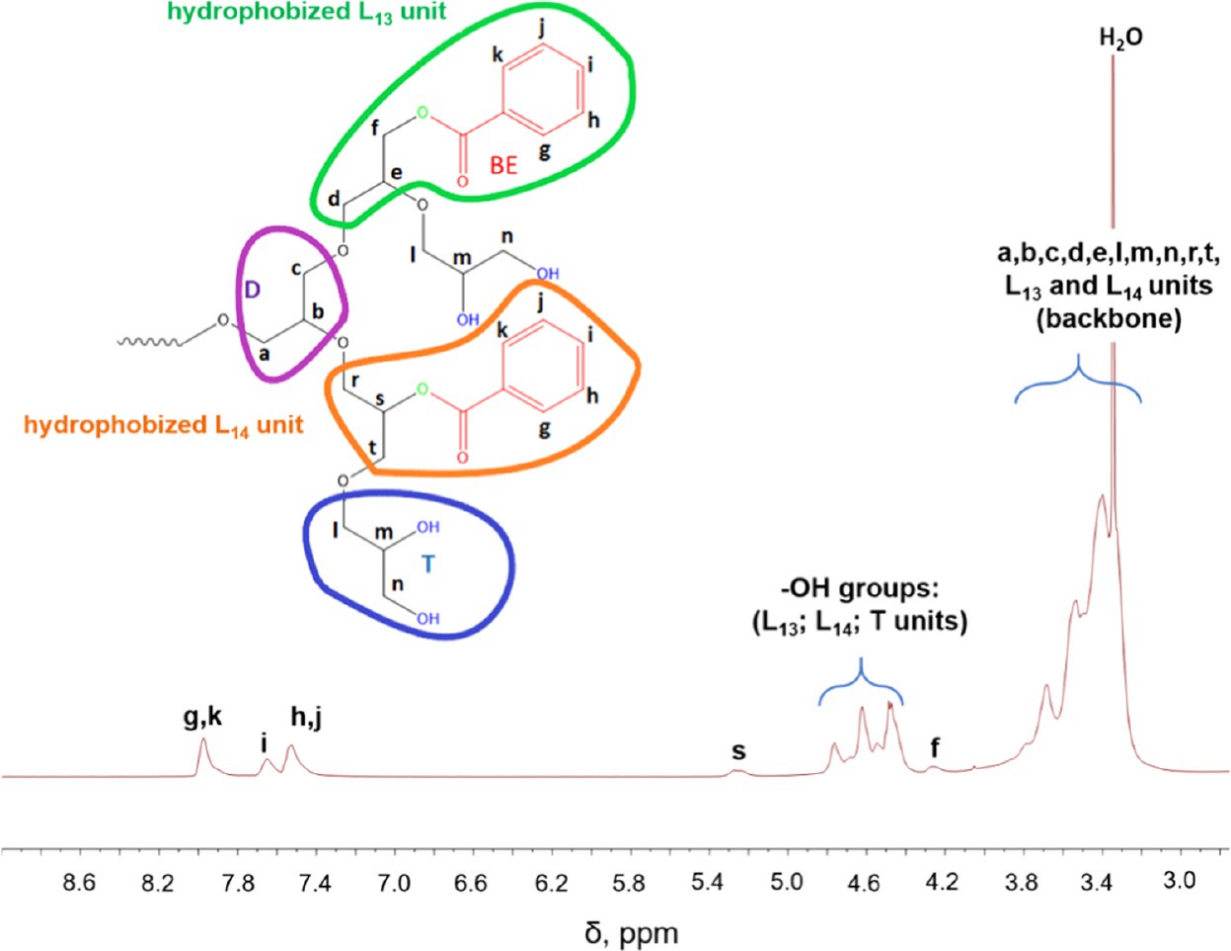
Exemplary ^1^H NMR spectrum of the internally hydrophobized HbPGL with phenyl moieties incorporated *via* ester bonds (HbPGL_BE26) recorded in DMSO-*d*_6_.

The degree of HbPGL’s internal OH group hydrophobization with phenyl units *via* ester or urethane bonds ranged from 4 to 82 mol %. Generally, the hydrophobized HbPGLs with phenyl moieties incorporated via urethane linkages turned out to be less soluble in water. For example, HbPGL_PC66 was completely insoluble in water, whereas HbPGL_BE81 was partially soluble.

DSC analysis of all hydrophobized HbPGLs with phenyl carbamate and phenyl ester groups revealed significant changes in the glass transition values, *T*_g_, in comparison to the neat polymer (*T*_g_ = −29.9 °C). *T*_g_ increased with the increase of the hydrophobization degree of HbPGL; however, these changes were more significant for urethane derivatives. For instance, *T*_g_ for HbPGL_BE15 was equal to −26.4 °C, whereas for HbPGL_PC16, it was approximately 10 °C higher. A higher degree of HbPGL hydrophobization with the phenyl carbamate groups led to a further increase in the *T*_g_ of synthesized polymers, reaching positive Centidegrees values. This behavior can be ascribed to the fact that urethane linkages are prone to form hydrogen bonds, which is especially favorable in low-temperature range^34,35^ and thus leads to the restriction of segmental motions in wider temperature range and thus stiffening of HbPGL_PC macromolecules.

### Clotrimazole Encapsulation and *In Vitro* Release Study

Clotrimazole, which has three unconjugated phenyl rings and an imidazole moiety in the structure and is routinely used in the treatment of candidiasis caused by *Candida albicans* and other *Candida* species, was used to assess the ability of HbPGL with varying contents of phenyl groups to solubilize hydrophobic drugs. Its poor solubility in water (0.49 μg/mL)^36^ requires the development of polymer components that enhance its solubility and thus increase its bioavailability and, as a result, boost the therapy efficiency.

The process of clotrimazole solubilization was performed according to the ultrasound-assisted solvent evaporation method. The encapsulation efficiency (EE) was estimated using ^1^H NMR spectroscopy.

To assess the effect of the hydrophobization of the HbPGL core on the solubilization of clotrimazole into unimolecular micelles based on HbPGL, we applied unmodified HbPGL as a reference, in which no clotrimazole molecules could have been encapsulated. HbPGLs with a low degree of hydrophobization (4 and 15 mol % of modified monohydroxyl constitutional units) via both ester and urethane linkages were virtually unable to encapsulate the drug (Table 3). The encapsulation efficiency, EE, was below 1%. Upon suspending the polymer/drug mixture in water, clotrimazole precipitated and was removed in the filtration process. This behavior can result from the fact that a low number of phenyl units randomly distributed in the HbPGL core does not provide enough hydrophobic environment to capture the highly hydrophobic clotrimazole.

**Table 3:** Results of Clotrimazole Solubilization, i.e., Drug Loading and Encapsulation Efficiency, EE, within HbPGL, Where the Core Was Internally Hydrophobized with Phenyl Moieties Incorporated via Ester or Urethane Linkages, Respectively

polymer	drug loading (mg/g)	EE (%)
**HbPGL_BE4**	2.10	0.3
**HbPGL_BE15**	2.95	0.5
**HbPGL_BE27**	10.4	2.6
**HbPGL_BE37**	210	55.0
**HbPGL_BE49**	259	71.4
**HbPGL_BE58**	473	88.8
**HbPGL_BE74**	nonfiltrated structures	
**HbPGL_BE81**	nonfiltrated structures	
**HbPGL_PC4**	0.48	0.1
**HbPGL_PC16**	3.50	0.8
**HbPGL_PC31**	386	67.2
**HbPGL_PC55**	nonfiltrated structures	

A substantial amount of encapsulated drugs have been observed for HbPGLs, in which at least 30 mol % of monohydroxyl units were hydrophobized. In the case of ester derivatives upon the increase of the degree of hydrophobization in the range from 37 to 58 mol %, the drug loading increased gradually from 210 to 473 mg per gram of polymer, with the encapsulation efficiency between 55 and 90%. Among all of the synthesized urethane derivatives, only in the case of HbPGL_PC31, the process of encapsulation was effective (EE = 67.2%) with drug loading equal to 386 mg/g. The hydrophobization of the HbPGL core in the range of 74–81 mol % in the case of ester derivatives and for urethane derivatives at the degree of modification equal to 55 mol % turned out to be excessive as the encapsulation process led to the formation of aggregates, where filtration was impossible. Based on the drug encapsulation experiments, we can conclude that HbPGLs hydrophobized via urethane bonds displayed a higher ability of clotrimazole solubilization as the drug loading achieved for HbPGL_PC31 was higher in comparison to the ester analog. This behavior can be explained by the possibly formed hydrogen bonds between urethane bonds present in the polymer and the imidazole of clotrimazole.

**Figure 2:**
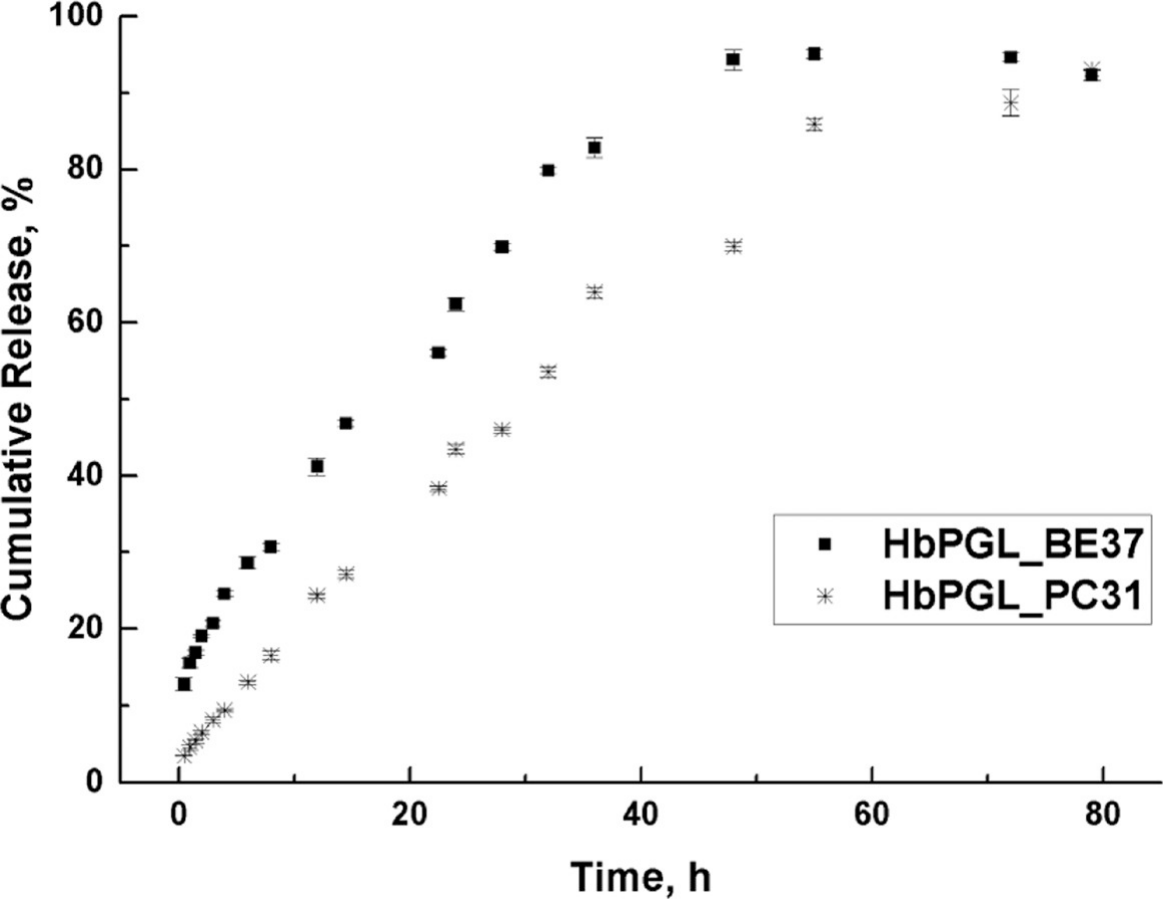
Clotrimazole release profile from HbPGLs hydrophobized with phenyl moieties immobilized via ester (HbPGL_BE37) or urethane (HbPGL_PC31) bonds.

To evaluate the effect of a chemical bond by which a phenyl moiety was immobilized within the HbPGL structure, we investigated clotrimazole release from benzoate (HbPGL_BE37) and phenyl carbamate (HbPGL_PC31) derivatives (Figure 2). These systems displayed a comparable degree of hydrophobization and significant drug loading. Generally, both systems displayed gradual drug release; however, a rapid initial burst release (approx. 15% after 0.5 h) was only observed in the case of the ester derivative. Moreover, a slower release rate of clotrimazole was observed for the urethane derivative. For instance, 50% of cumulative drug release was observed after 28 h for the urethane HbPGL derivative, while in the case of the ester derivative, this release level was attained after 18 h. These data indicate a higher affinity of clotrimazole toward the phenyl carbamate HbPGL matrix.

### Cytotoxicity Measurements

**Figure 3:**
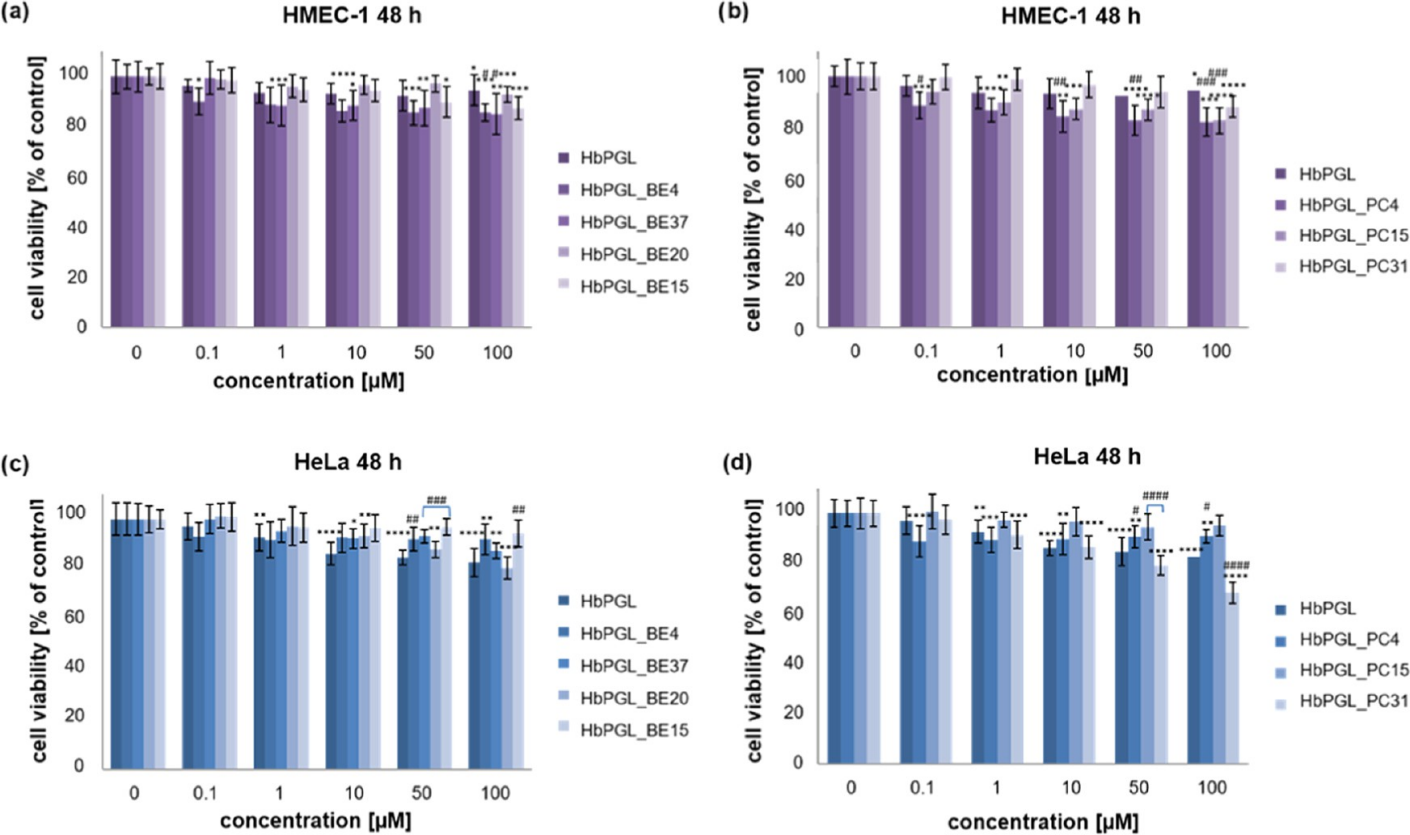
Influence of the molar concentration of the neat hyperbranched polyglycidol and its hydrophobized derivatives on the cell viability of HMEC-1 (a, b) and HeLa (c, d) after 48 h of incubation at 37 °C. Data are presented as a percentage of control (untreated cells) standard deviation (SD). The cytotoxicity values compared to the untreated control (*p < 0.05, **p < 0.01, ***p < 0.005, ****p < 0.0001) as well as between unmodified hyperbranched polyglycidol and modified ones at the same compound concentration (^#^*p* < 0.05, ^##^*p* < 0.01, ^###^*p* < 0.005, ^####^*p* < 0.0001).

The cytotoxicity assay was carried out for neat HbPGL and its phenyl-enriched derivatives using the MTT test on non-neoplastic (dermal microvascular endothelium cells, HMEC-1) and neoplastic (human cervical cancer endothelial, HeLa) line cells. The viability of both HMEC-1 and HeLa cells was evaluated after 24 and 48 h of incubation at 37 °C (Figures S71 and 3, respectively). As shown in Figure 3, even at the highest concentration of each polymer used of 100 μM, no significant decrease in HMEC-1 and HeLa cell viability was observed even after 48 h of incubation. The lack of cytotoxicity of such high concentrations of polymer samples is important due to hydrogel formulations, which makes these polymers prospective from the point of view of biomedical applications.

### Influence of Temperature on the Rheological Properties of Hydrogels Constructed of Internally Hydrophobized Hyperbranched Polyglycidol and Acrylamide Copolymer Equipped with 2-Acrylamidephenylboronic Acid, Poly(AM-*ran*-2-AAPBA)

**Scheme 2:**
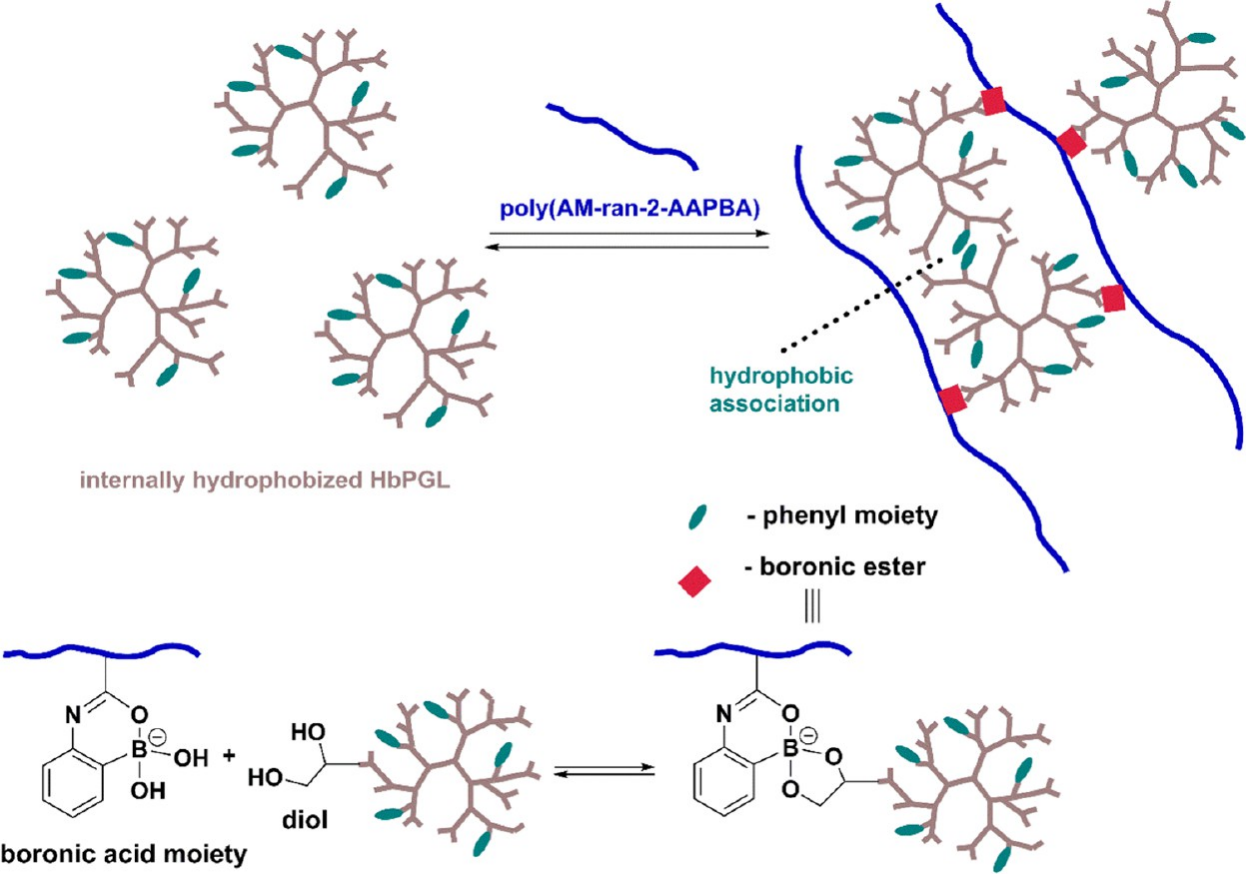
Illustration Depicting the Mechanisms of the Hydrogel Formation Based on the Internally Hydrophobized HbPGL and Poly(AM-*ran*-2-AAPBA)

The hydrogel constructed of neat hyperbranched polyglycidol cross-linked with acrylamide copolymer equipped with 2-acrylamidephenylboronic acid moieties, poly(AM-*ran*-2-AAPBA), is a reversible network thanks to the dynamic equilibrium between boronic acids, diols, and formed boronic ester species. It is known that the networks constructed of boronic ester cross-links are thermoresponsive.^2,15,16^ It results from the fact that the formation of boronic esters is an exothermic process,^16,37^ and thus the increase in temperature is not favorable for the network tie-points that assure the network integrity. The gradual heating of the neat HbPGL-based hydrogel from 10 to 50 °C was accompanied by a consecutive decrease of storage modulus *G*′ and then dropped below *G*″ at approximately 40 °C (Figure S72), which corresponds to the transition of the hydrogel to the liquid state as a result of the equilibrium shift to substrates, i.e., boronic acids and diols (Scheme 2).

**Figure 4:**
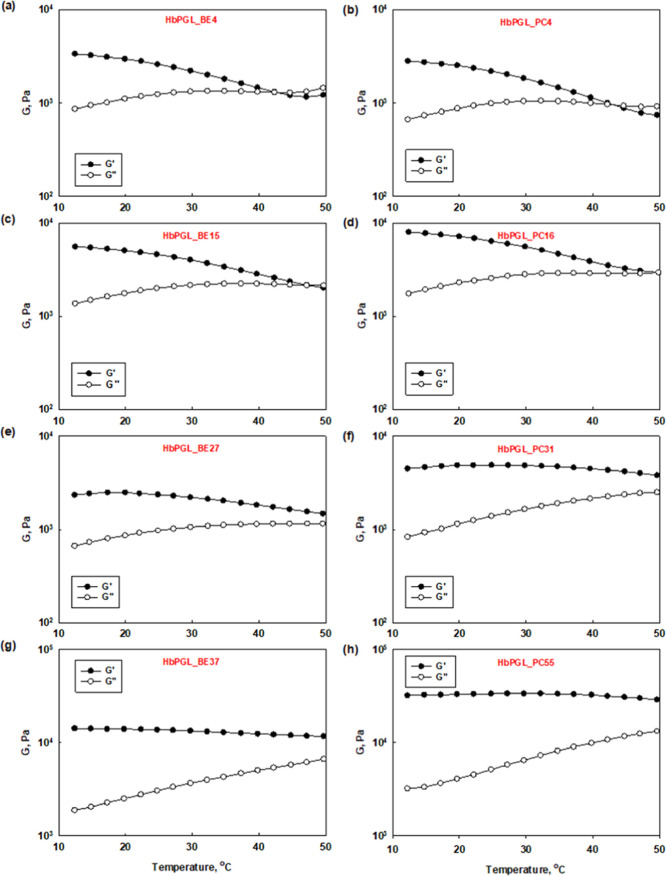
Dependence of *G* moduli on temperature for hydrogels constructed of hydrophobized HbPGL by the incorporation of phenyl moieties *via* ester or urethane bonds.

The incorporation of aromatic groups in the HbPGL’s core resulted in significant changes in the thermal behavior of constructed hydrogels with poly(AM-*ran*-2-AAPBA) (Figure 4). The higher the degree of hydrophobization of HbPGL used for the construction of hydrogel, the higher gel–liquid transition temperature was observed (*G*′ < *G*″) (Figure 4). Regardless of whether phenyl rings were incorporated via ester or urethane linkages, the comparable thermal behavior of hydrogels constructed of macromolecules at a certain hydrophobization degree was observed. For hydrogels composed of HbPGL, where approx. 30 mol % of monohydroxyl groups were hydrophobized via ester linkages (HbPGL_BE27), and HbPGLs, where less than 30 mol % of monohydroxyl groups were modified via urethane bonds, the crossover of storage and loss moduli (*G*′ = *G*″) was observed above 50 °C. In the case of hydrogels built of HbPGL with a higher degree of hydrophobization, i.e., starting from HbPGL_PC31 and HbPGL_BE37, *G*′ was higher than *G*″ in the whole investigated temperature range from 10 to 50 °C. Moreover, the difference between *G*′ and *G*″ values at the low-temperature range increased along with the increase of the hydrophobization degree of applied HbPGL for the gel construction. For example, at 15 °C, tan δ for the hydrogel systems constructed of HbPGL hydrophobized at 15 mol % was approximately 0.25, whereas for HbPGL_PC55, tan δ was equal to 0.10. This behavior inputs about the higher contribution of solid-like behavior in the case of hydrogels made of hydrophobized HbPGL in comparison to the hydrogel based on the neat HbPGL.

### Viscoelastic Properties of Hydrogels Constructed of Internally Hydrophobized Hyperbranched Polyglycidol and Poly(AM-*ran*-2-AAPBA)

**Figure 5:**
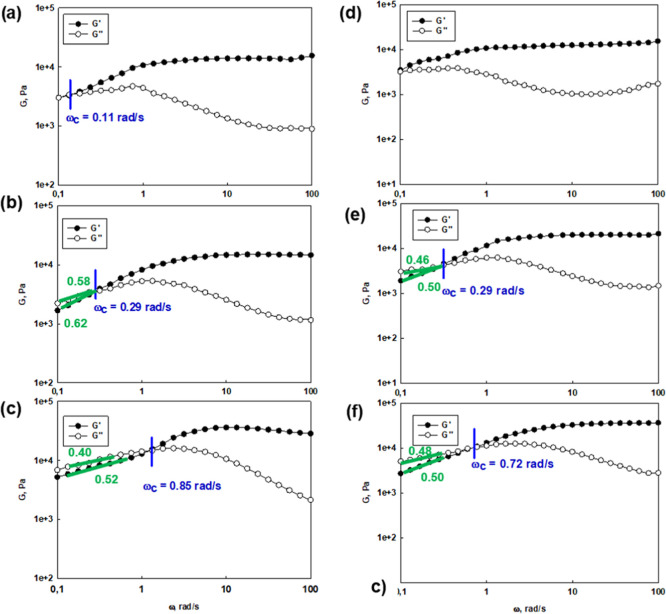
Frequency sweep tests performed for hydrogel constructed of poly(AM-*ran*-2-AAPBA) cross-linked with HbPGL_BE37 recorded at 10 °C (a), 25 °C (b), and 40 °C (c) and HbPGL_PC55 at 10 °C (d), 25 °C (e), and 40 °C (f).

**Figure 6:**
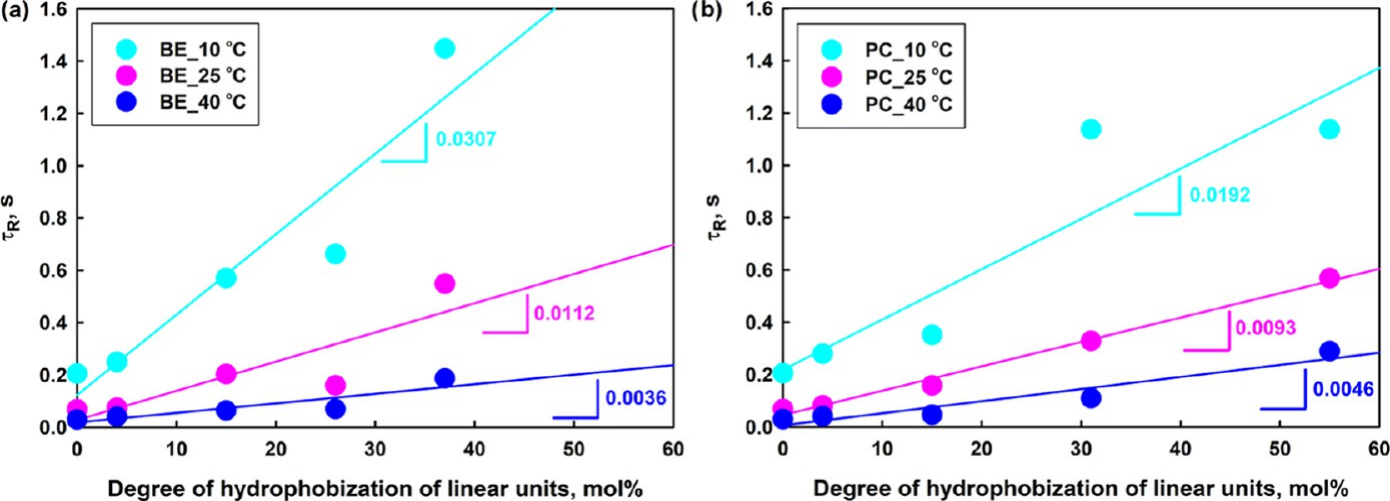
Dependence of the relaxation time of macromolecules in the networks, τ_R_, on the degree of hydrophobization using ester (a) and urethane (b) linkages.

All hydrogel systems composed of both neat and hydrophobized HbPGLs are viscoelastic networks, which were revealed based on the frequency sweep experiments (Figures 5 and S73–S76). The study was carried out at 10, 25, and 40 °C. In the higher frequency range, i.e., shorter time scales, the storage modulus exceeded the value of the loss modulus (*G*′ > *G*″). It results from the fact that the lifetime of boronic ester cross-links was longer in comparison to the applied strain. The decrease of frequency resulted in the inversion of *G*′ and *G*″ at the crossover frequency (ω_c_), which corresponds to the gelation point (gel–liquid transition) as an effect of the cross-link dissociation. ω_c_ denotes the onset of macroscopic chain displacement, while at frequencies below ω_c_ (longer time scales), the material begins to flow (*G*′ < *G*″) due to the dominant contribution of liquid-like behavior. The crossover frequency, ω_c_, for hydrogels constructed with hydrophobized HbPGL was lower at each investigated temperature in comparison to the hydrogel based on the neat HbPGL. For example, ω_c_ for neat HbPGL-based hydrogel at 10 °C was equal to 0.78 rad/s, whereas for hydrogels constructed of HbPGL_BE37 and HbPGL_PC55, it was reduced to 0.10 and 0.14 rad/s, respectively. At 40 °C, the crossover frequency for neat HbPGL-based hydrogel was 5.30 rad/s, whereas for HbPGL_BE37- and HbPGL_PC55-based networks, ω_c_ was significantly shifted to 0.85 and 0.55 rad/s, respectively. The slope of ω_c_ dependence on temperature for the hydrogel constructed of neat HbPGL was 0.151 (Figure S73d), whereas the slope for this dependence for the hydrogels composed of HbPGL_BE37 and HbPGL_PC55 was 0.025 and 0.014 (Figure S77), respectively. The decrease of crossover frequency observed for hydrogels composed of hydrophobized HbPGL (Figures 5 and S73–S76) was directly related to the longer relaxation times of macromolecules engaged in the network formation according to the following relationship: ω_c_ = 1/2π·τ_R_, where τ_R_ is a relaxation time of macromolecules.^38^ This behavior can be attributed to the presence of an additional cross-linking mechanism, beside boronic esters, in the network. As comparable values of ω_c_ were obtained for hydrogels constructed of both urethane and ester derivatives at the same modification degree, we rejected a contribution of H-bonding of urethane bonds as a dominant effect in the network formation. Thus, we concluded that hydrophobic interactions generated by phenyl moieties are responsible for prolonging the relaxation time of macromolecules (Figure 6a,b), and besides boronic ester cross-links are engaged in the network formation (Scheme 2). It is noteworthy that the slope of ω_c_ dependence vs temperature decreased with the increase of the degree of hydrophobization of HbPGL macromolecules applied for the gel construction (Figure S77). These data input, however, that cross-links based on hydrophobic associations are less sensitive to temperature, which is consistent with literature data.^31^

Activation energy, *E*_a_, determined for all hydrogels based on the Arrhenius equation (Figure S78) revealed that higher energy is needed to activate the relaxation processes of macromolecules in the networks composed of hydrophobized HbPGL than that constructed of neat HbPGL (Table 4).

**Table 4:** Activation Energy, *E*_a_, Values of the Macromolecular Relaxation in the Network Determined for Hydrogels Constructed of Neat and Hydrophobized HbPGL

hydrogel sample	*E*_a_ (kJ/mol)
**neat HbPGL**	47.2
**BE4**	44.6
**BE15**	53.2
**BE26**	55.2
**BE37**	50.0
**PC4**	47.2
**PC15**	66.4
**PC31**	57.2
**PC55**	

The presence of an additional mechanism of cross-linking was also confirmed based on the increased value of the frequency-independent plateau of *G*′ = *f*(ω), (*G*_N_) in the higher frequency range above ω_c_, i.e., in the region of shorter time scales. The increasing *G*_N_ modulus of hydrogels based on the hydrophobized HbPGL is ascribed to the increasing number density of elastically effective network chains, *N*_Eexp_, describing the following dependence: *G*_N_ = *N*_E_exp__*kT*,^39^ and thus the increase of the strength of the hydrogel. *G*_N_ was the lowest for the hydrogel composed of neat HbPGL and gradually increased with the increase of hydrophobization degree of HbPGL applied for the hydrogel formation (Figure S79). These data input that the enrichment of HbPGL with a low fraction of phenyl groups can lead to the formation of a single-sticker-type of intermolecular interactions thanks to the high probability of homogeneous distribution of hydrophobic rings within sphere-shaped HbPGL. The significant increase of *G*_N_ for hydrogels constructed of substantially hydrophobized HbPGL probably results from the formation of phenyl group clusters (aggregates).^40,41^ Moreover, along with the increase of the hydrophobization degree of HbPGL applied for the hydrogel formation, the hydrogels were more opaque, although initial polymer solutions applied for the hydrogel formation were transparent.

The formation of hydrophobic associations in water is an effect of the dehydration of parts of macromolecules, which is associated with a dominant entropic contribution to the self-assembly in the pure aqueous environment.^42−45^ The probability of hydrophobic interactions between individual macromolecules increases with the increase of the hydrophobization degree of HbPGL. The gradual increase of *G*_N_ of the hydrogels along with the hydrophobization degree of applied HbPGL can be ascribed to the homogeneous distribution of phenyl units within the hyperbranched macromolecules, which consecutively are engaged in the network formation. Moreover, these data indicate that hydrophilic HbPGL corona swollen with water does not constitute efficient protection against intermolecular hydrophobic interactions in the polymer network.

**Figure 7:**
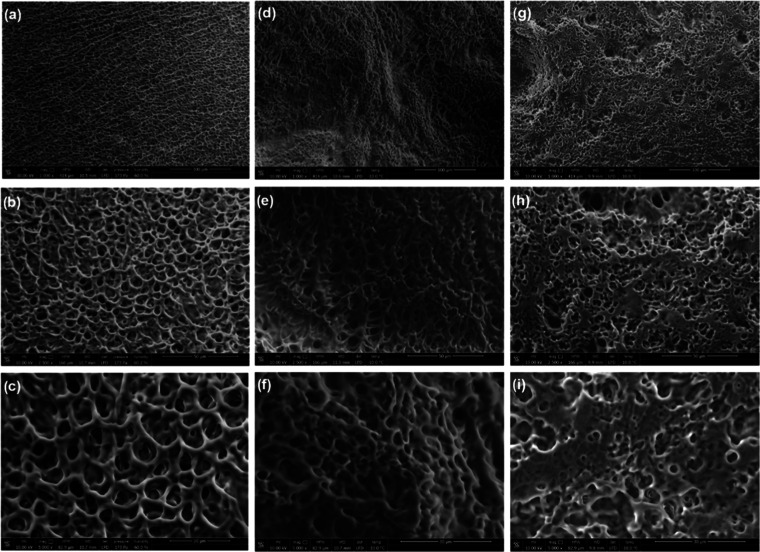
ESEM images of hydrogel systems constructed of neat HbPGL (a–c), HbPGL_BE26 (d–f), and HbPGL_PC55 (g–i).

The ESEM images recorded for hydrogels constructed of the neat HbPGL and hydrophobized HbPGL differing in the number of phenyl groups (Figure 7) revealed the significant changes in their morphology. In the case of neat HbPGL-based hydrogel, the structure of the network was homogeneous and highly porous. A slightly reduced porosity was observed for the network of the moderately hydrophobized HbPGL (HbPGL_BE26); however, the network porosity was still uniformly porous. On the contrary, in the morphology of the hydrogel constructed of HbPGL_PC55, the unporous patches were distinguished, which can be ascribed to the unhydrated areas of the material. The decreasing porosity of hydrogels along with the increasing molar fraction of hydrophobic units in applied HbPGLs results from the diminishing mesh size (ξ) of the network according to the relationship: ξ = (*N*_E_exp__)^−1/3^.^46^

The contribution of both cross-linking mechanisms, i.e., boronic esters and hydrophobic associations, should vary with temperature. It is well known that the equilibrium of reaction leading to boronic ester formation upon heating from 10 to 40 °C is shifted to the substrate direction, as the process of their formation is exothermic. Since *G*_N_ did not decrease upon heating (Figures 5 and S73–S76) and even increased at 40°C, it can be concluded that the contribution of hydrophobic interactions increases along with temperature in the studied range.^47^

It is noteworthy that frequency sweep experiments revealed that *G*′ does not cross *G*″ in its maximum value, which was even more evident for hydrogels composed of HbPGL, where the core was highly enriched with hydrophobic units. Such behavior indicates that more than one mechanism of cross-linking is responsible for the network formation.^15^ In the case of hydrogel composed of neat HbPGL, this effect was observed, however, to a lower extent and can be ascribed to boronic ester cross-links and physical entanglements of high-molecular-weight macromolecules of acrylamide copolymer. For hydrogels built of hydrophobized HbPGLs, the formation of hydrophobic clusters contributed to the network formation, apart from these abovementioned cross-linking factors. In the case of HbPGL macromolecules cross-linked via boronate linkages using low-molecular boronic acids, the maximum of *G*″ was crossed by *G*′, which was ascribed to only one mechanism governing the network formation, as the used HbPGL was lack of physical entanglements.^16,48^

Predominant values of *G*″ over *G*′ in the region of low frequencies (longer time scales) below the crossover frequency indicate the onset of a structural change in the network, which can be ascribed to viscous flow. The difference between *G*′ and *G*″ values for the hydrogels built of the hydrophobized HbPGL, however, was lower in comparison to the hydrogel composed of neat HbPGL and decreased upon the gradual enrichment of HbPGL with phenyl units (Figures 5 and S73–S76). Tan δ determined at 10 °C for hydrogels based on hydrophobized HbPGLs at the degree from 4 to 55 mol % ranged from 0.30 to 1.00, respectively, whereas for the neat-based HbPGL hydrogel, tan δ was equal to 0.27. Moreover, in the case of hydrophobized hydrogels, the slope of *G*′ and *G*″(ω) in the region of low frequency, below ω_c_, was flattened at each investigated temperature (Figures 5 and S73–S76). This behavior can be explained by the still present transient cross-links, which prolong the relaxation of macromolecules.^49−51^ Therefore, the deviation of the relaxation process from a single-exponential Maxwell model (*G*′ ∼ ω^2^ and *G*″ ∼ ω^1^) for hydrophobized HbPGL-based hydrogels was observed. In the case of the hydrogel constructed of the neat HbPGL, the relaxation process that follows the Maxwell model was merely observed at 40 °C (Figure S73c), whereas at lower temperatures the slope of moduli was decreased (Figure S73a,b) as an effect of the increased stability of boronic ester cross-links at lower temperatures. In addition, the polydispersity of applied polymer components can cause multiexponential decay that leads to the broadening of the relaxation time spectrum.^49^ The incorporation of phenyl units into HbPGL, however, more evidently influenced the flow of the samples as the deviation from the Maxwell model was more significant along with the increase of the hydrophobization degree of HbPGL applied for the hydrogel formation (Figures 5 and S73–S76). In the case of the hydrogel composed of HbPGL_PC55, below ω_c_, the values of both *G*′ and *G*″ moduli were comparable, and the relaxation process significantly deviated from the Maxwell model. The detailed analysis of the behavior of moduli in the low-frequency region revealed that hydrogels constructed of the hydrophobized HbPGL do not flow as easily as the systems based on the neat HbPGL. The gradual reduction of the slope of *G*′ and *G*″ in the low-frequency region along with the increase of the network hydrophobization can result from the formation of larger sticker aggregates of hydrophobic domains, referred to as clusters with a higher energy barrier for the dissociation of such complex sticker.^52^ As a result, the relaxation time spectrum was broadened, and thus the flow behavior, typical for the liquid-like behavior, is diminished.

Strain sweep tests carried out for the prepared hydrogels revealed that the degree of hydrophobization of used HbPGL significantly influences the stability of the network against the applied strain (Figures S80 and S81). At the low amplitude range, both storage and loss moduli exhibited a plateau, characteristic for the linear viscoelastic region, which was followed by a decrease of both moduli at amplitude characteristic for each hydrogel. Generally, the value of strain, at which the integrity of the network was disrupted, decreased with the increase of the hydrophobization degree of HbPGL applied for the hydrogel formation. It evidently indicates that phenyl rings incorporated into the HbPGL core influence the mechanical properties of the constructed hydrogels. After exceeding the strain value, at which the network is disrupted, the inversion of *G*′ and *G*″ was observed, and *G*″ became larger than *G*′. The crossover of both moduli corresponds to a transition from a solid (hydrogel) to a liquid state and the material exhibits a viscous flow. In the case of the hydrogel systems built of HbPGL_BE37 or HbPGL_PC31, as low as 10% of strain was needed to trigger the gel to sol transition. In the case of hydrogels composed of HbPGL where less than 30 mol % of all monohydroxyl groups were hydrophobized via either ester or urethane bonds, the strain required for the disruption of the network was close to 100%. Hydrogels composed of neat HbPGL or low-hydrophobized macromolecules (HbPGL_BE4 or HbPGL_PC4) were disrupted at strain above 100%. These data evidently demonstrate that the reorganization of hydrophobic cross-links generated between individual macromolecules is restricted. The significant contribution of hydrophobic interactions in the case of hydrogels built of highly hydrophobized HbPGLs shaded the effect of rapid and continuous reorganization of dynamic boronic ester cross-links.

In addition, although the concentration of applied polymer components in all investigated hydrogel systems was the same, for samples prepared from HbPGL_BE37, HbPGL_PC31, and HbPGLPC55 macromolecules, the phenomenon of water-repelling was observed with a naked eye. This behavior can be ascribed to the significant hydrophobization of the HbPGL core, which causes the increase in the probability of hydrophobic interactions between individual macromolecules. The formation of local hydrophobic domains is assisted by an entropy gain as a result of the subsequent release of unfavorably organized water molecules from the intramolecular spaces.^42−45^

**Figure 8:**
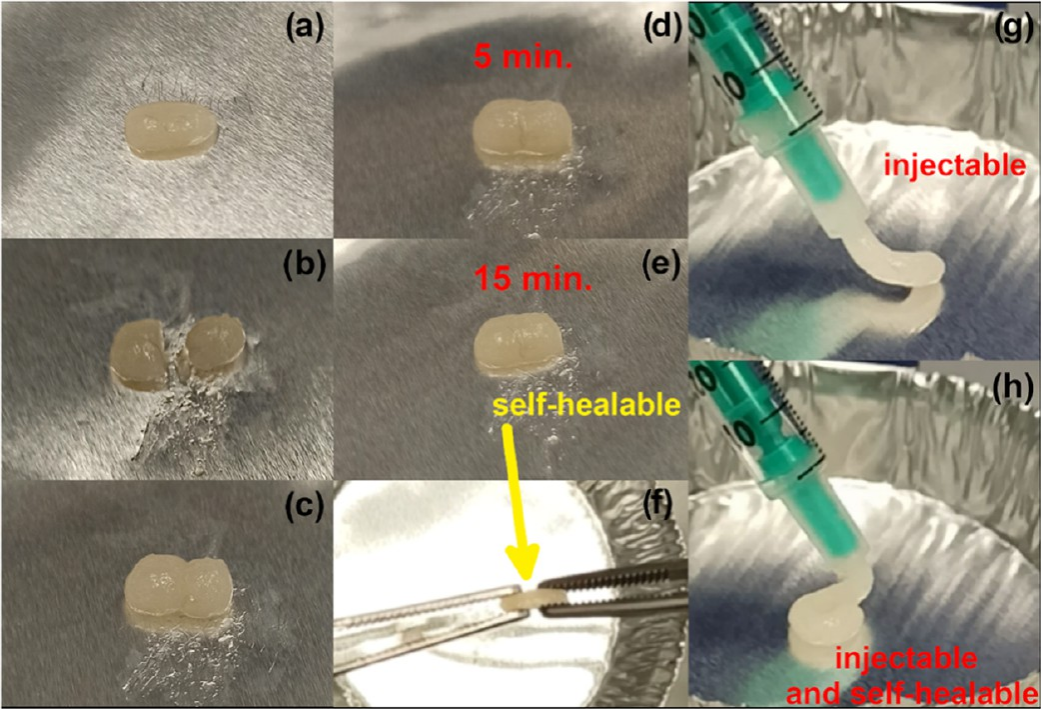
Illustration of the experiment demonstrating the self-healable behavior of HbPGL_BE26-based hydrogel. The hydrogel sample (a) was ruptured into two pieces (b) and placed in close contact (c). The sample was gradually repaired after 5 min (d) and 15 min (e), respectively, and then the sample was increased with a couple of tweezers (e), which revealed that the sample was self-healable under ambient conditions (f). The hydrogel was injected via a syringe (g). The hydrogel after extrusion from the syringe preserves its integrity (h). In addition, a video file presenting an experiment of the hydrogel injection via a needle was attached.

The participation of hydrophobic moieties in the network formation significantly influences the self-healing ability of the prepared hydrogels. The self-healing properties of the hydrogel are mainly determined by two factors. From one side, the recombination rate of the binding sites is crucial, whereas, from the other side, the sufficient chain dynamics of the individual macromolecules is indispensable to enable mobility of the interacting sites to ensure the cross-link reformation.^38^ The reforming ability of boronic ester cross-links in the neat HbPGL-based network is assured not only by a sufficient rate of exchange between the product (boronic ester) and substrates, i.e., boronic acid and 1,2-diol (Scheme 2), but also by the high mobility of HbPGL macromolecules in the network, which assures that binding sites can meet once again.^2,15^ The relaxation time (τ_R_) of macromolecules engaged in the network formation gradually decreased with the increase of the hydrophobization degree of the applied HbPGL. These data input that the highly hydrophobized HbPGL macromolecules are significantly constrained in the network, and in spite of the presence of dynamic boronic ester-based cross-links, the self-healable properties of hydrogels are reduced. This behavior can be explained by the restricted ability of hydrophobic associations to reorganize. The increasing contribution of hydrophobic interactions diminishes the significance of the dynamic boron cross-links in the network, and thus self-healing properties are gradually reduced. For example, the hydrogel constructed of HbPGL_BE26 and HbPGL_PC31 still retained the self-healing properties (Figure 8); however, the time needed to vanish the fracture between two portions of the gel was slightly longer in comparison to the behavior of the hydrogel based on the neat HbPGL (Figure S82). The hydrogels constructed on HbPGLs with the highly hydrophobized interior, i.e., HbPGL_BE37 and HbPGL_PC55, did not display self-healable properties. The exemplary self-healing test performed for the hydrogel based on HbPGL_PC55 (Figure S83) revealed that two hydrogel pieces placed at a close distance were not able to reform one piece of the hydrogel despite a long time.

The self-healing properties of hydrogels are important in biomedical applications, for example, in topical therapies. Both the reduced self-healing ability and the flow behavior of the hydrogel result in the limited ability of the formation of continuous coverage with the hydrogel-based drug carrier on the afflicted area, which can influence their potential in biomedical applications. The formation of a uniform layer is of great importance to assure effective tissue coverage to attain controlled drug delivery.

**Scheme 3:**
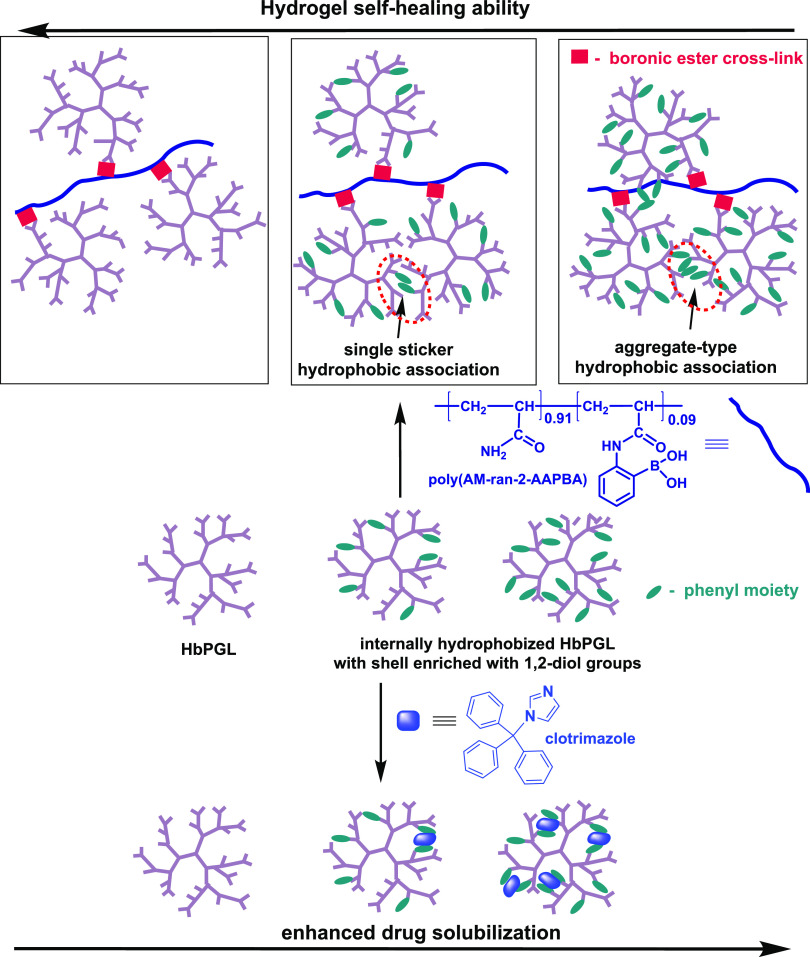
Illustration Showing the Influence of the HbPGL Hydrophobization with Phenyl Moieties on the Solubilization Ability of Clotrimazole and the Rheological Properties of Hydrogel Platforms

The scope of the article is summarized in Scheme 3.

## Conclusions

We have successfully demonstrated the construction of injectable hydrogels composed of unimolecular micelles based on hyperbranched polymers cross-linked via dynamic boronic ester linkages, suitable for the intravaginal therapies of vulvovaginosis. For hyperbranched polyglycidol with phenyl-enriched core, we achieved the improved solubilization of clotrimazole, a water-insoluble drug where the structure consists of four nonconjugated aromatic rings. The drug solubilization within the HbPGL-based unimolecular micelles increased gradually along with the degree of hydrophobization of the core.

Our study revealed a significant influence of HbPGL core hydrophobization with phenyl rings incorporated *via* ester or urethane linkages on the rheological properties of hydrogels constructed of core-modified HbPGL cross-linked with poly(acrylamide-*ran*-2-acrylamidephenylboronic acid). The thermal stability of hydrogels composed of hydrophobized HbPGL was higher, i.e., even above 50 °C in comparison to the neat HbPGL-based system (39.8 °C). These data input that hydrogel platforms will be stable at the temperature conditions of the vagina, and thus the degradation of the network can be triggered with glucose molecules present in the vaginal fluid. Furthermore, the elastic strength of the networks, determined based on *G*_N_ values, increased with the gradual enrichment of HbPGL core with phenyl moieties. At 40 °C, *G*_N_ was over 20 times higher for hydrogel constructed of HbPGL, where the monohydroxyl groups were hydrophobized at 55 mol % in comparison to neat HbPGL-based hydrogel. Moreover, the activation energy of the relaxation of hydrophobized macromolecules in the dynamic network was higher in comparison to that determined for the system composed of neat HbPGL macromolecules. These changes in the rheological properties of hydrogel systems can be ascribed to the intermolecular hydrophobic associations between phenyl groups, which besides boronic esters played the role of the additional cross-links in the network. The increasing contribution of hydrophobic interactions in the network resulted in the gradual reduction of the flow behavior and self-healing ability of the network as an effect of the change of the cross-linking mechanism based on the hydrophobic interactions from a single-sticker to aggregate-type model. These results input that to obtain the hydrogel of the efficient drug loading and beneficial rheological behavior such as injectability and self-healing properties, the HbPGL of a proper degree of hydrophobization has to be applied.

In the face of numerous side effects of orally administered drugs, especially in the case of women suffering from gastrointestinal tract disorders, there is a necessity to develop formulations of hydrophobic drugs for gynecology. Unimolecular micelle-based hydrogel platforms presented here can be used as carriers of various aromatic-equipped drugs in the treatment of infections evoked by other microorganisms (bacteria, protozoan, i.e., *Trichomonas vaginalis*) or in anticancer therapies. Each system, however, requires optimization such as the choice of proper groups adjusted to the chemical nature of an encapsulated drug, the degree of hydrophobization along with the rheological properties of a hydrogel platform to attain the therapeutic effect.

## Supporting Information Available

Administration of hydrogel systems with a syringe (Video S1) (MP4)
Administration of hydrogel systems with a syringe (Video S2) (MP4)
Administration of hydrogel systems with a syringe (Video S3) (MP4)
^1^H, ^13^C, and ^1^H DOSY NMR spectra of hydrophobized HbPGLs, GPC spectrum of acrylamide-2-acrylamidephenylboronic acid copolymer, cytotoxicity tests on HMEC and HeLa lines after 24 h, and rheology characteristics of hydrogels (PDF)
