# Supplementary material for: Self-Healable, Injectable Hydrogel with Enhanced Clotrimazole Solubilization as a Potential Therapeutic Platform for Gynecology
Source: Biomacromolecules. 2022 Sep 8;23(10):4203–19. doi: 10.1021/acs.biomac.2c00691 (PMC9554913; doi:10.1021/acs.biomac.2c00691)
Supplement: Supplementary file 4 — bm2c00691_si_004.pdf [file bm2c00691_si_004.pdf]

Self-healable, injectable hydrogel with enhanced clotrimazole solubilization as a potential therapeutic platform for gynecology

Monika Gosecka*†, Daria Jaworska-Krych†, Mateusz Gosecki†, Ewelina Wielgus†, Monika Marcinkowska‡, Anna Janaszewska‡, Barbara Klajnert-Maculewicz‡

†Centre of Molecular and Macromolecular Studies, Polish Academy of Sciences

Sienkiewicza 112, 90-363 Lodz, Poland

‡Department of General Biophysics, Faculty of Biology and Environmental Protection, University of Lodz, 141/143 Pomorska Street, 90-236 Lodz, Poland

** Correspondence: mdybko@cbmm.lodz.pl*

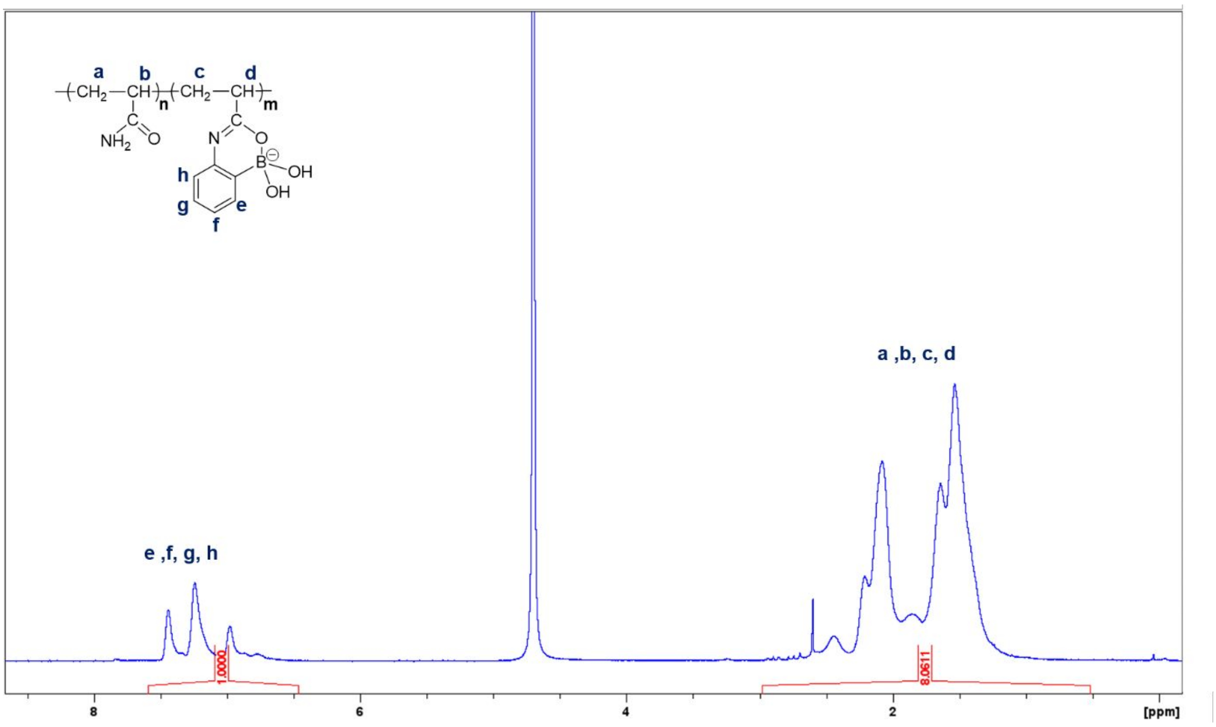

Figure S1. ^{1}H NMR spectrum of poly(AM-*ran*-2-AAPBA), M_n=43000 recorded in D_2O.

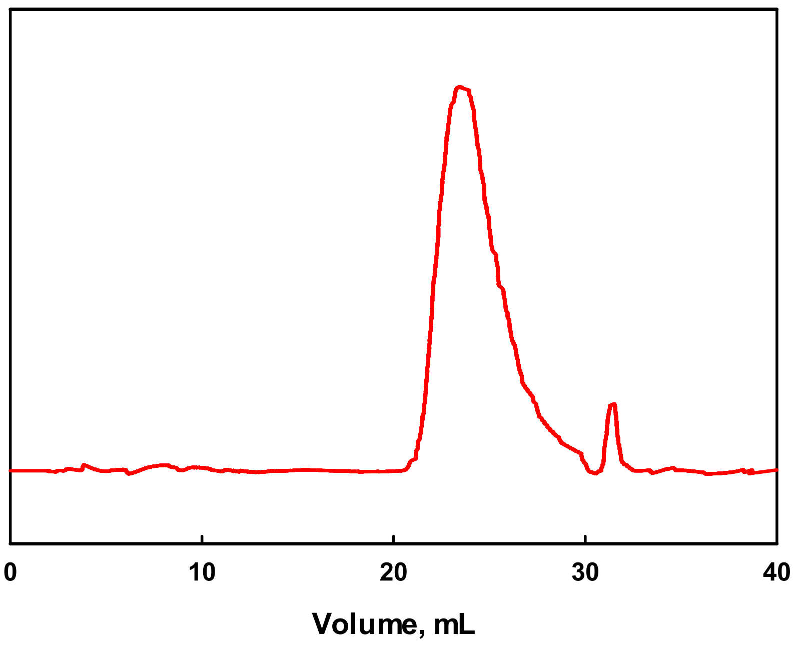

Figure S2. GPC chromatogram of poly(AM-*ran*-2-AAPBA) performed in an aqueous solution of NaN3 (0.1 wt %) at a flow rate of 1.0 mL min using a chromatograph (Knauer K-501 HPLC pump) equipped with a degasser (4-Channel Degasser; K-5004, Knauer), three TSK-GEL columns: G5000 PW XL 1 3000 PW XL 1 2500 PW XL (7.8 3 300 mm; Tosho; 26 8C), an LDC RI detector.

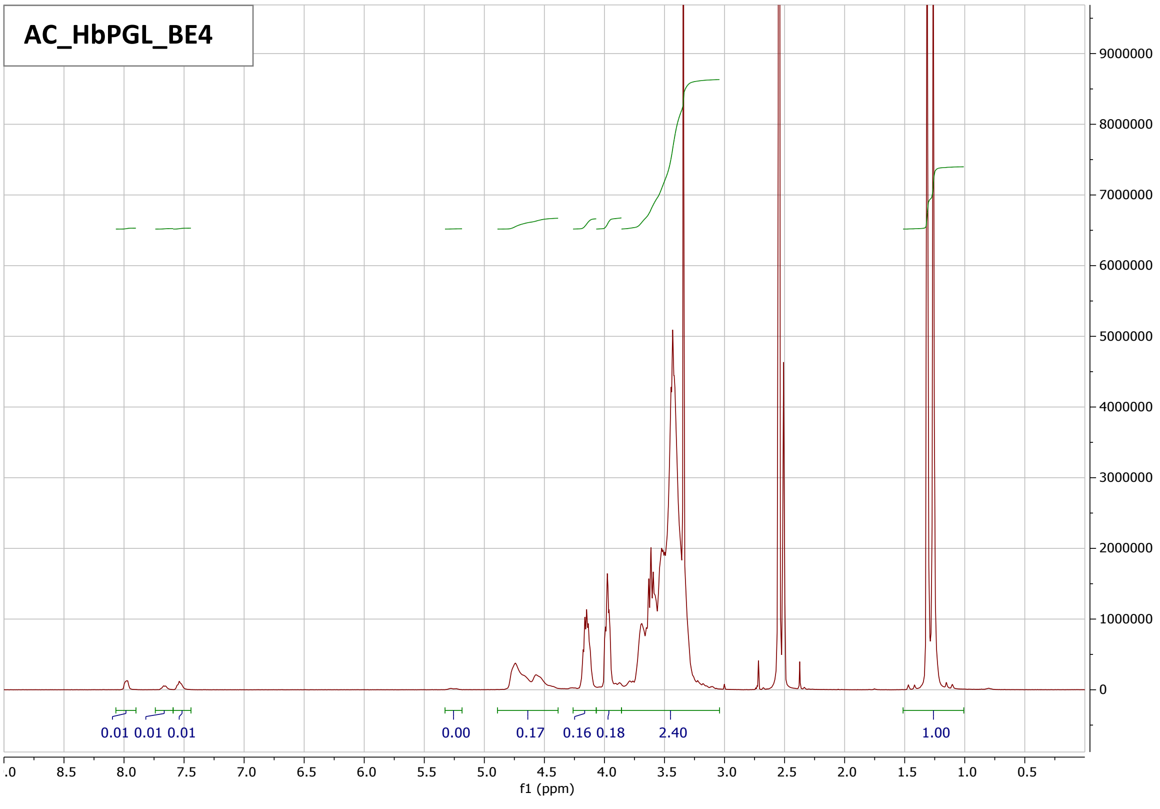

Figure S3. ^{1}H NMR spectrum of AC_HbPGL_BE4 recorded in DMSO-d_6.

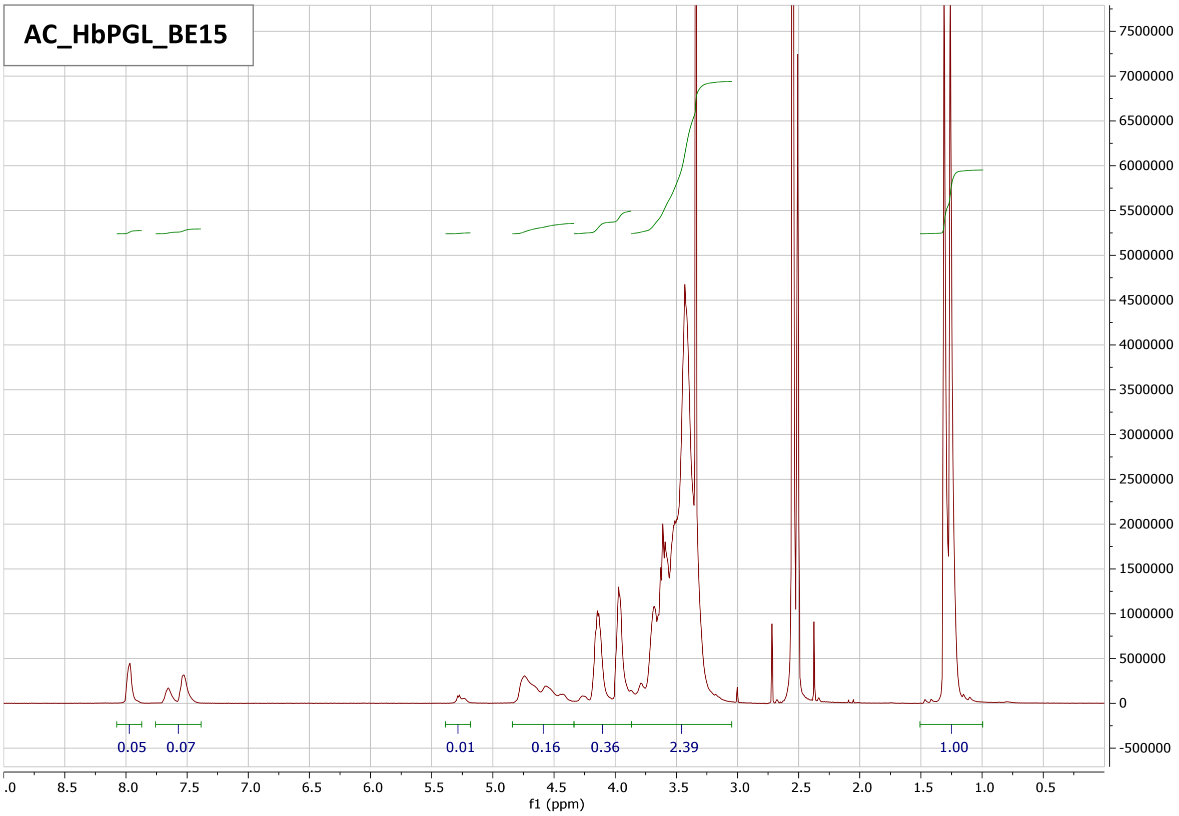

Figure S4. ^{1}H NMR spectrum of AC_HbPGL_BE15 recorded in DMSO-d_6.

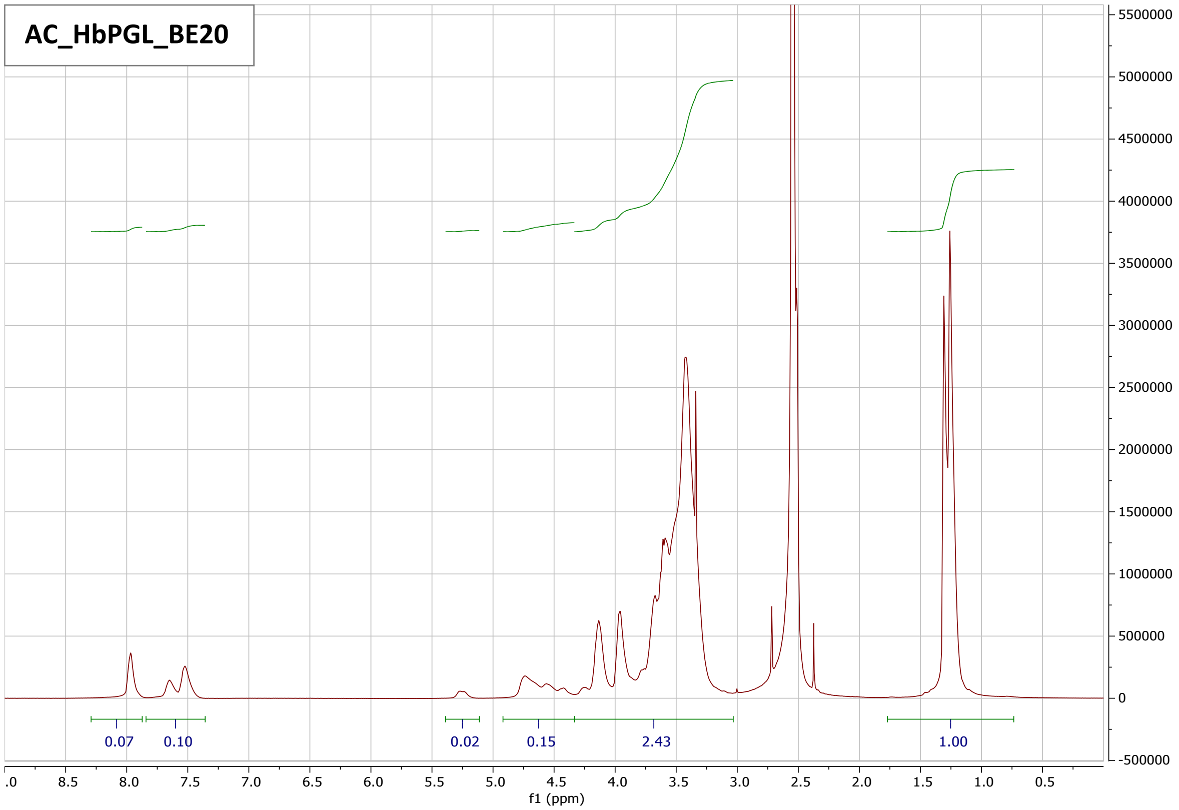

Figure S5. ^{1}H NMR spectrum of AC_HbPGL_BE20 recorded in DMSO-d_6.

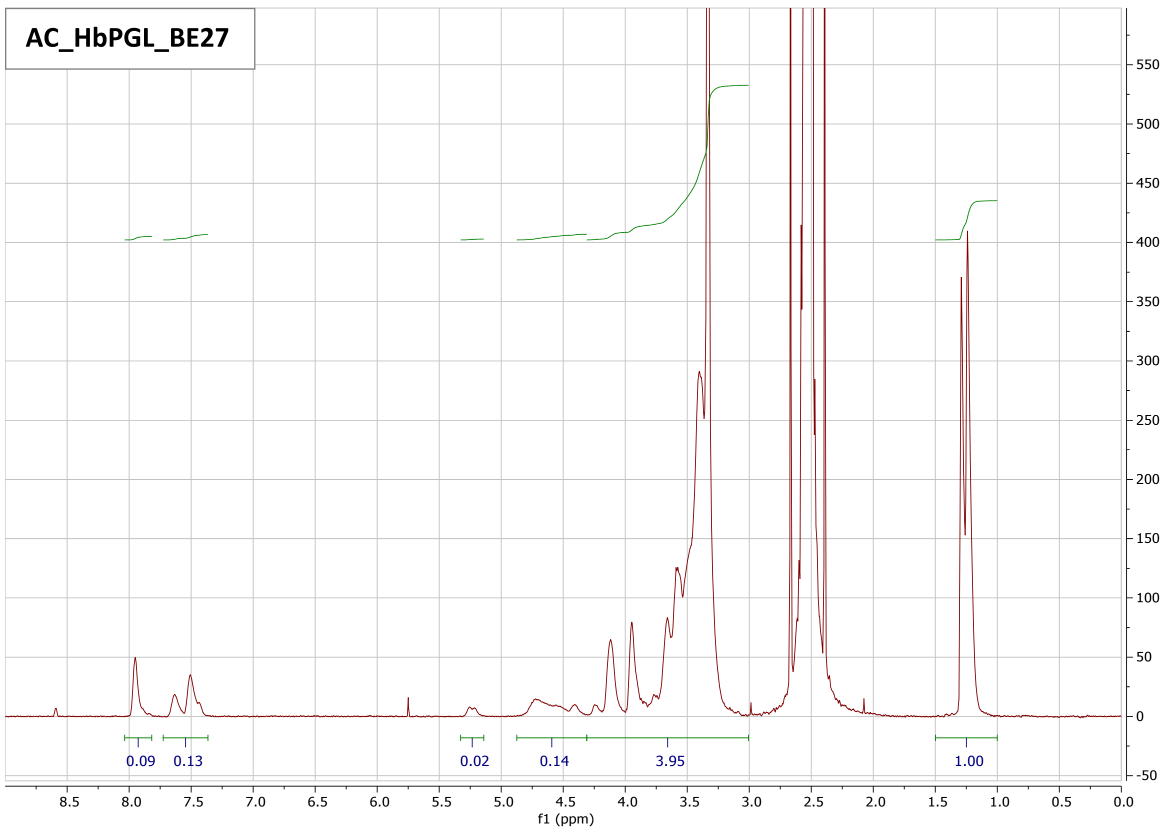

Figure S6. ^{1}H NMR spectrum of AC_HbPGL_BE27 recorded in DMSO-d_6.

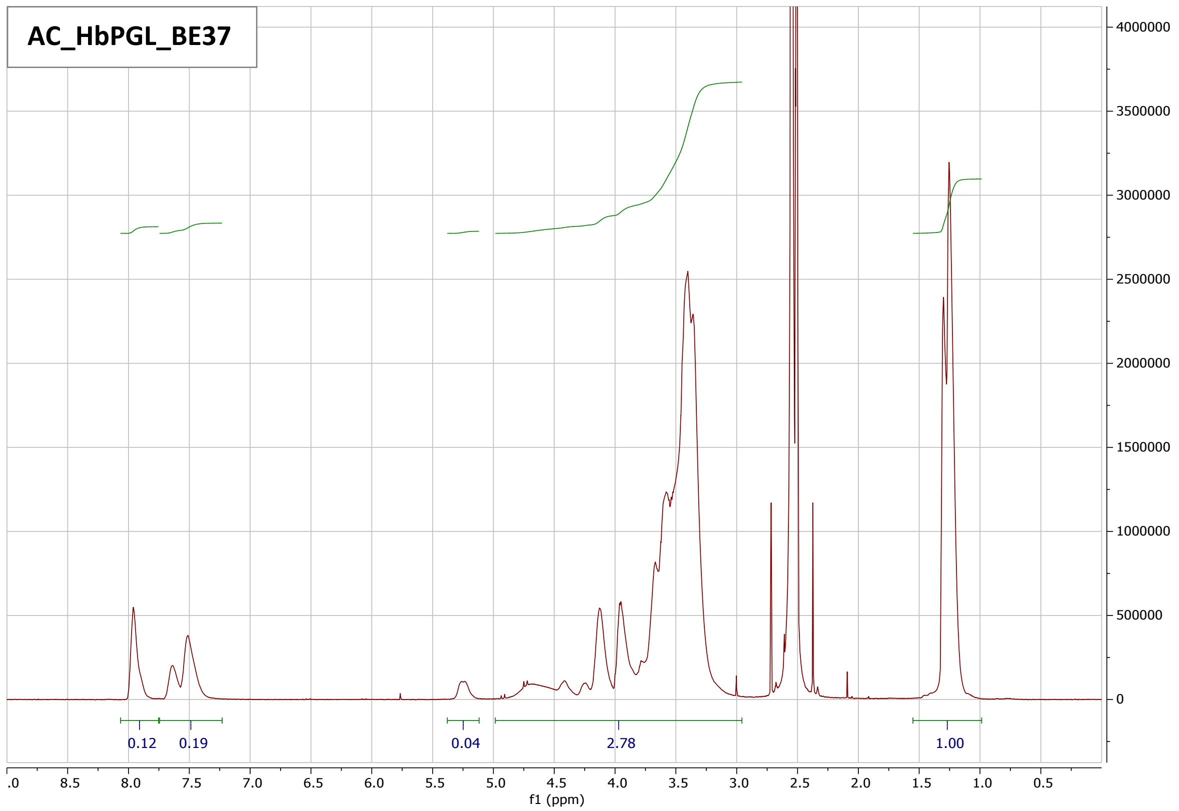

Figure S7. ^{1}H NMR spectrum of AC_HbPGL_BE37 recorded in DMSO-d_6.

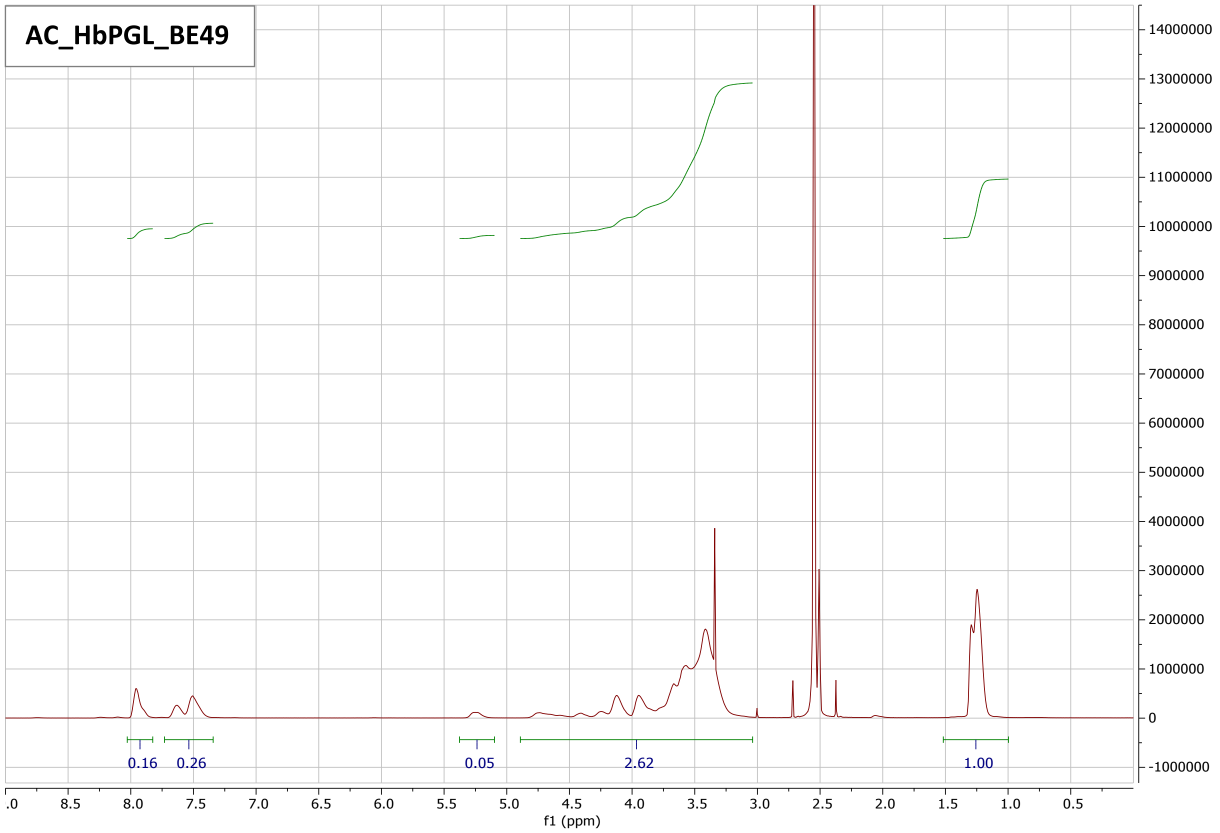

Figure S8. ^{1}H NMR spectrum of AC_HbPGL_BE49 recorded in DMSO-d$_6$.

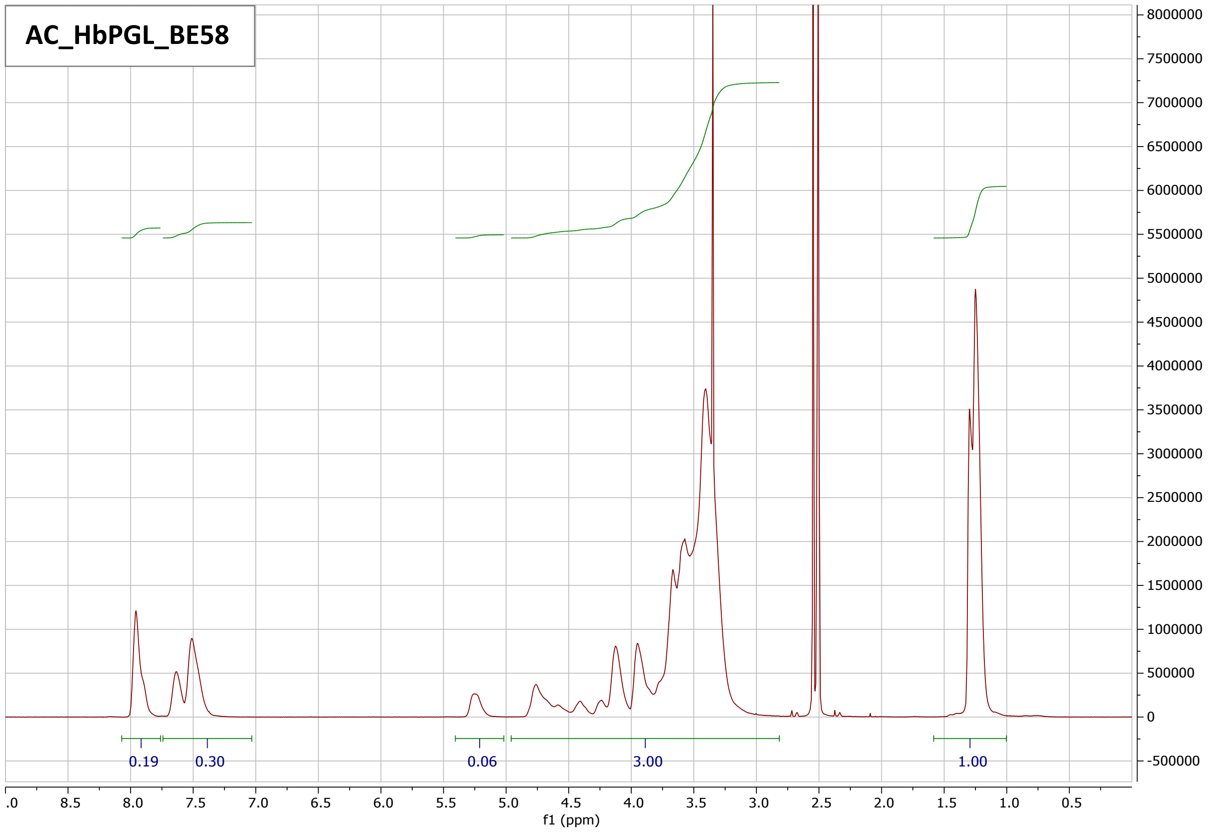

Figure S9. ^{1}H NMR spectrum of AC_HbPGL_BE58 recorded in DMSO-d$_6$.

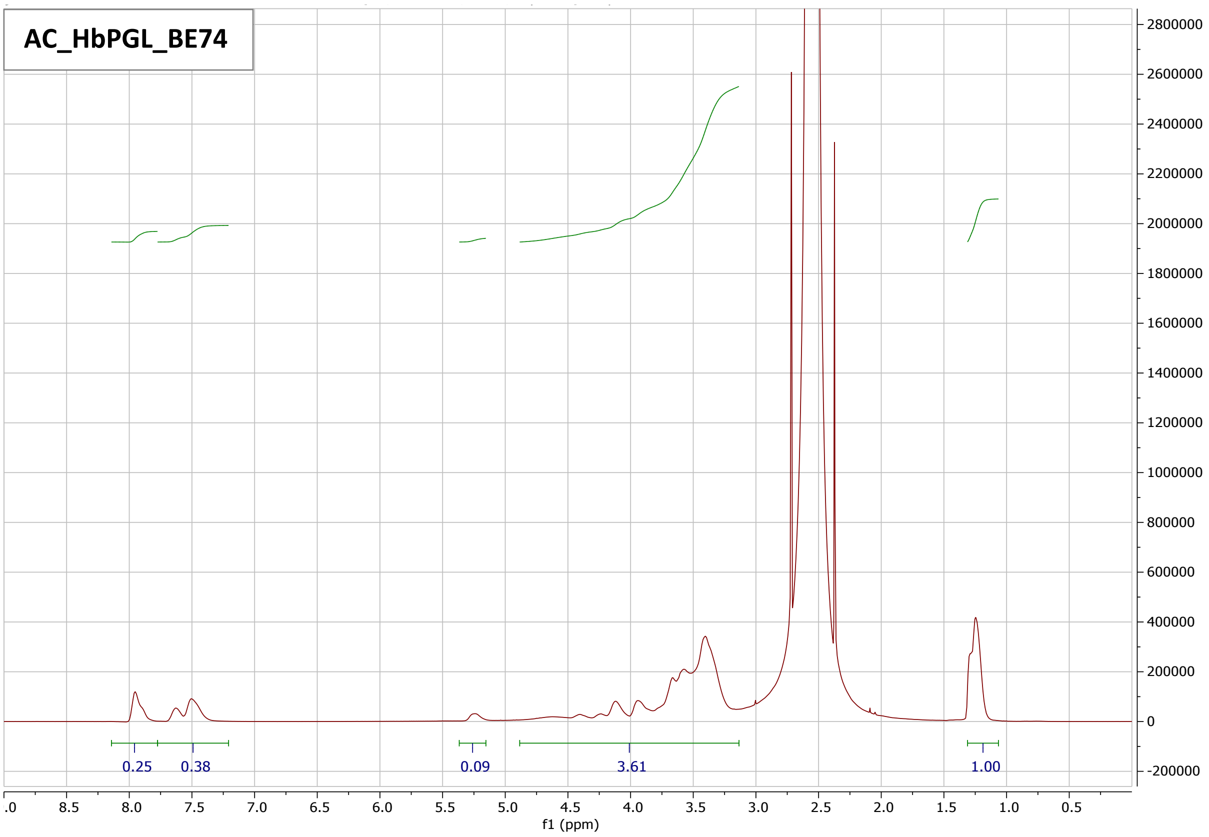

Figure S10. ^{1}H NMR spectrum of AC_HbPGL_BE74 recorded in DMSO-d_6.

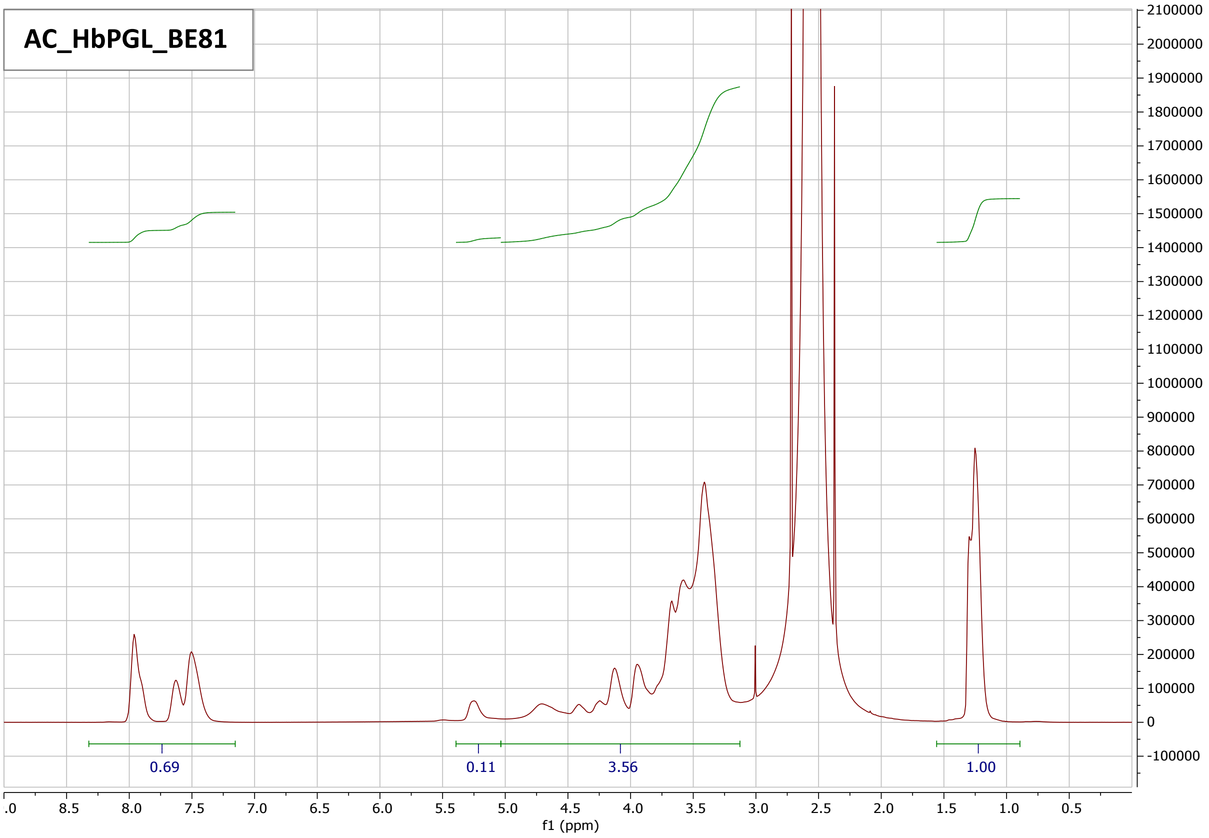

Figure S11. ^{1}H NMR spectrum of AC_HbPGL_BE81 recorded in DMSO-d_6.

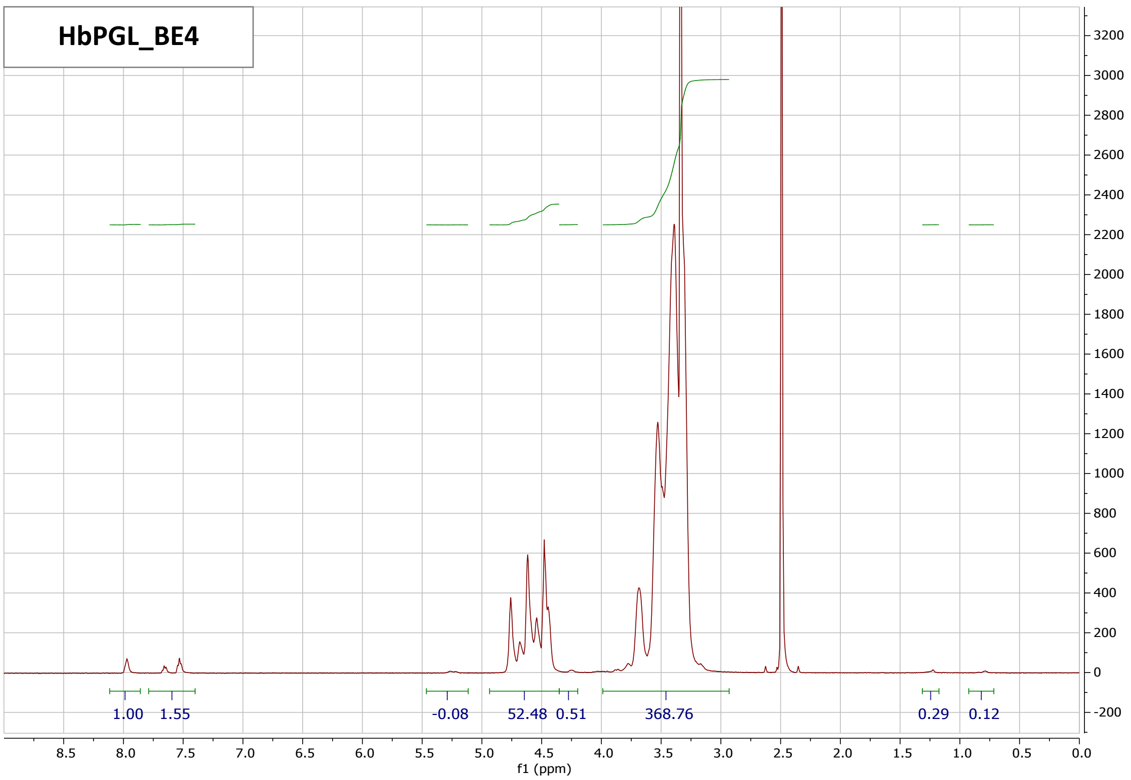

Figure S12. ^{1}H NMR spectrum of HbPGL_BE4 recorded in DMSO-d_6.

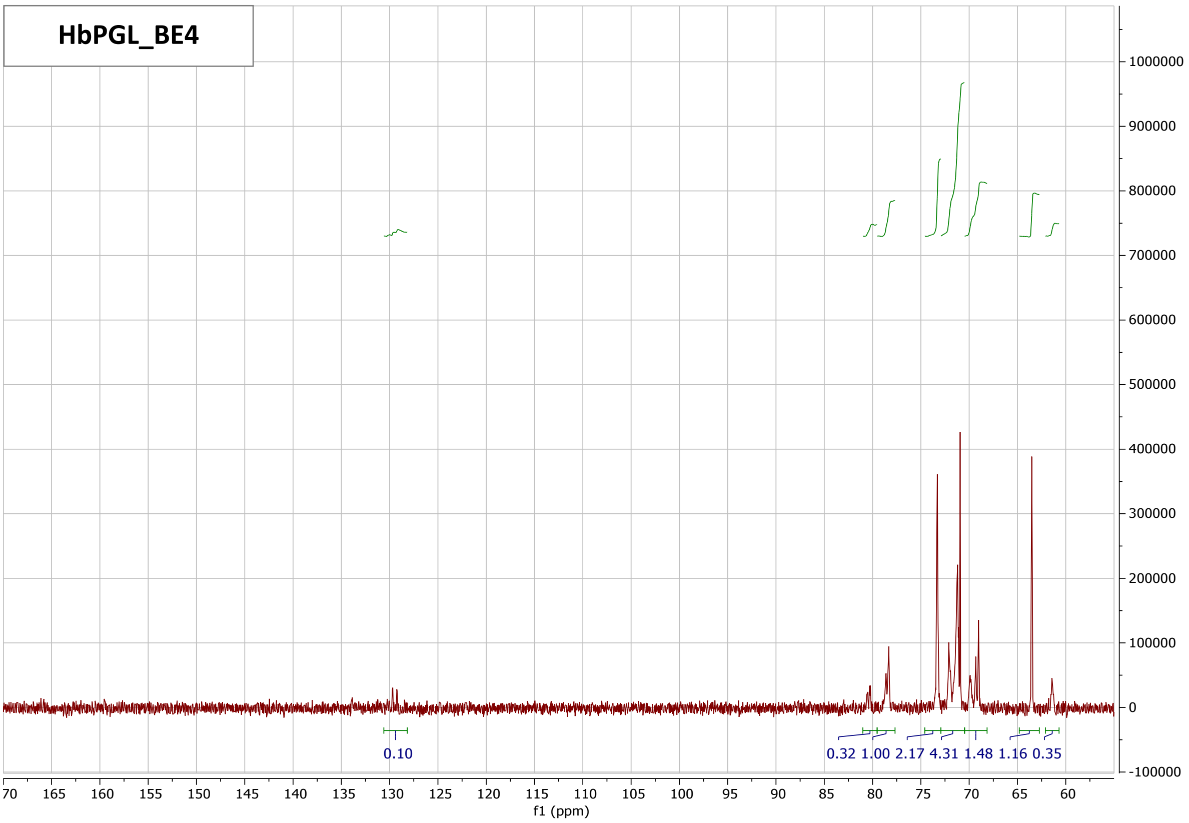

Figure S13. ^{13}C INVGATED NMR spectrum of HbPGL_BE4 recorded in DMSO-d_6.

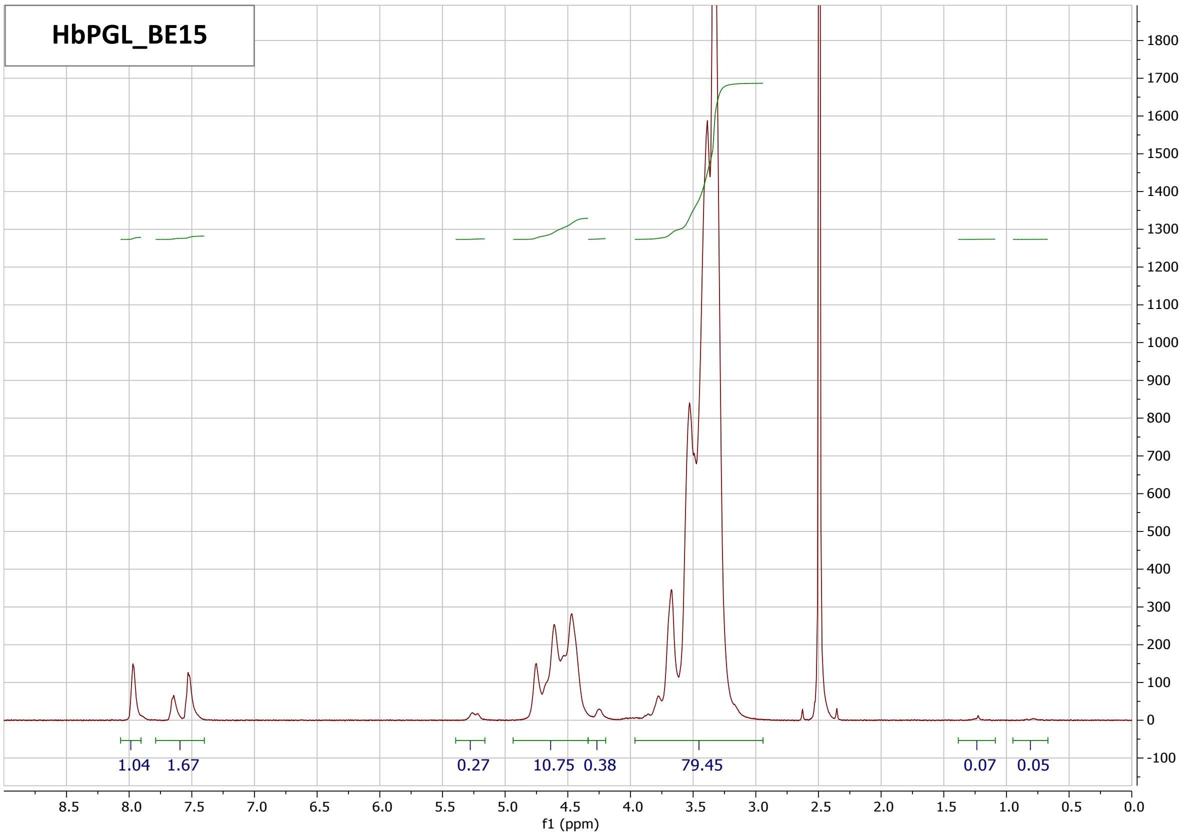

Figure S14. ^{1}H NMR spectrum of HbPGL_BE15 recorded in DMSO-d$_6$.

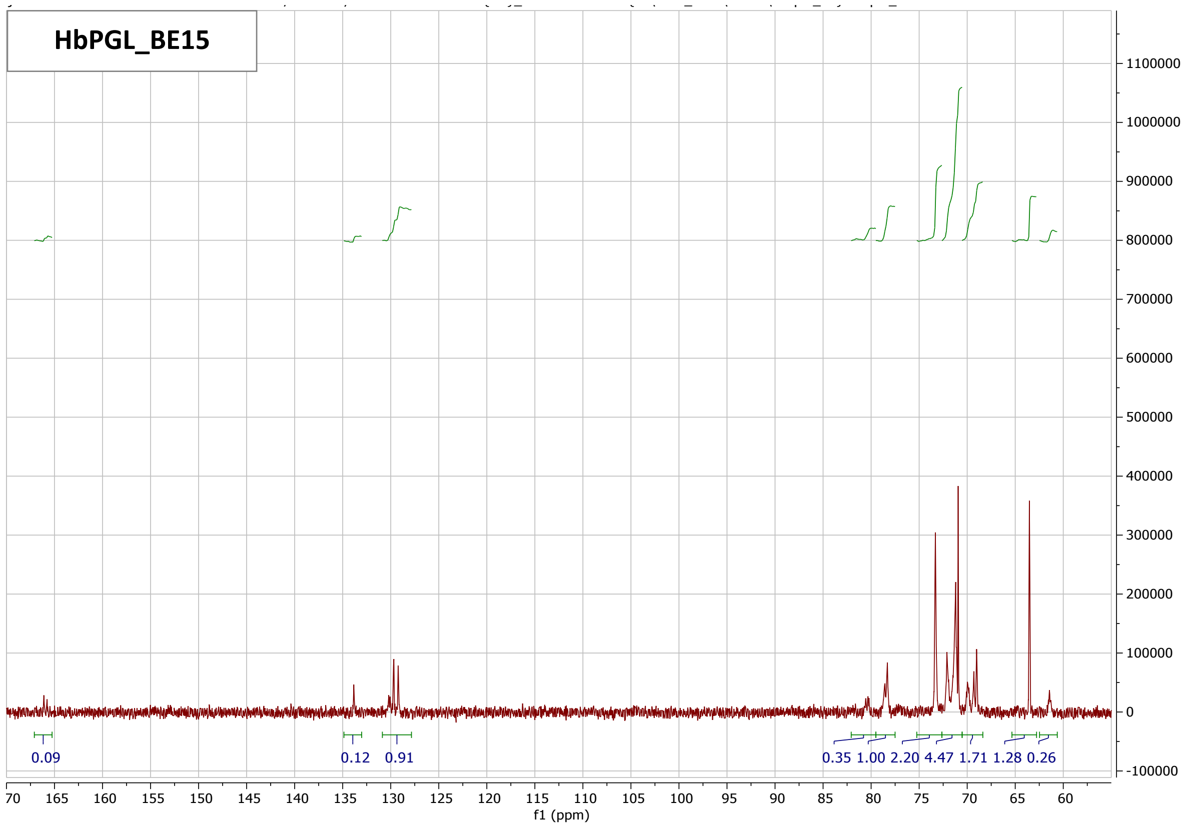

Figure S15. ^{13}C INVGATED NMR spectrum of HbPGL_BE15 recorded in DMSO-d$_6$.

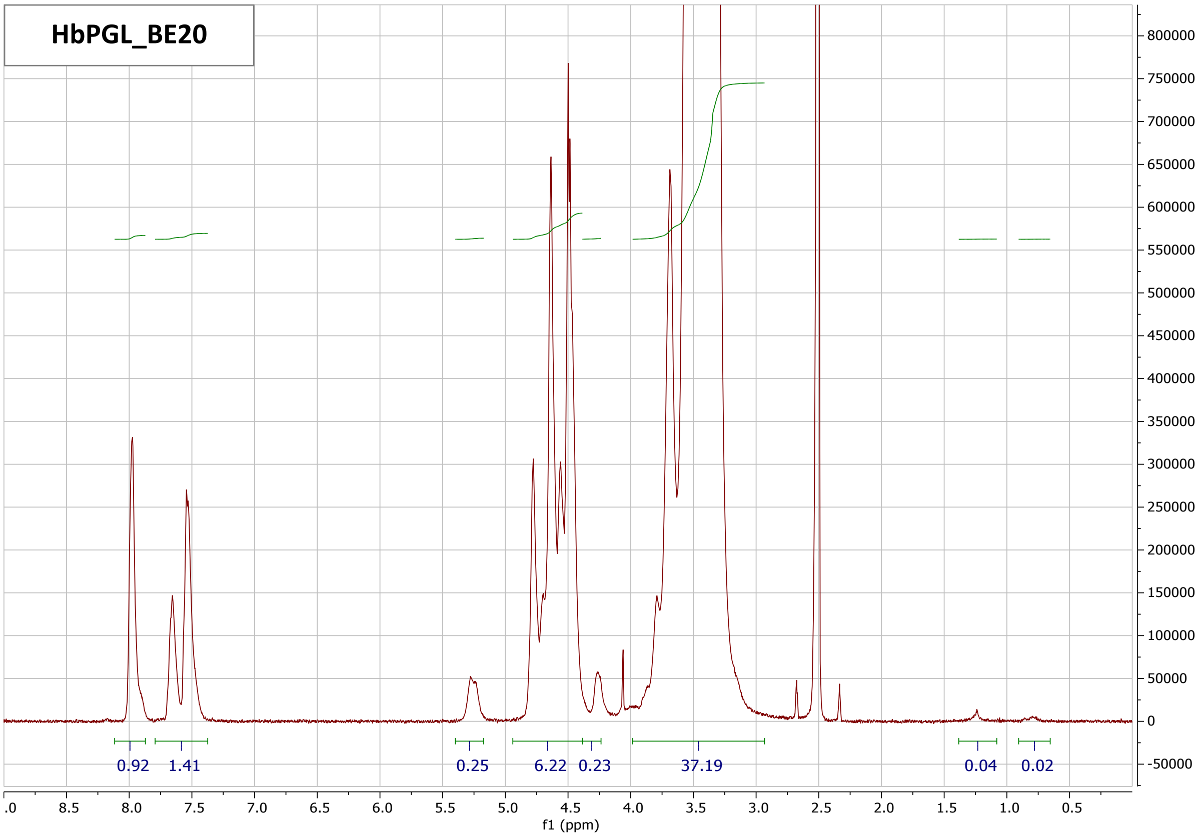

Figure S16. ^{1}H NMR spectrum of HbPGL_BE20 recorded in DMSO-d$_{6}$.

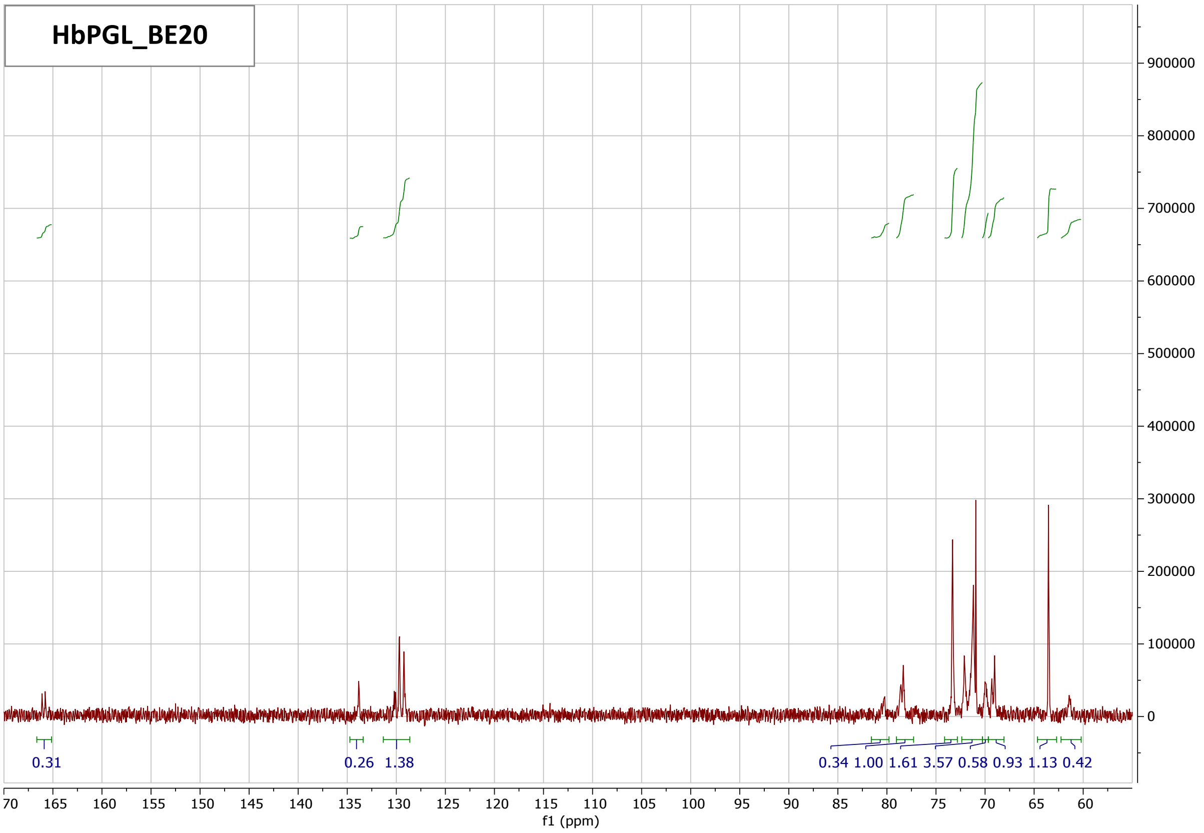

Figure S17. ^{13}C INVGATED NMR spectrum of HbPGL_BE20 recorded in DMSO-d$_{6}$.

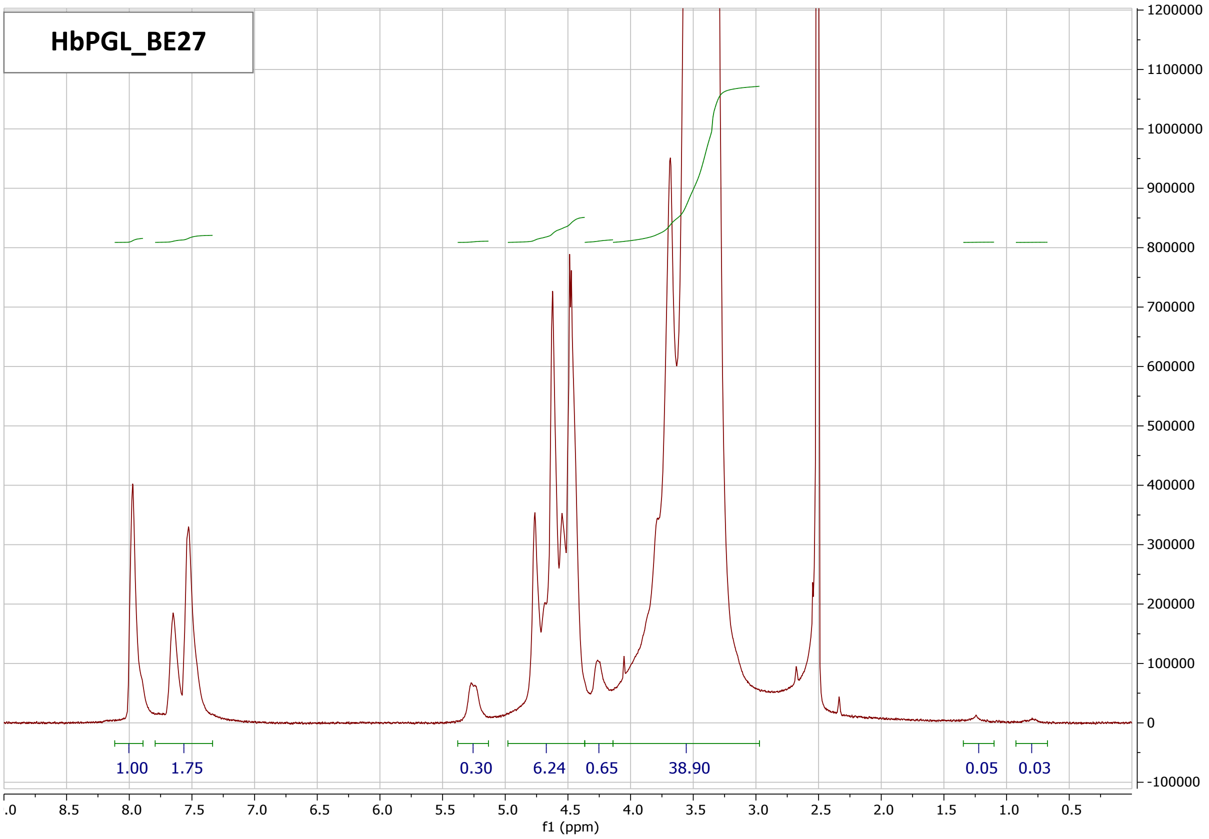

Figure S18. ^{1}H NMR spectrum of HbPGL_BE27 recorded in DMSO-d_6.

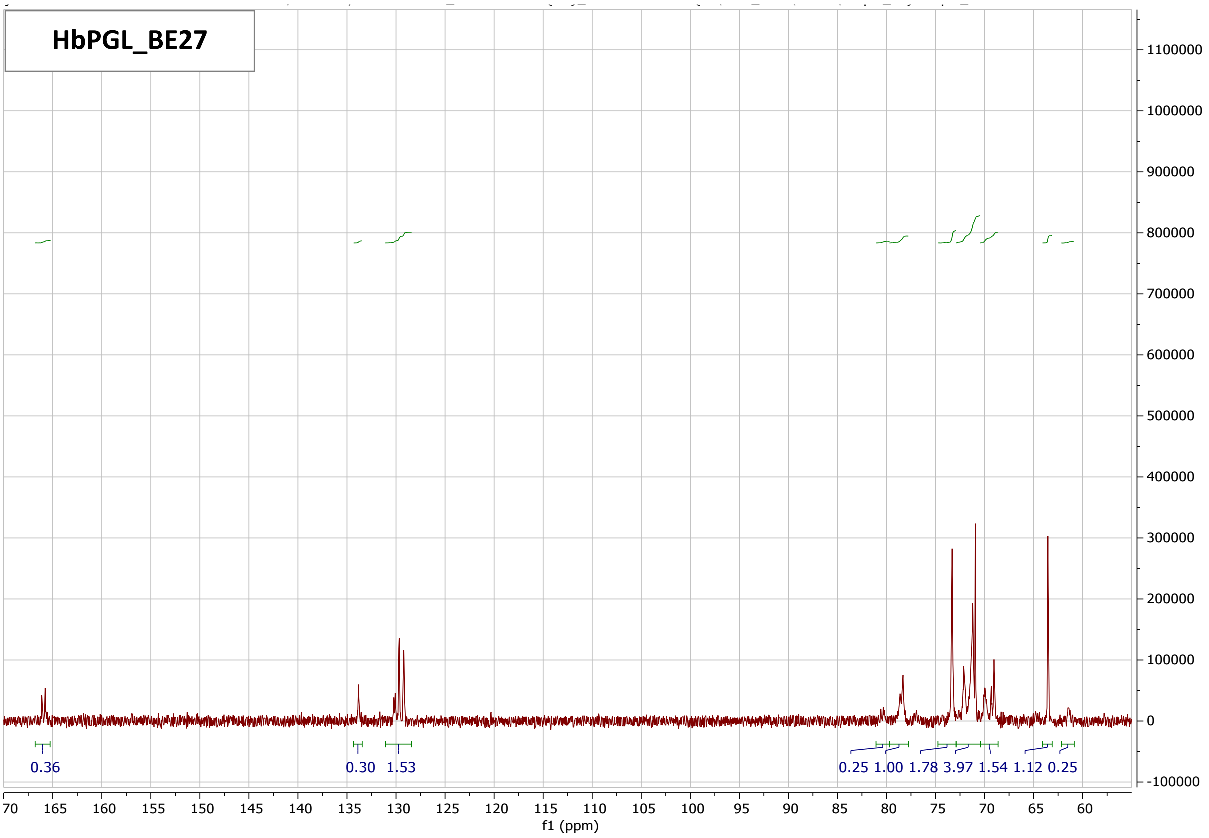

Figure S19. ^{13}C INVGATED NMR spectrum of HbPGL_BE27 recorded in DMSO-d_6.

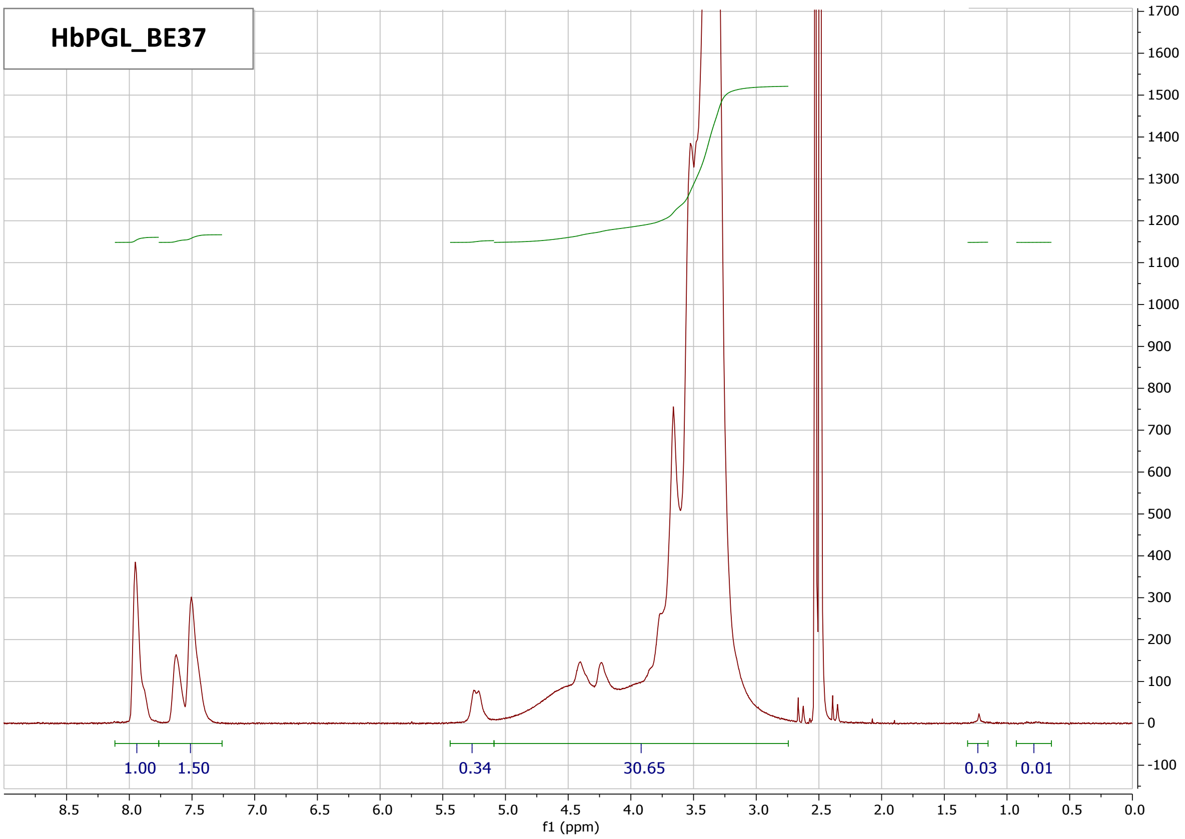

Figure S20. ^{1}H NMR spectrum of HbPGL_BE37 recorded in DMSO-d$_6$.

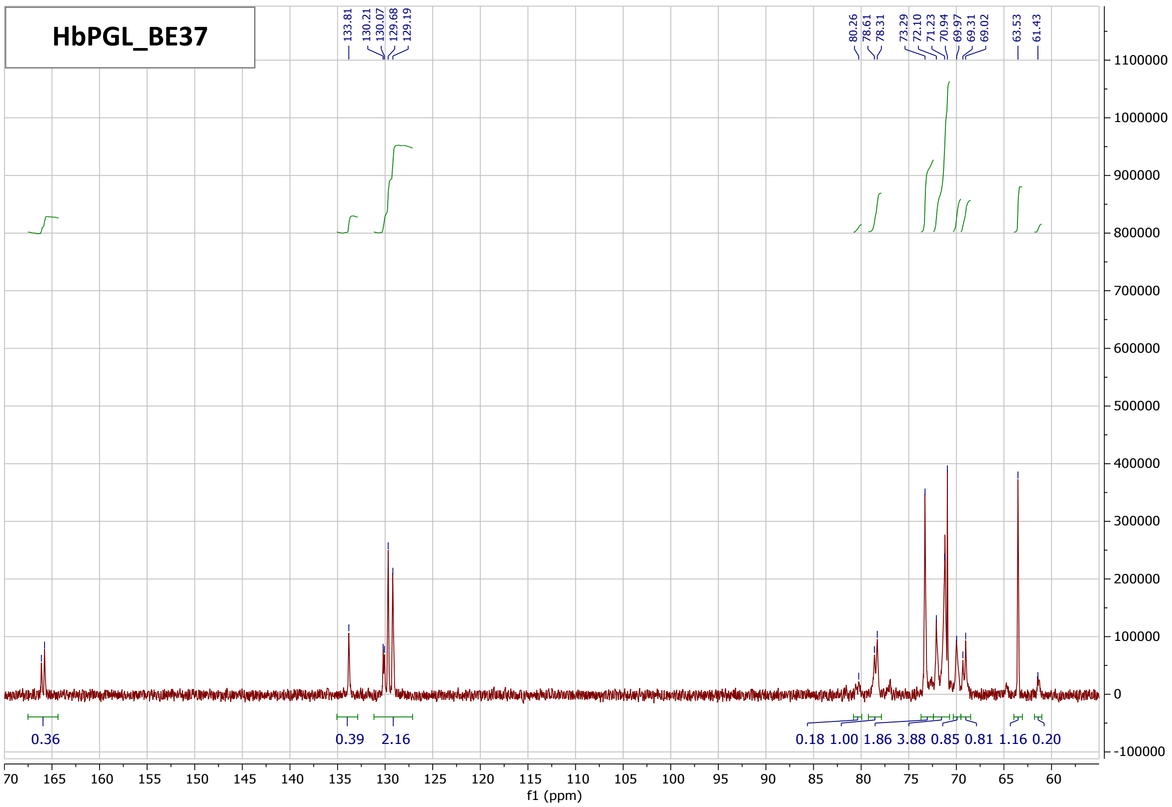

Figure S21. ^{13}C INVGATED NMR spectrum of HbPGL_BE37 recorded in DMSO-d$_6$.

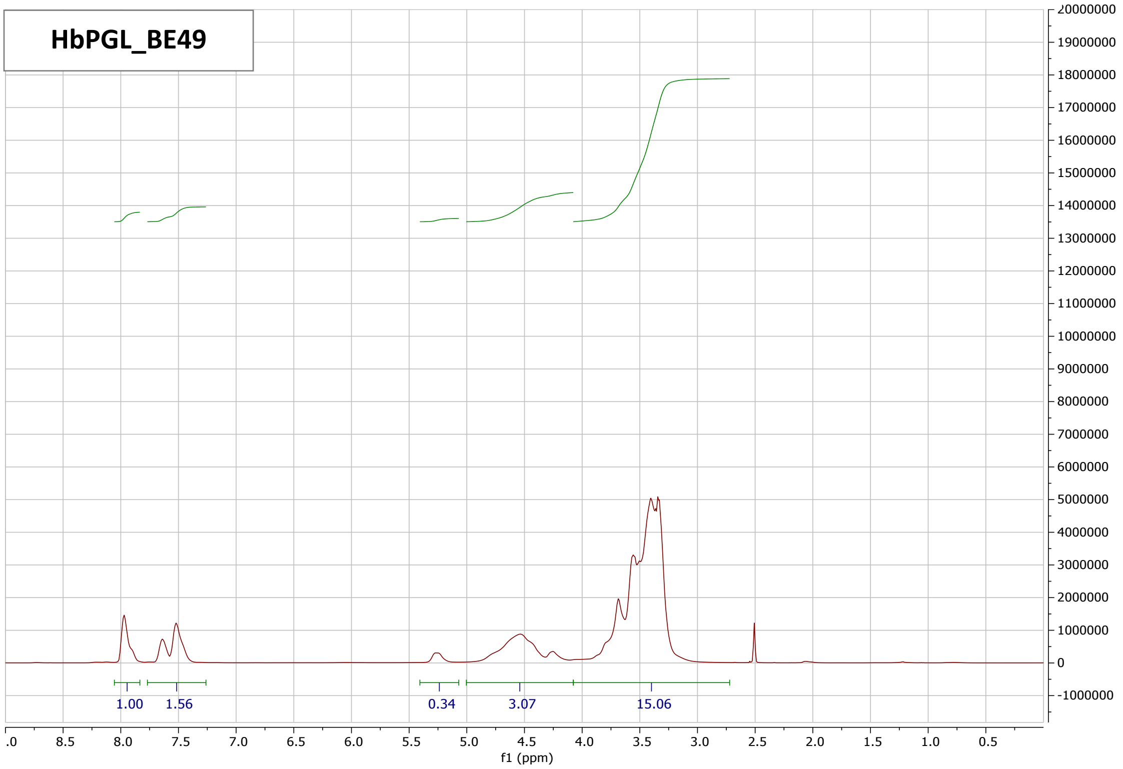

Figure S22. ^{1}H NMR spectrum of HbPGL_BE49 recorded in DMSO-d$_6$.

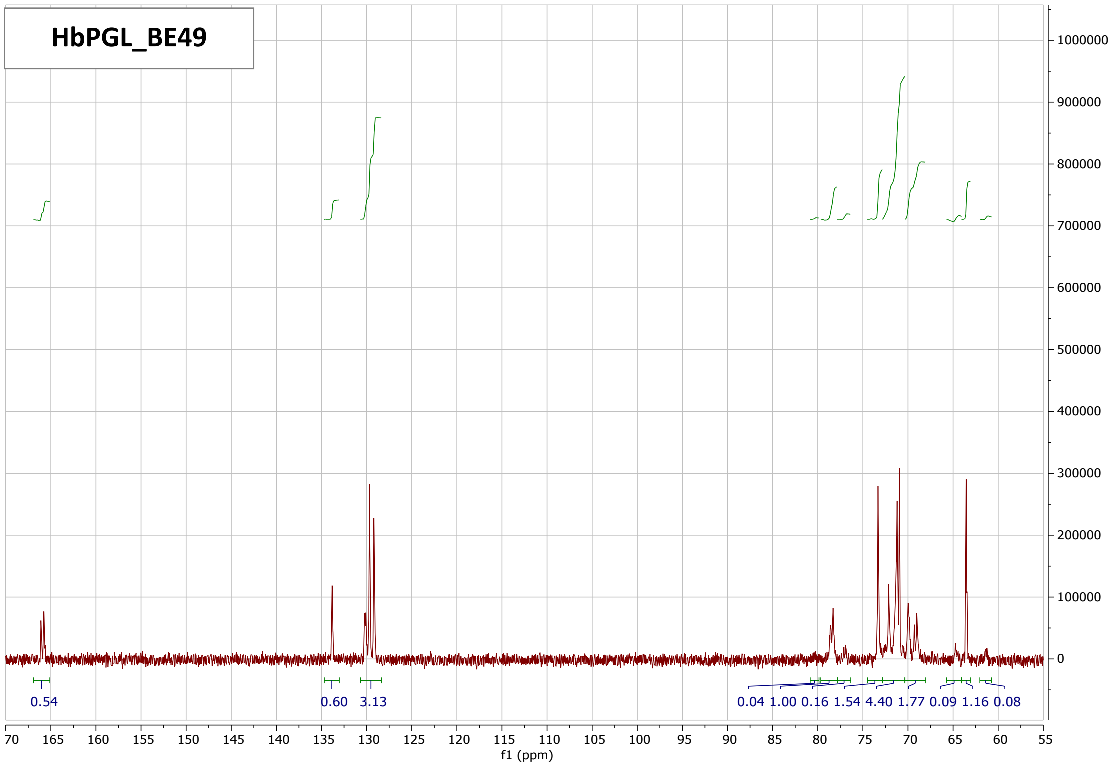

Figure S23. ^{13}C INVGATED NMR spectrum of HbPGL_BE49 recorded in DMSO-d$_6$.

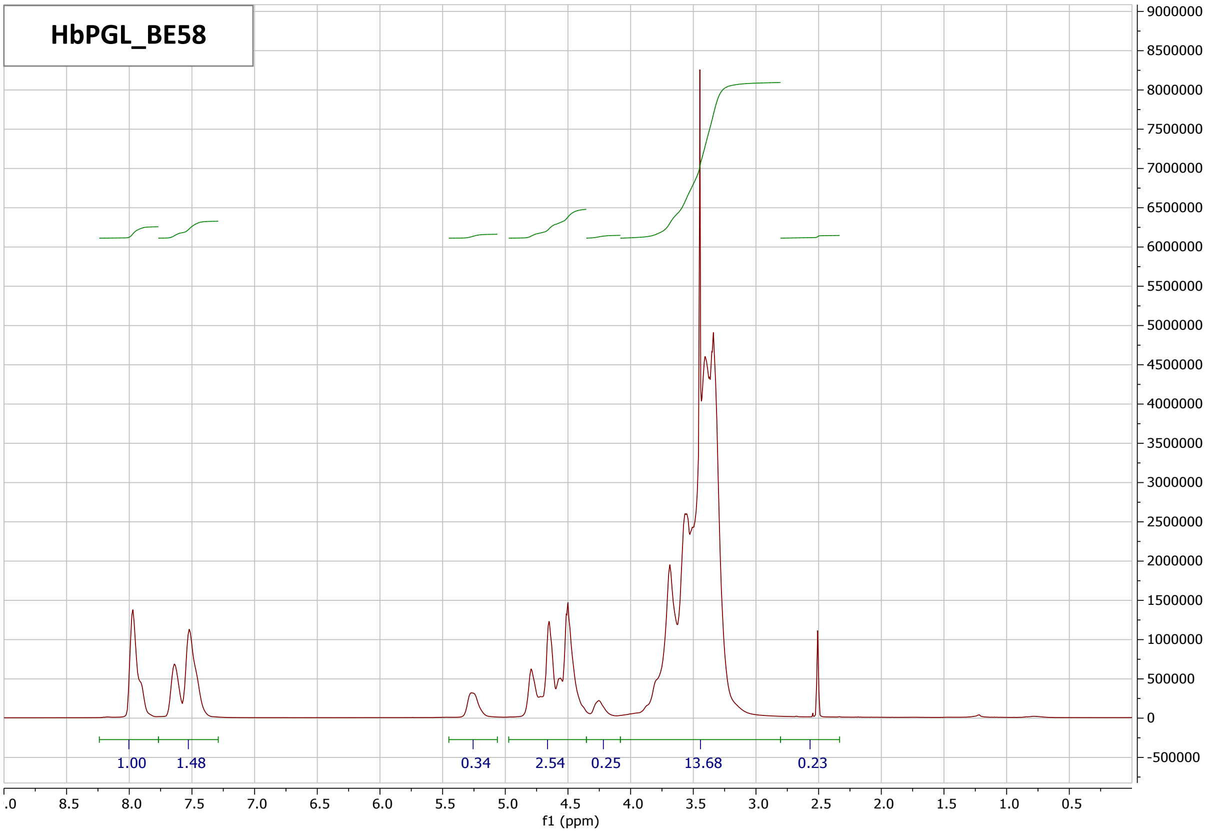

Figure S24. ^{1}H NMR spectrum of HbPGL_BE58 recorded in DMSO-d_6.

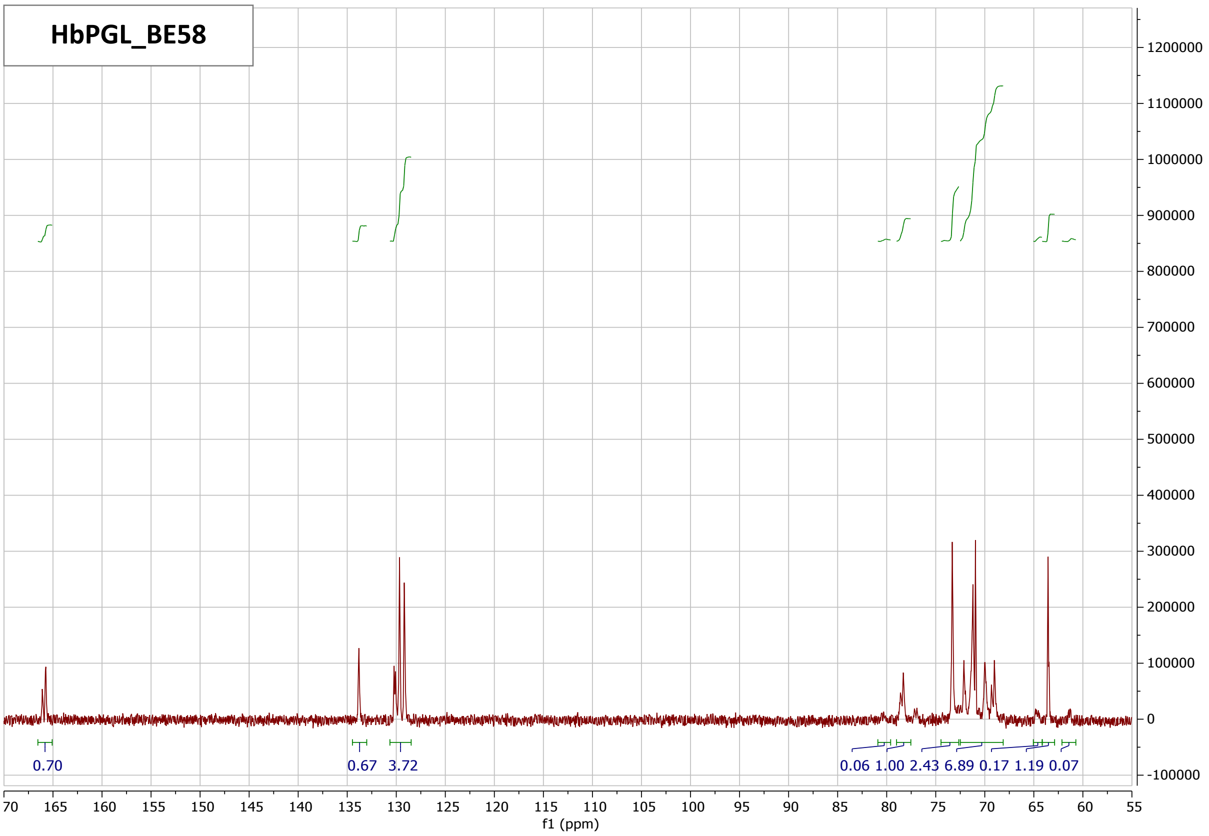

Figure S25. ^{13}C INVGATED NMR spectrum of HbPGL_BE58 recorded in DMSO-d_6.

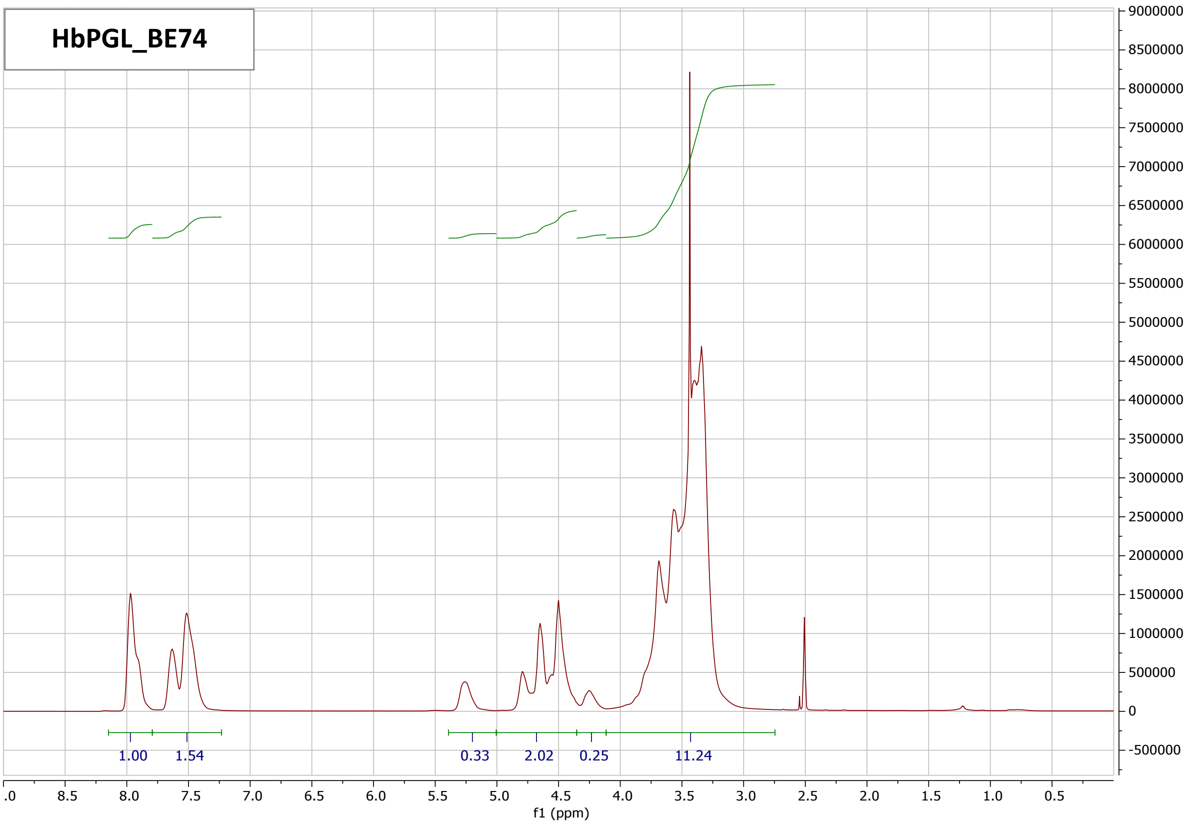

Figure S26. ^{1}H NMR spectrum of HbPGL_BE74 recorded in DMSO-d_6.

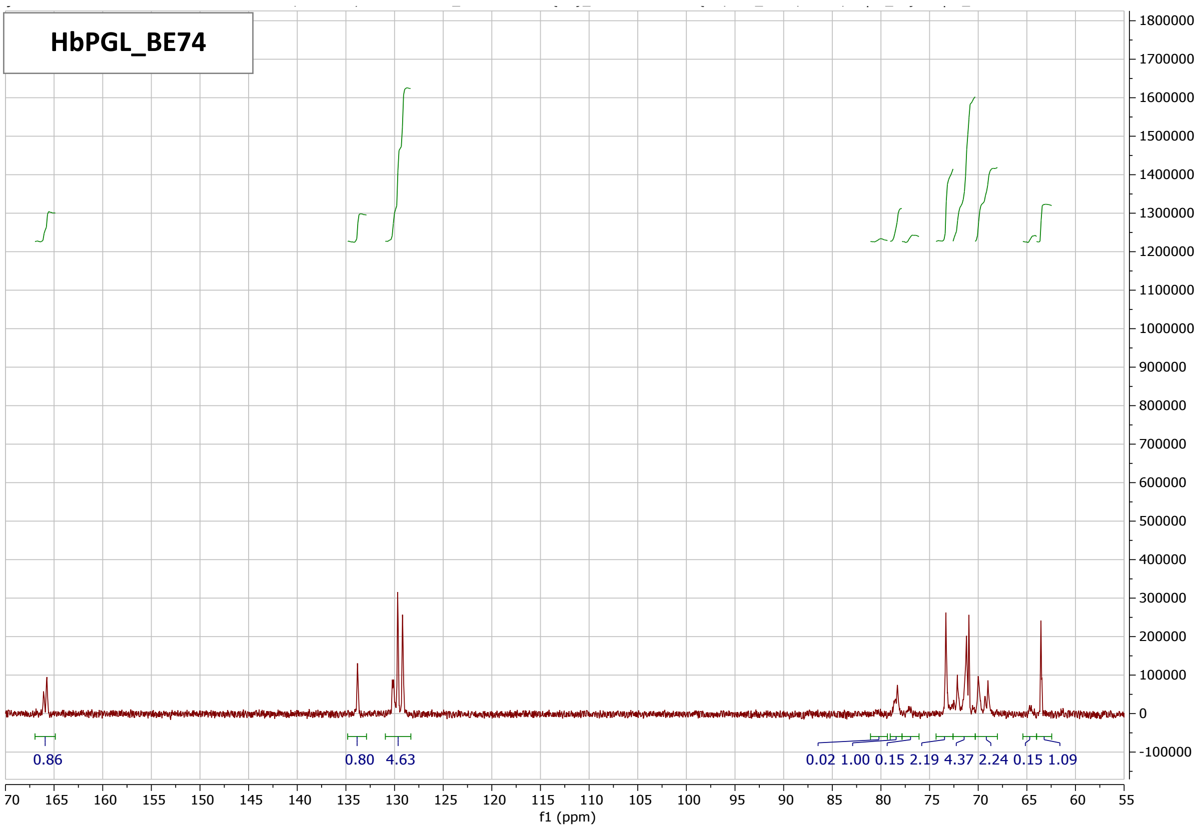

Figure S27. ^{13}C INVGATED NMR spectrum of HbPGL_BE74 recorded in DMSO-d_6.

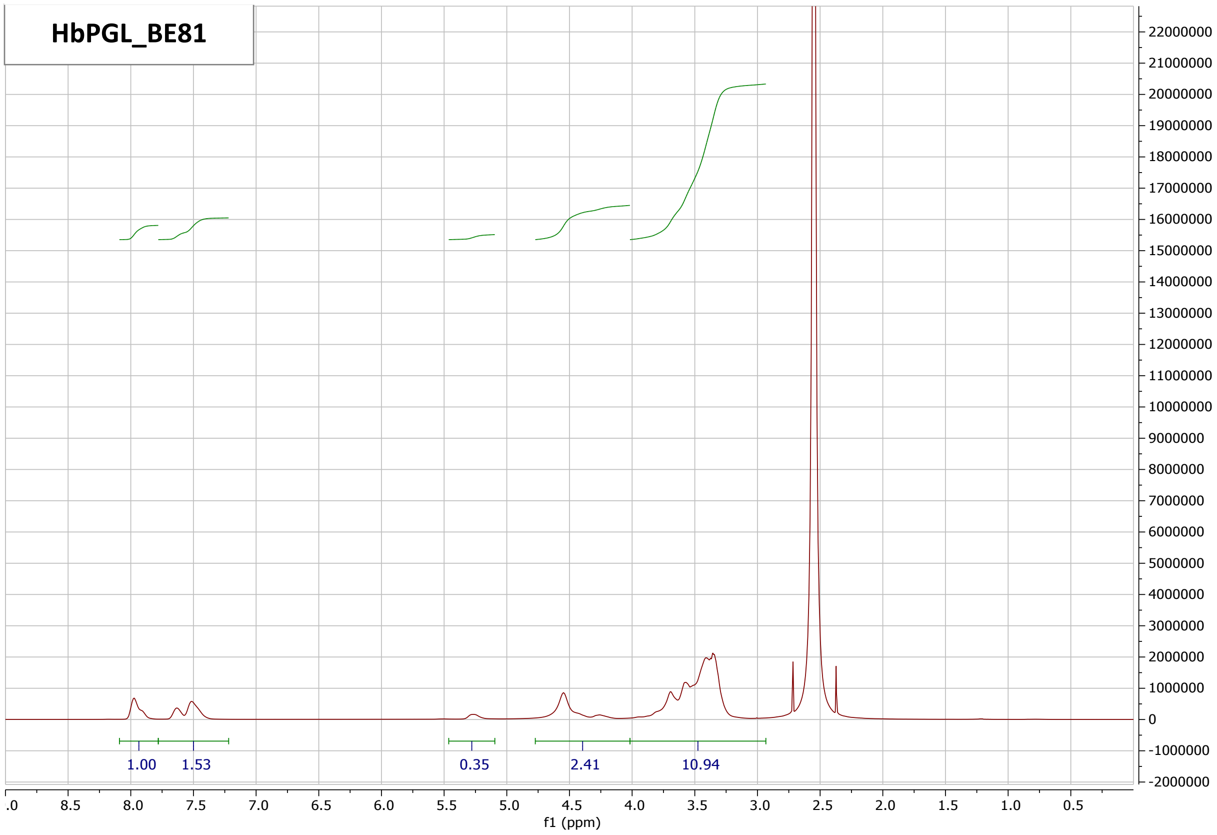

Figure S28. ^{1}H NMR spectrum of HbPGL_BE81 recorded in DMSO-d$_6$.

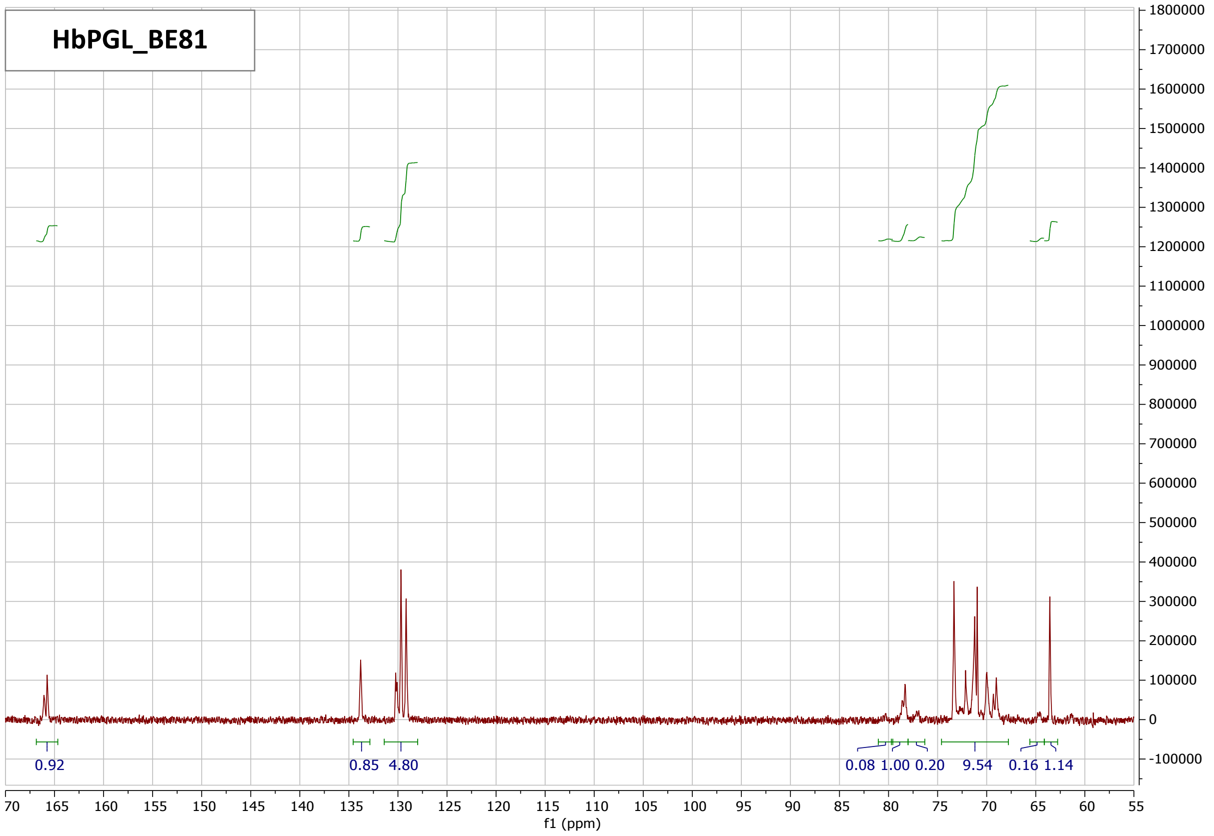

Figure S29. ^{13}C INVGATED NMR spectrum of HbPGL_BE81 recorded in DMSO-d$_6$.

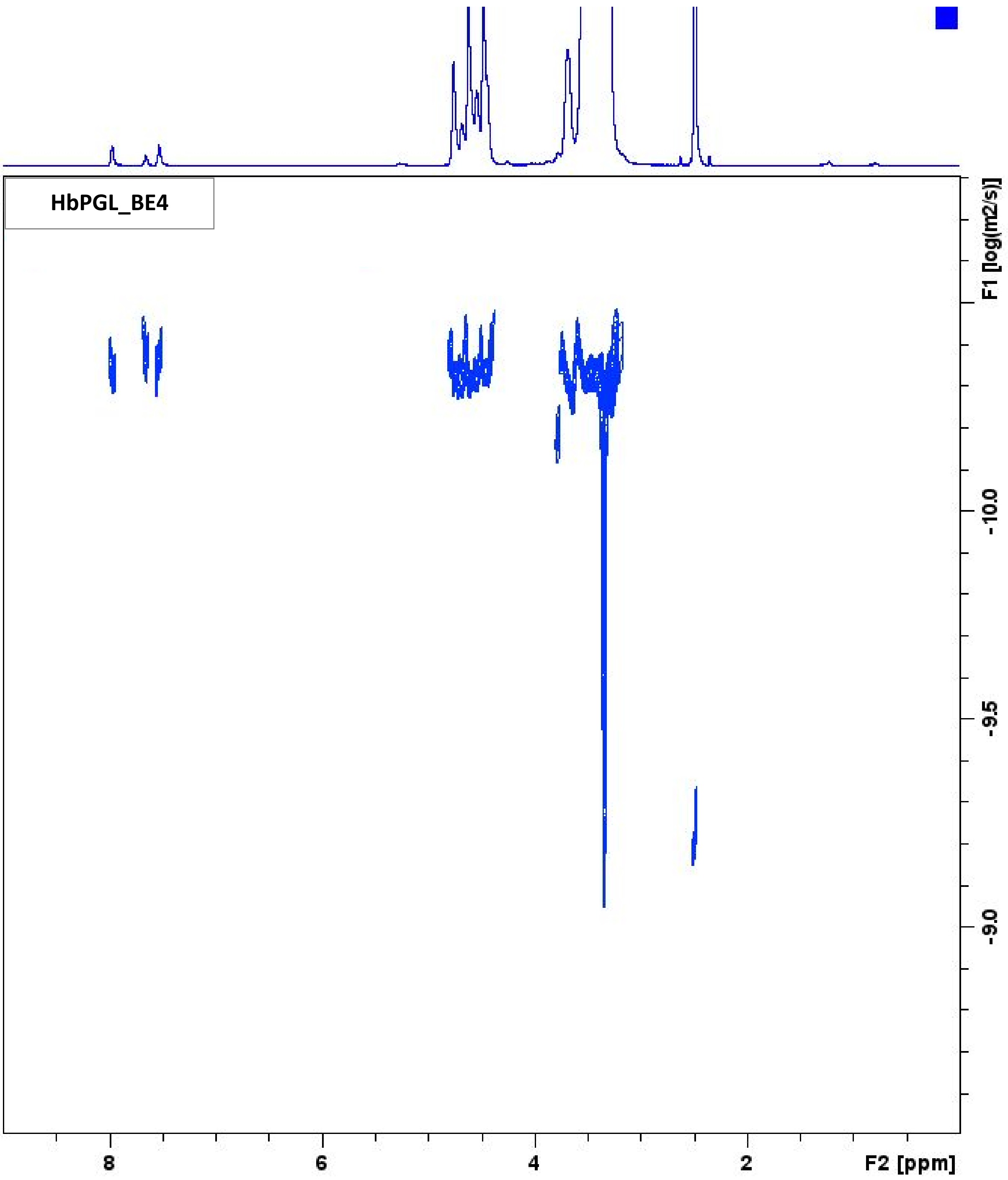

Figure S30. ^{1}H DOSY NMR spectrum of HbPGL_BE4 recorded in DMSO-d_6.

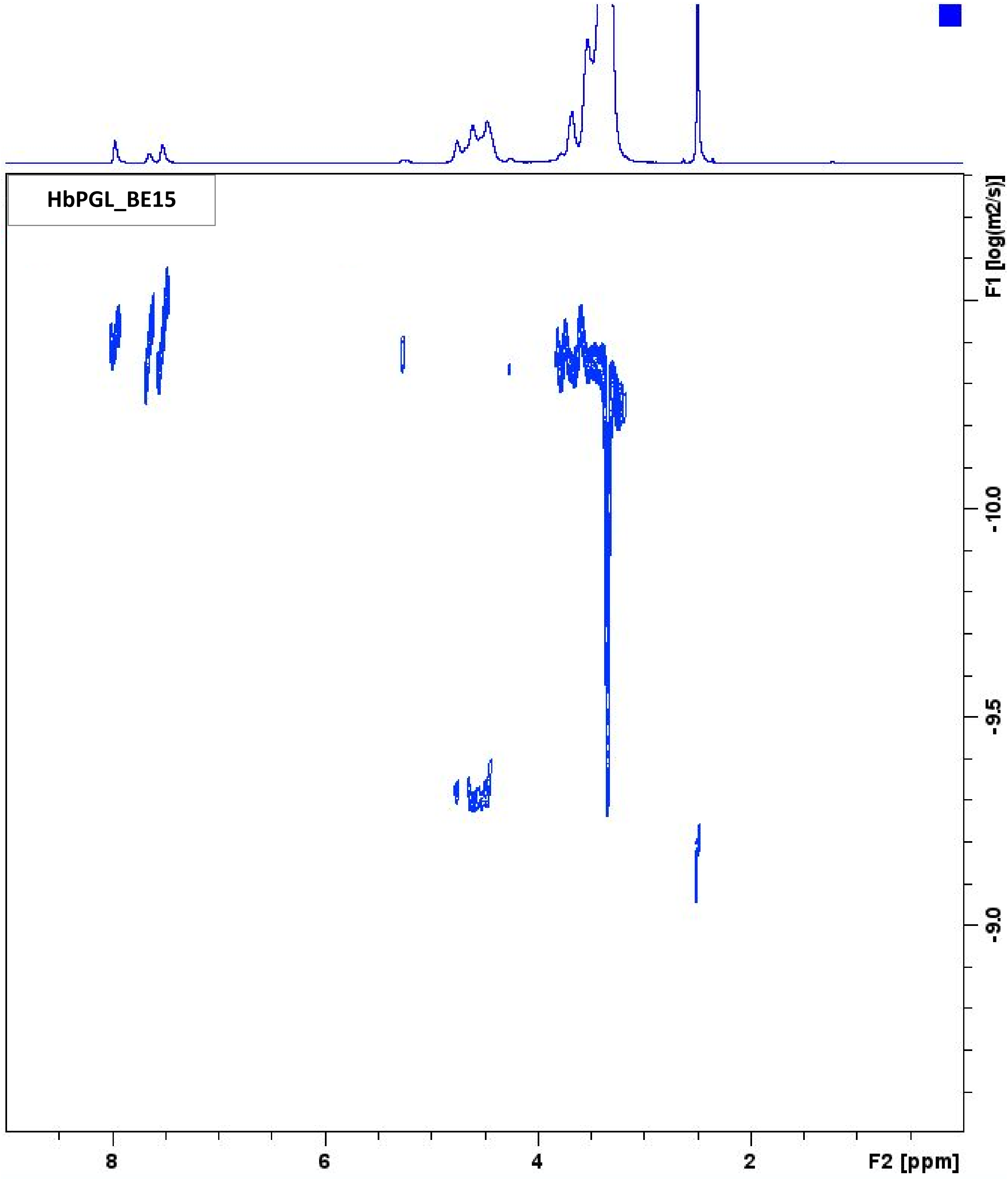

Figure S31. ^{1}H DOSY NMR spectrum of HbPGL_BE15 recorded in DMSO-d_6.

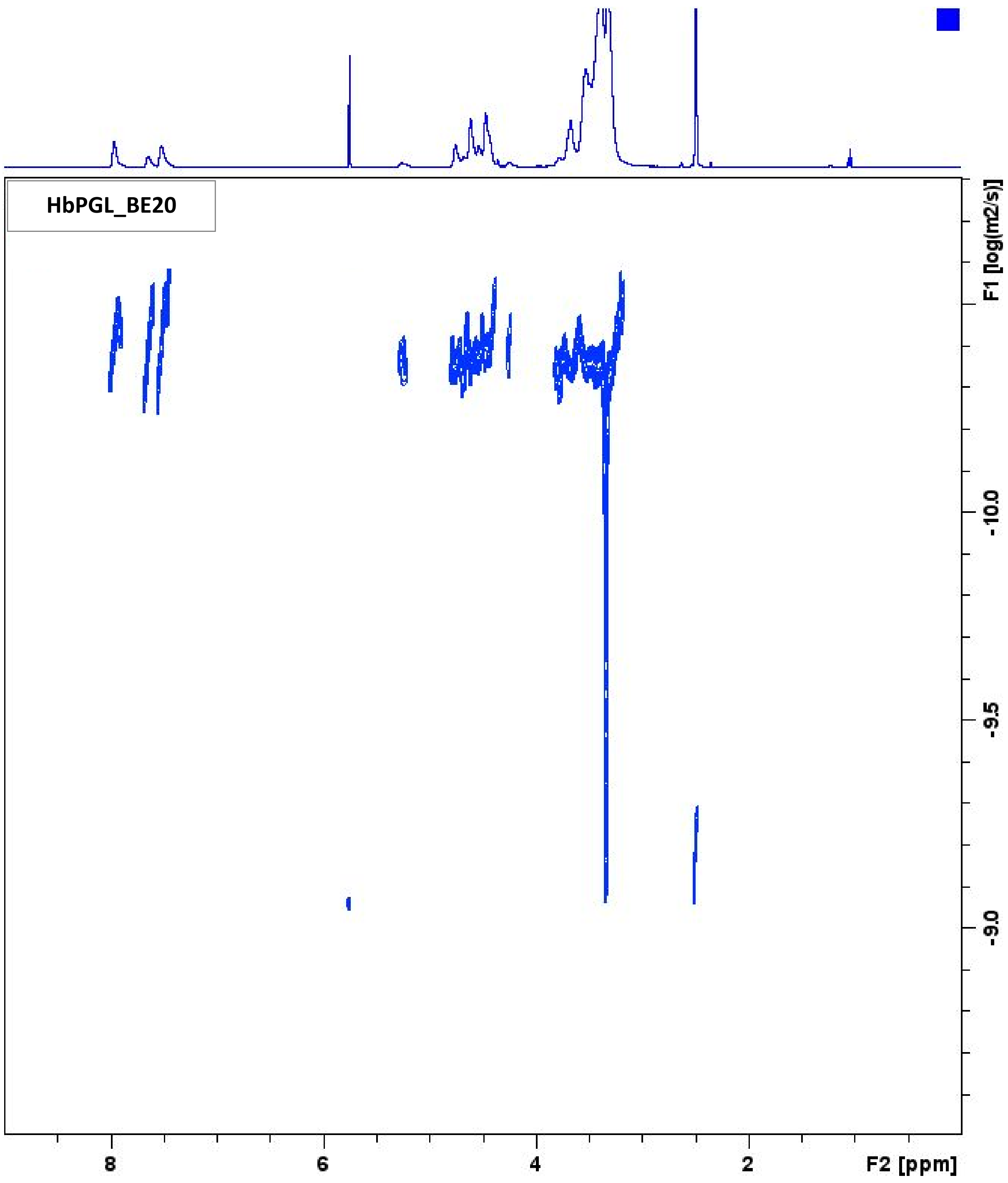

Figure S32. ^{1}H DOSY NMR spectrum of HbPGL_BE20 recorded in DMSO-d_6.

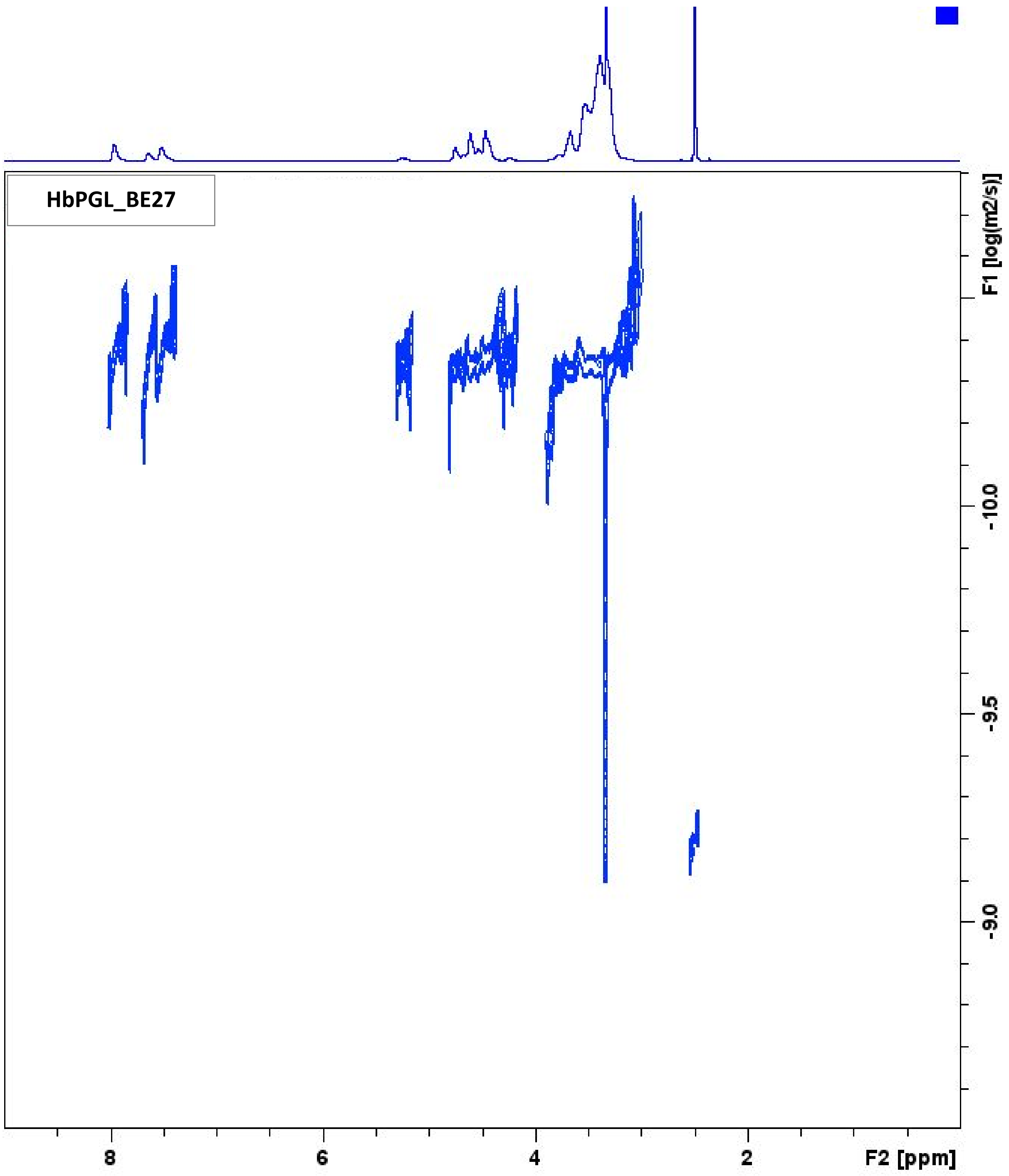

Figure S33. ^{1}H DOSY NMR spectrum of HbPGL_BE27 recorded in DMSO-d_6.

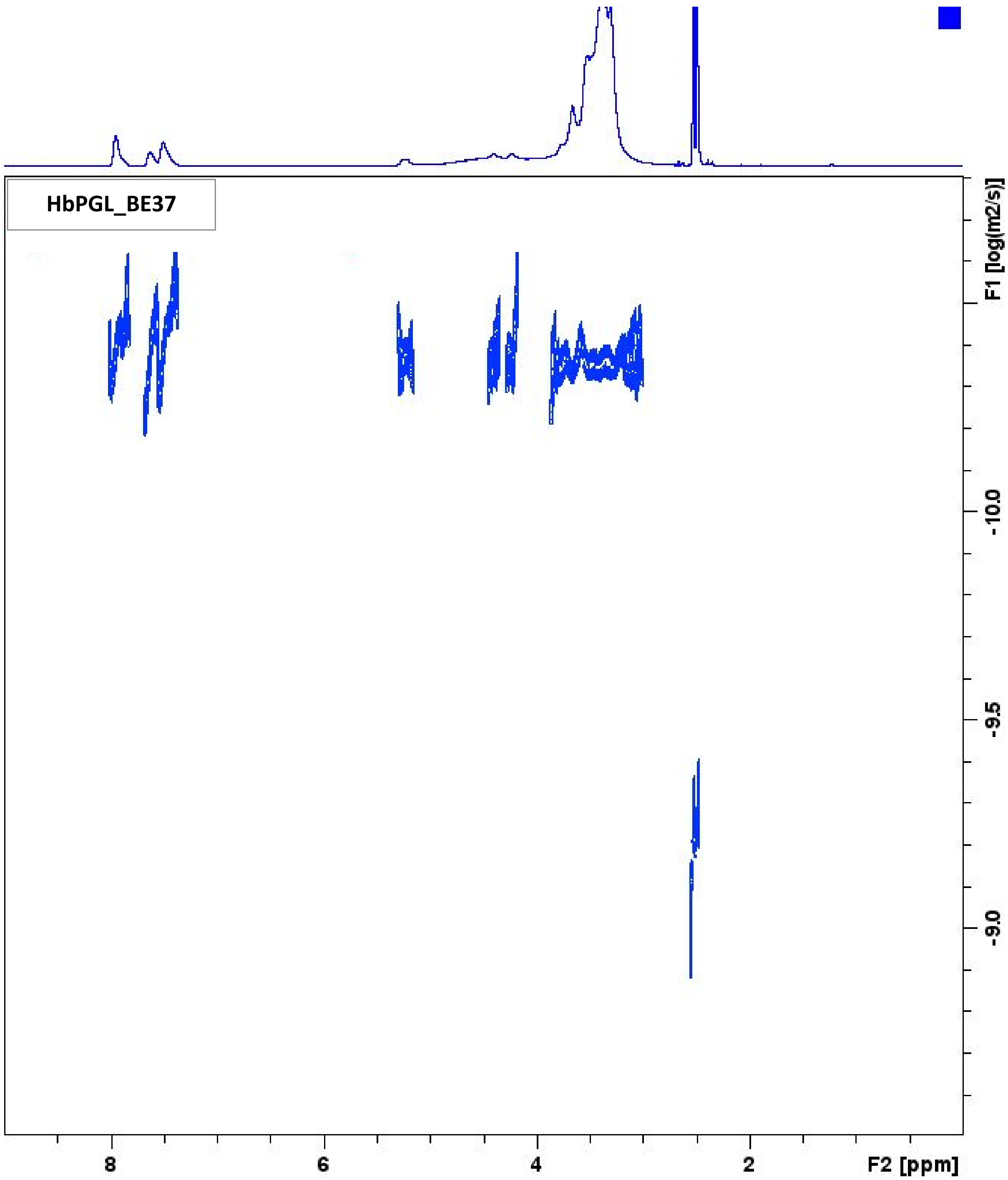

Figure S34. ^{1}H DOSY NMR spectrum of HbPGL_BE37 recorded in DMSO-d_6.

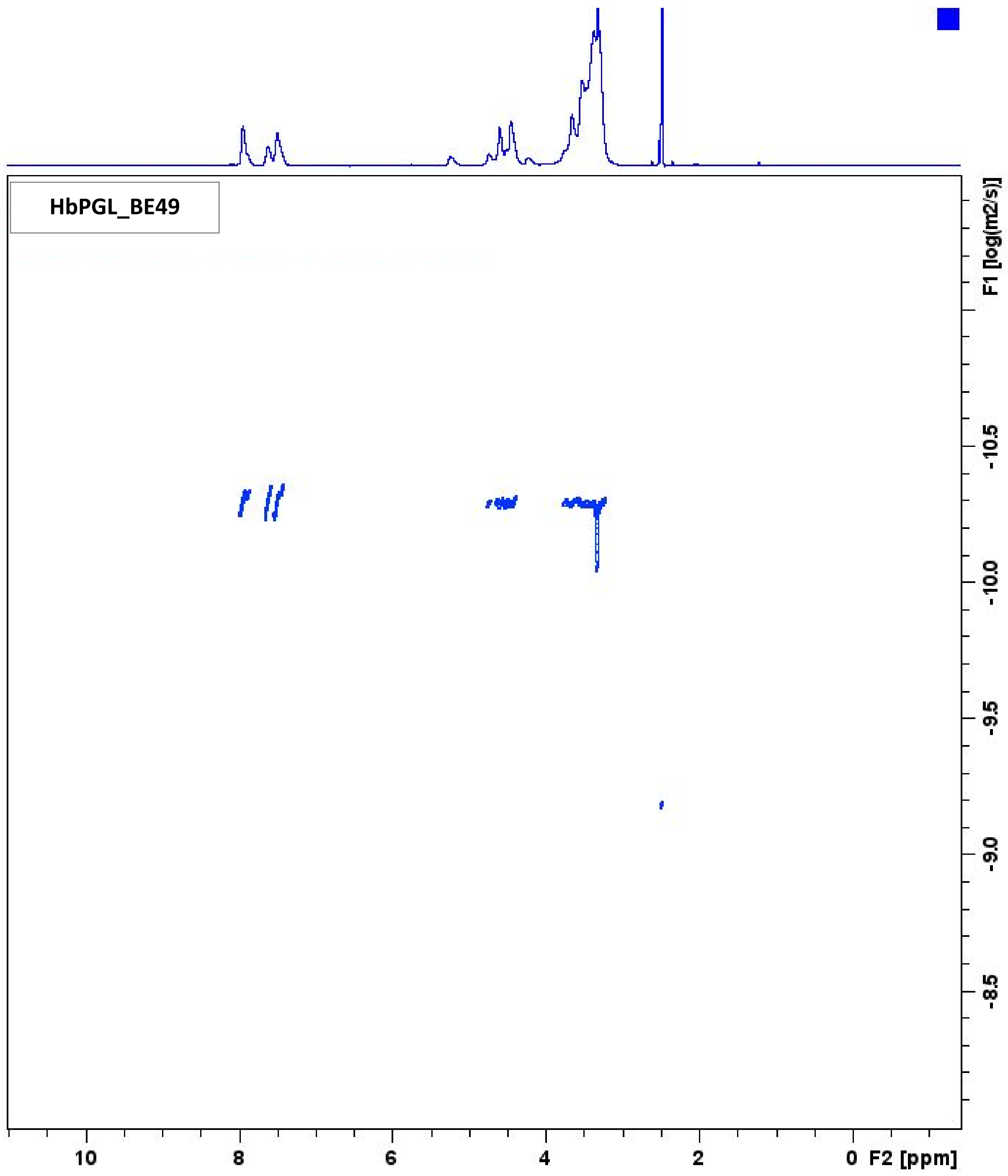

Figure S35. ^{1}H DOSY NMR spectrum of HbPGL_BE49 recorded in DMSO-d_6.

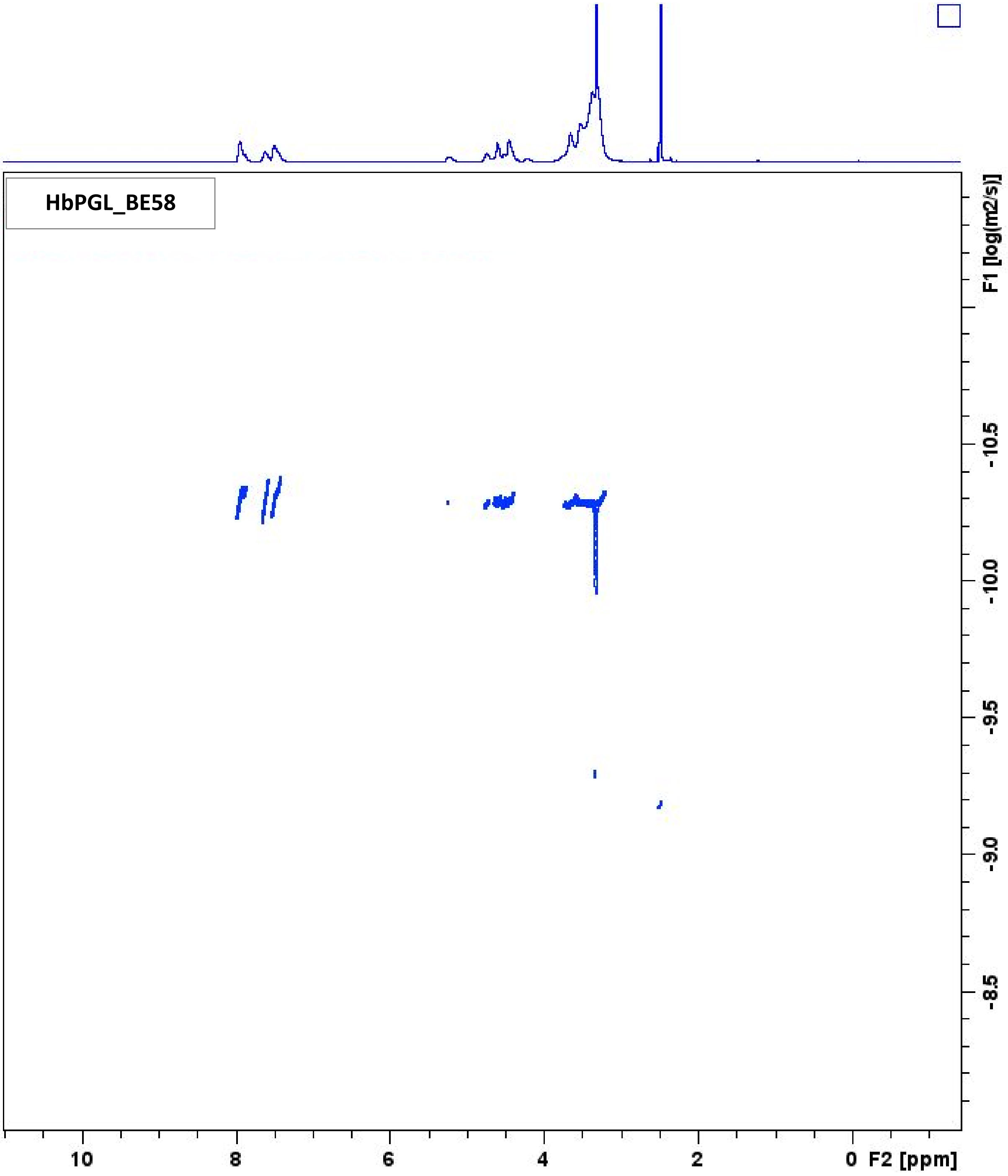

Figure S36. ^{1}H DOSY NMR spectrum of HbPGL_BE58 recorded in DMSO-d_6.

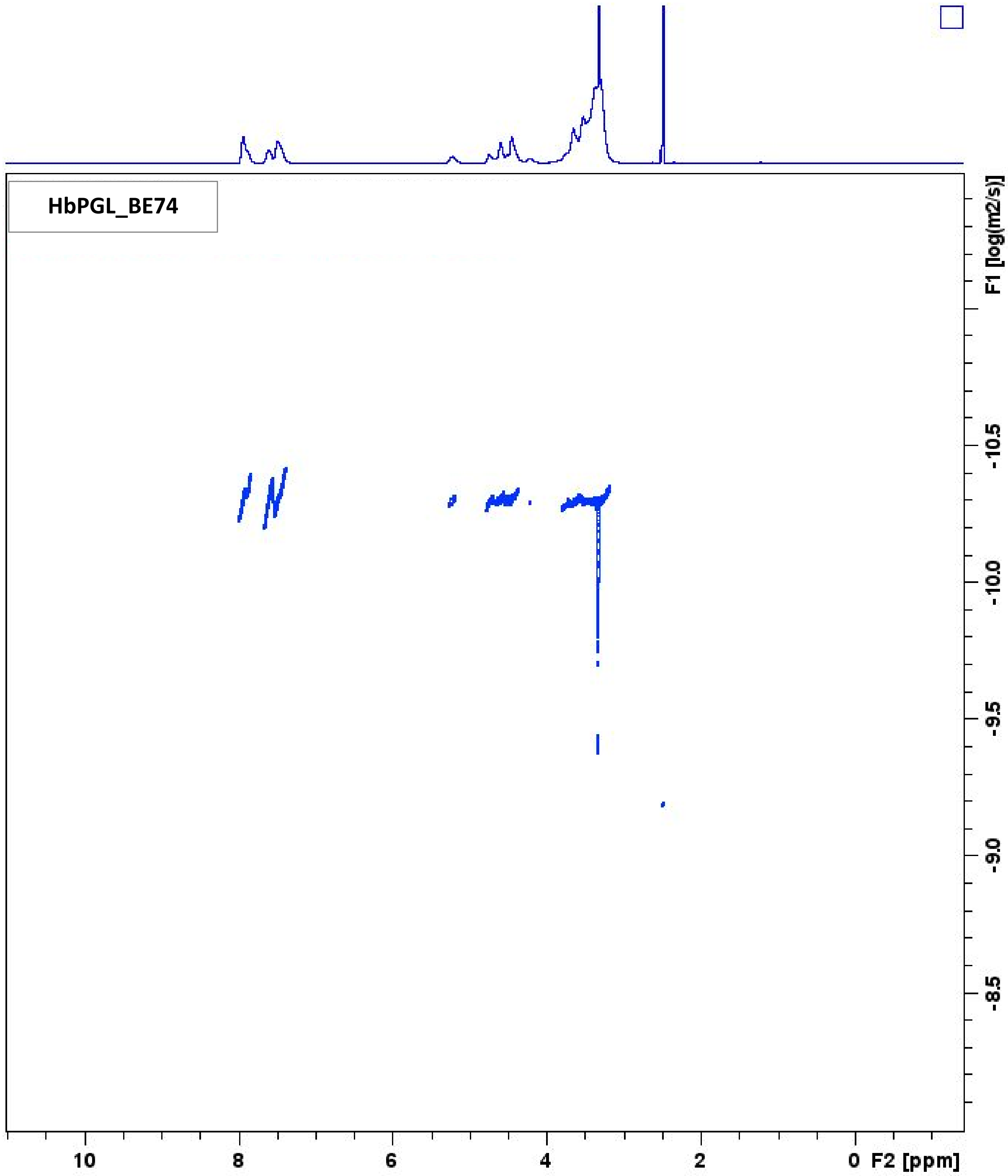

Figure S37. ^{1}H DOSY NMR spectrum of HbPGL_BE74 recorded in DMSO-d_6.

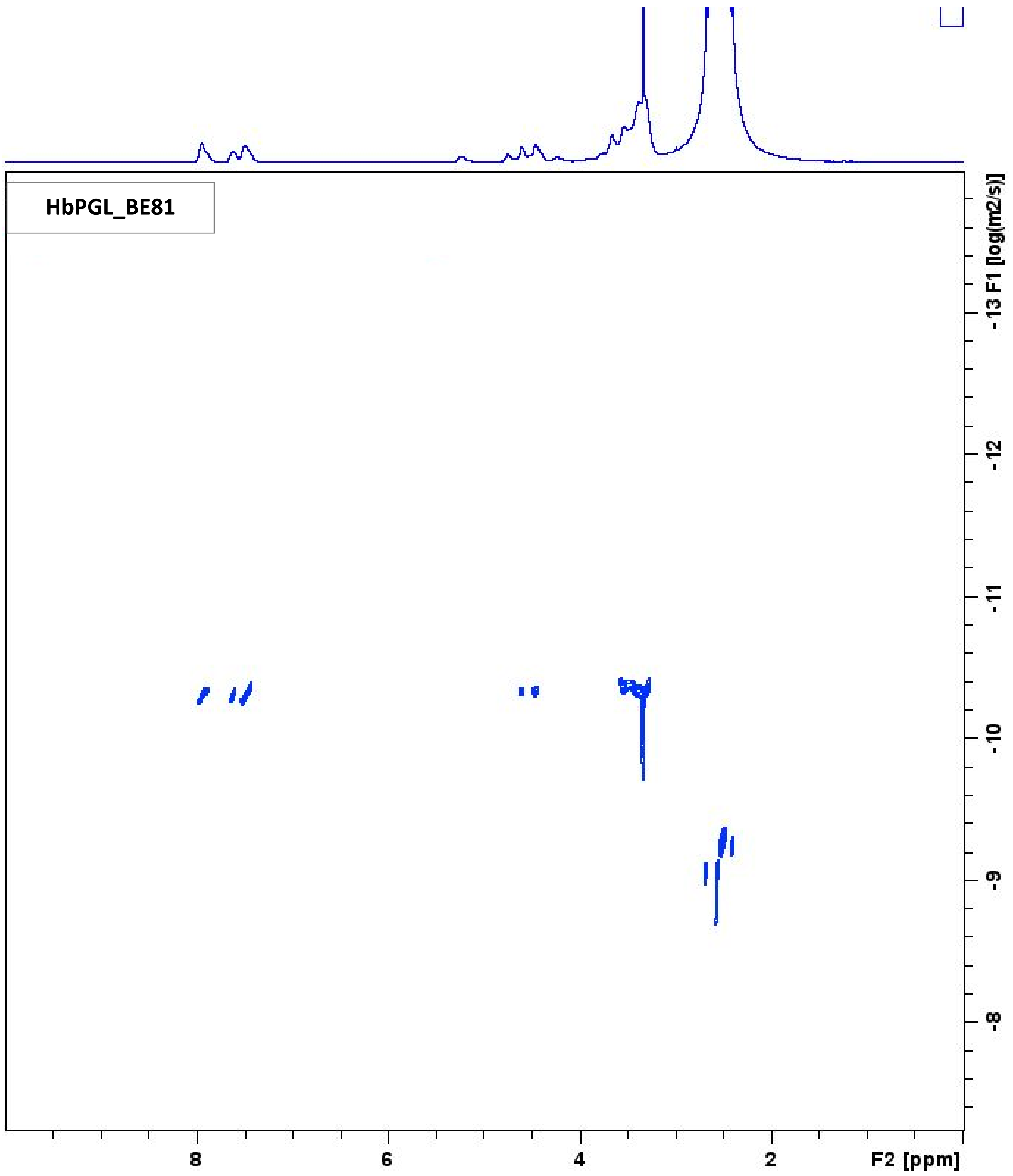

Figure S38. ^{1}H DOSY NMR spectrum of HbPGL_BE81 recorded in DMSO-d_6.

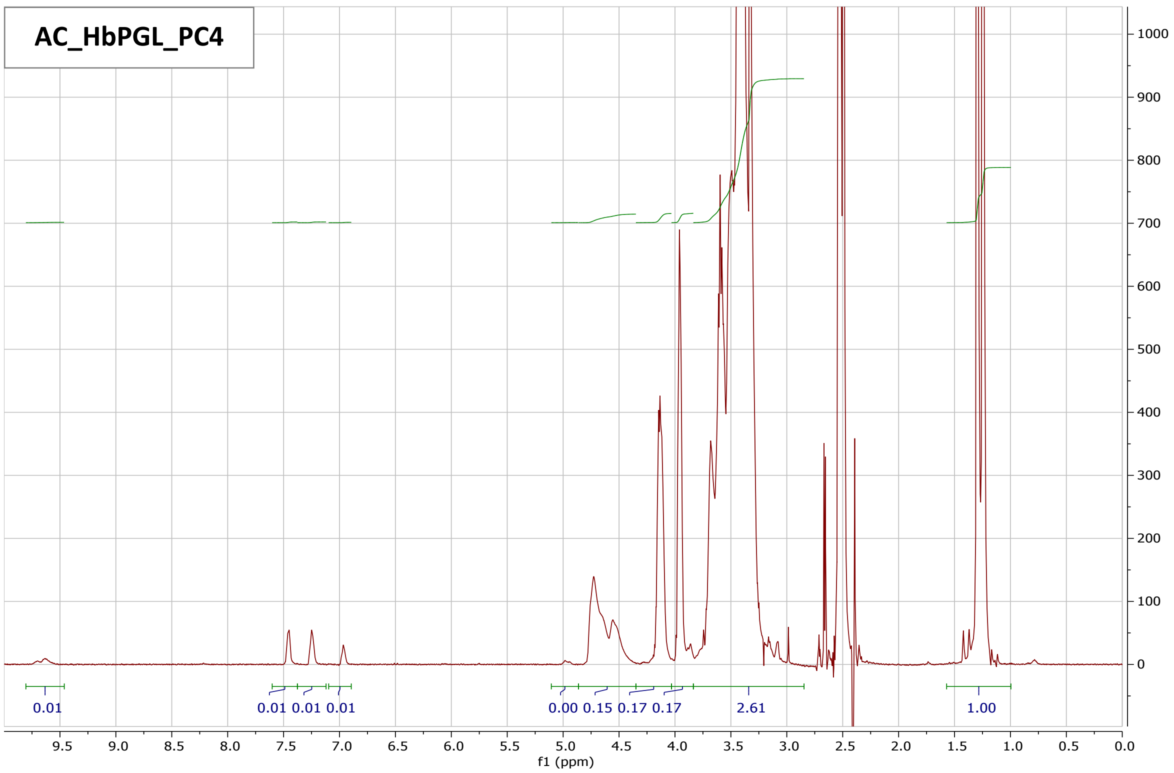

Figure S39. ^{1}H NMR spectrum of AC_HbPGL_PC4 recorded in DMSO-d_6.

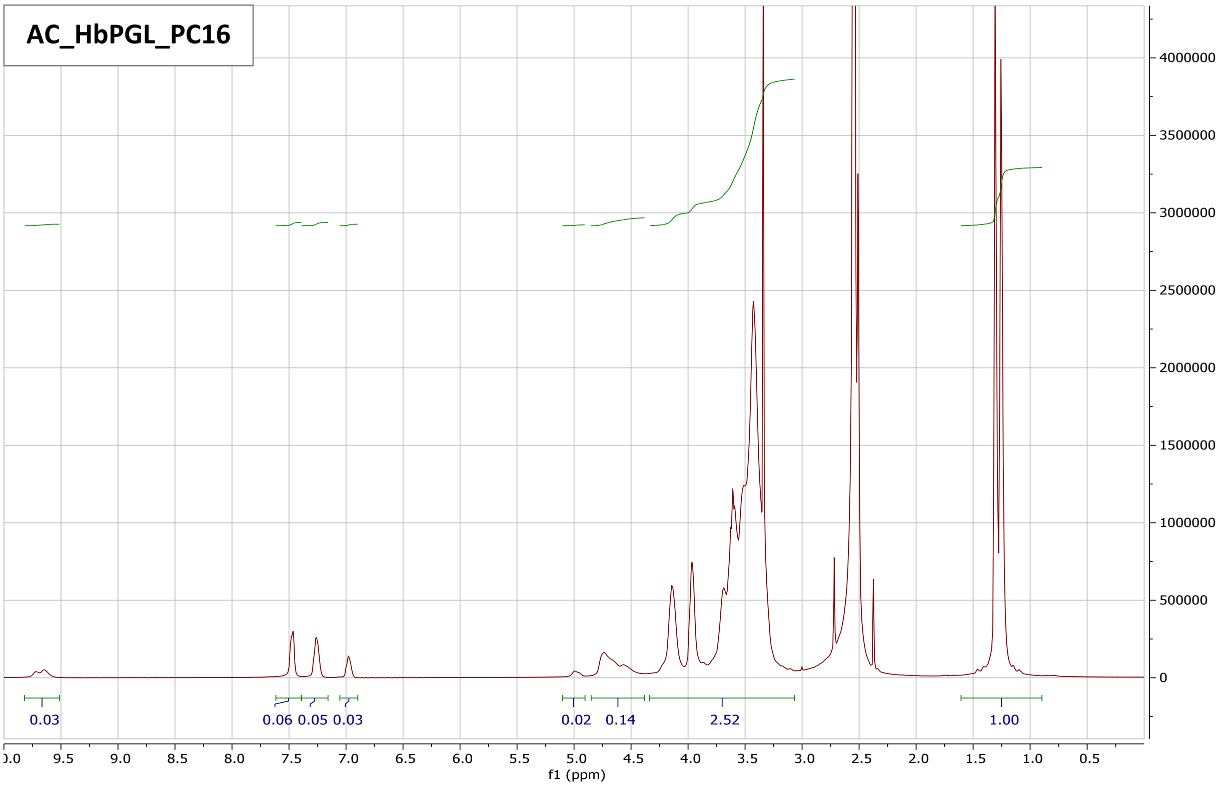

Figure S40. ^{1}H NMR spectrum of AC_HbPGL_PC16 recorded in DMSO-d_6.

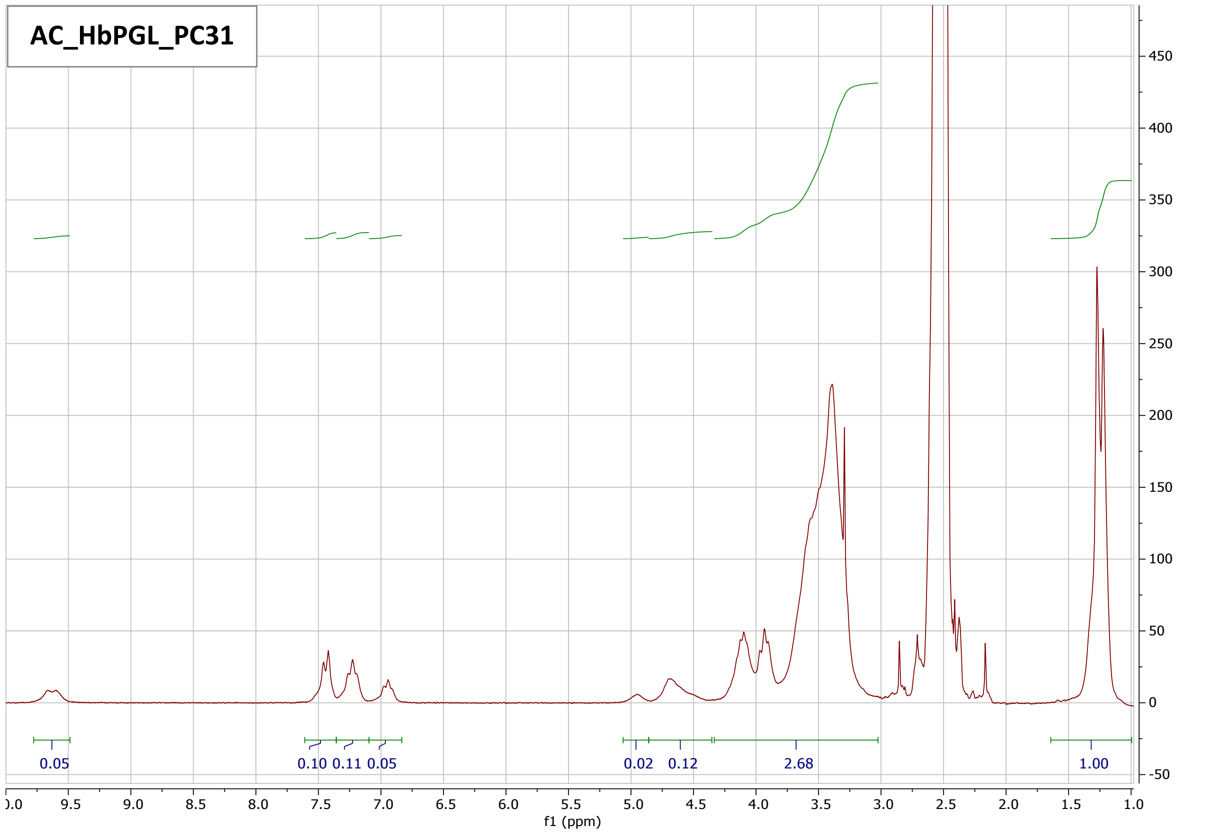

Figure S41. ^{1}H NMR spectrum of AC_HbPGL_PC31 recorded in DMSO-d_6.

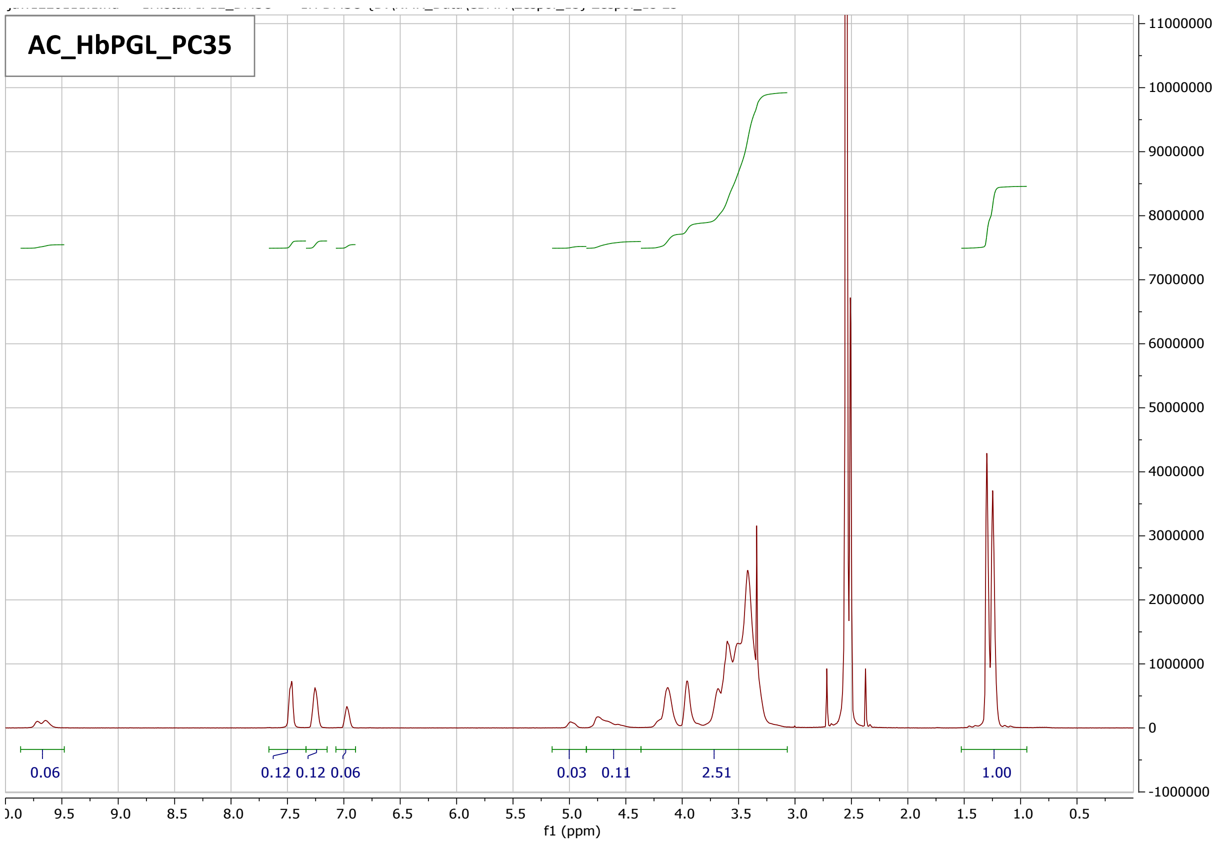

Figure S42. ^{1}H NMR spectrum of AC_HbPGL_PC35 recorded in DMSO-d_6.

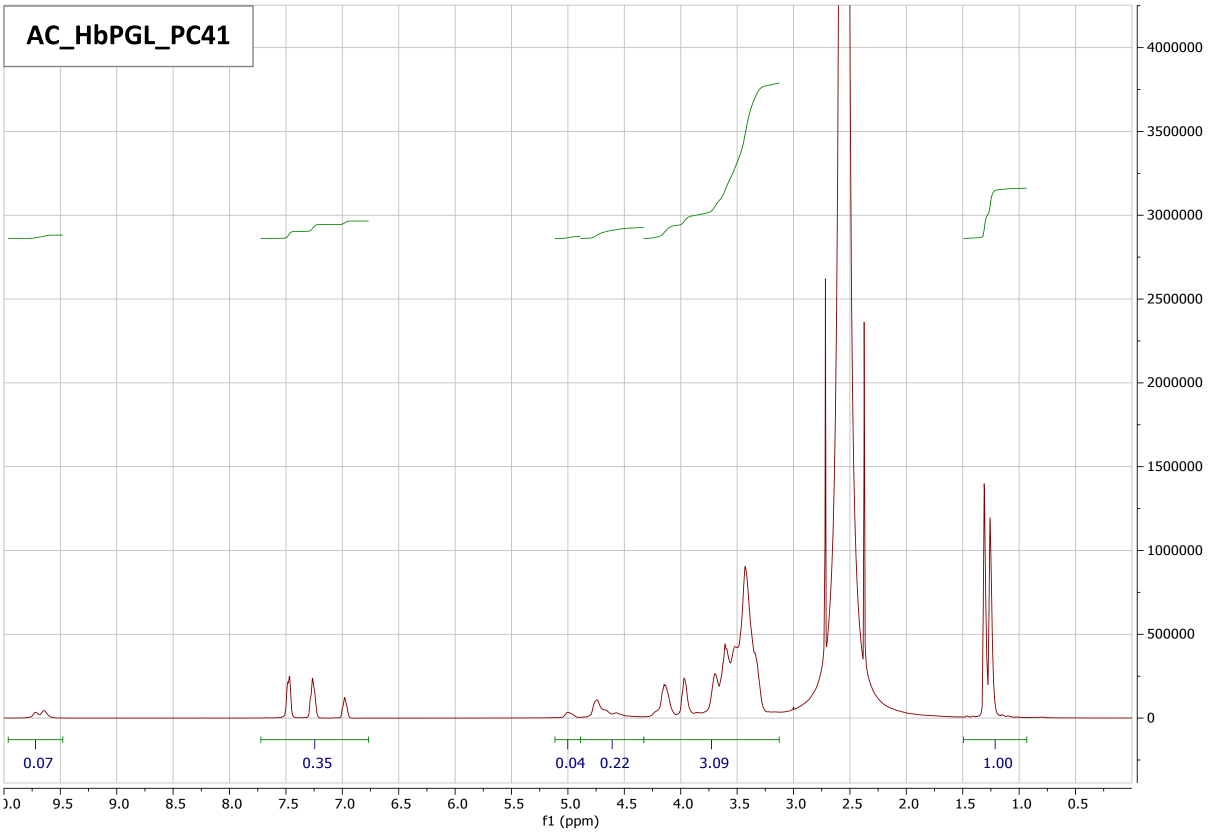

Figure S43. ^{1}H NMR spectrum of AC_HbPGL_PC41 recorded in DMSO-d_6.

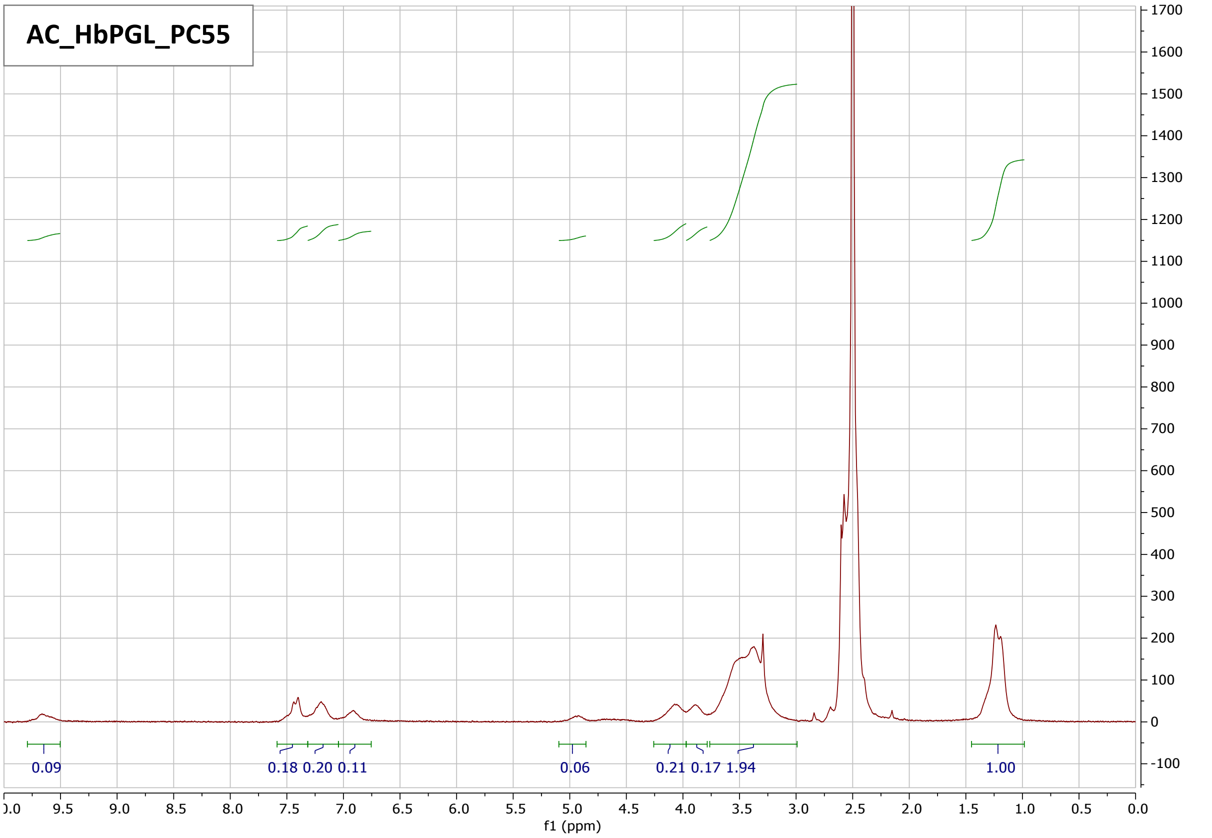

Figure S44. ^{1}H NMR spectrum of AC_HbPGL_PC55 recorded in DMSO-d_6.

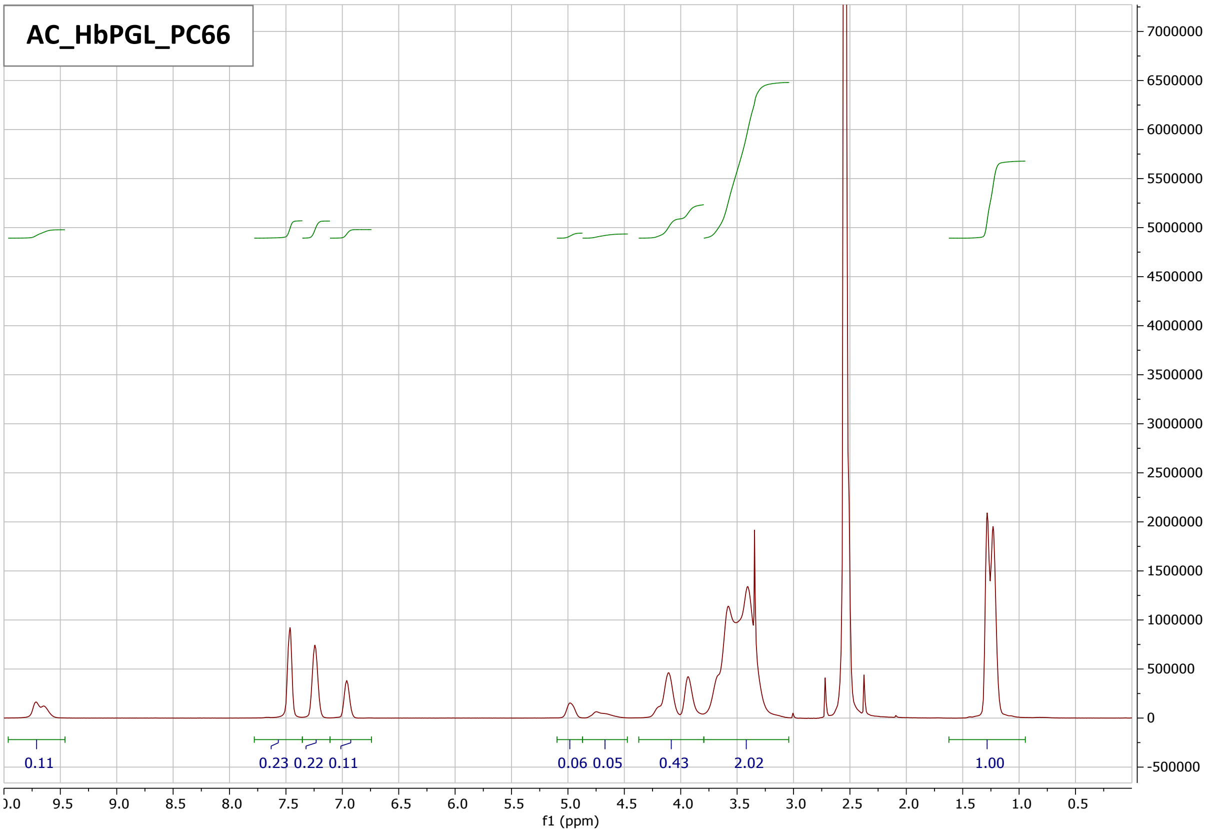

Figure S45. ^{1}H NMR spectrum of AC_HbPGL_PC66 recorded in DMSO-d$_6$.

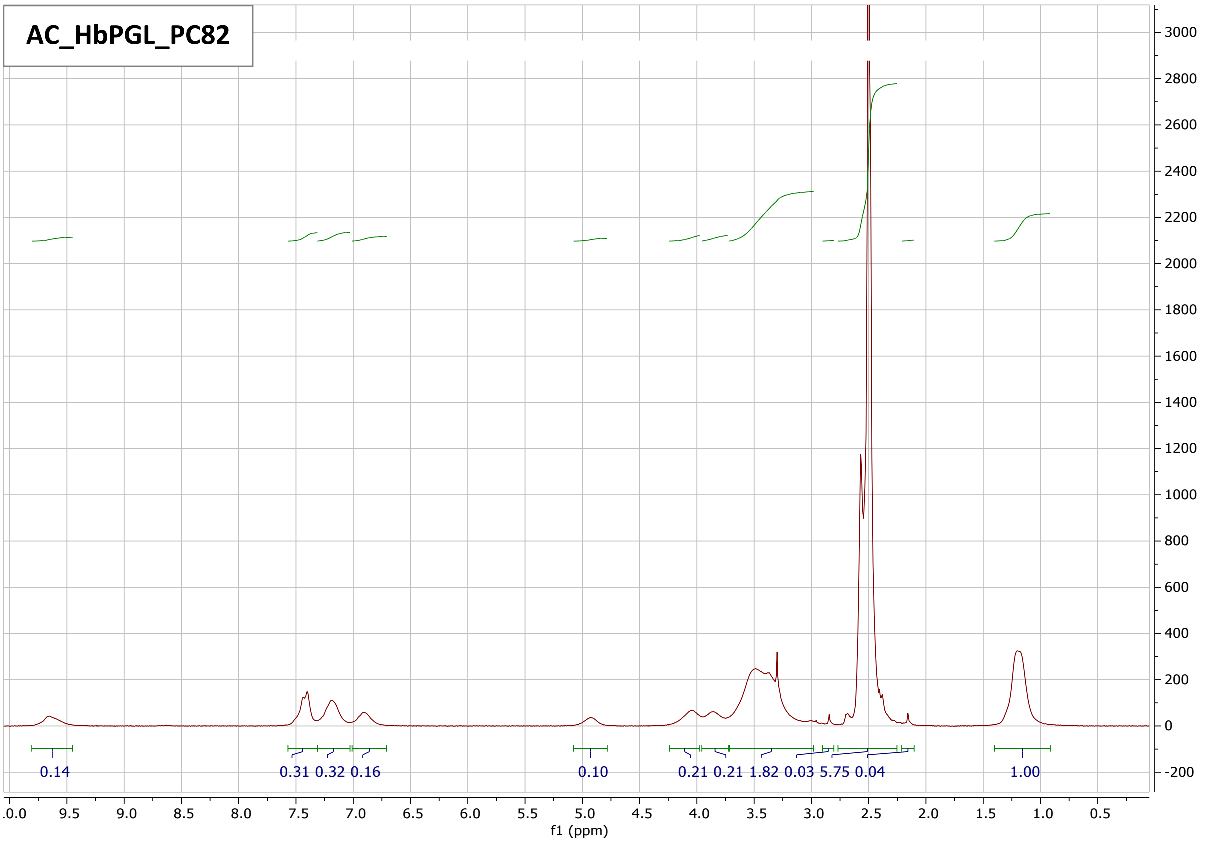

Figure S46. ^{1}H NMR spectrum of AC_HbPGL_PC82 recorded in DMSO-d$_6$.

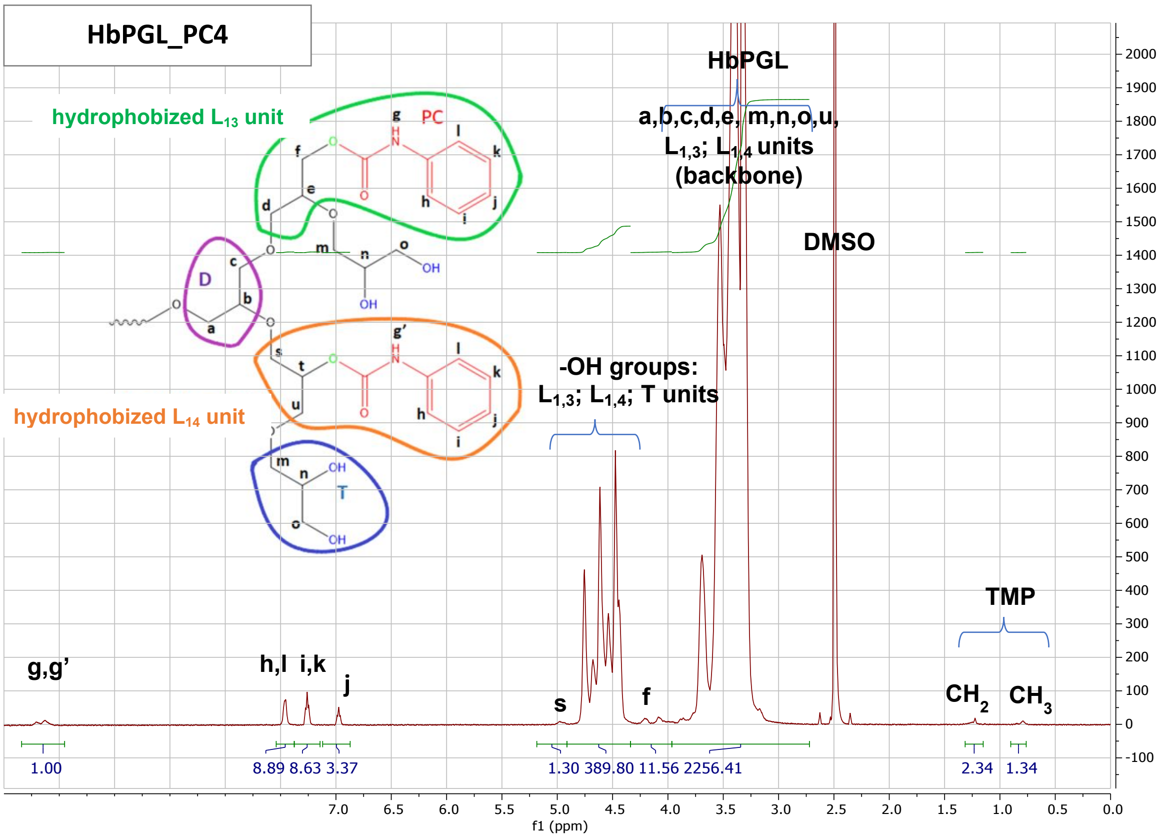

Figure S47. ^{1}H NMR spectrum of HbPGL_PC4 recorded in DMSO-d_6.

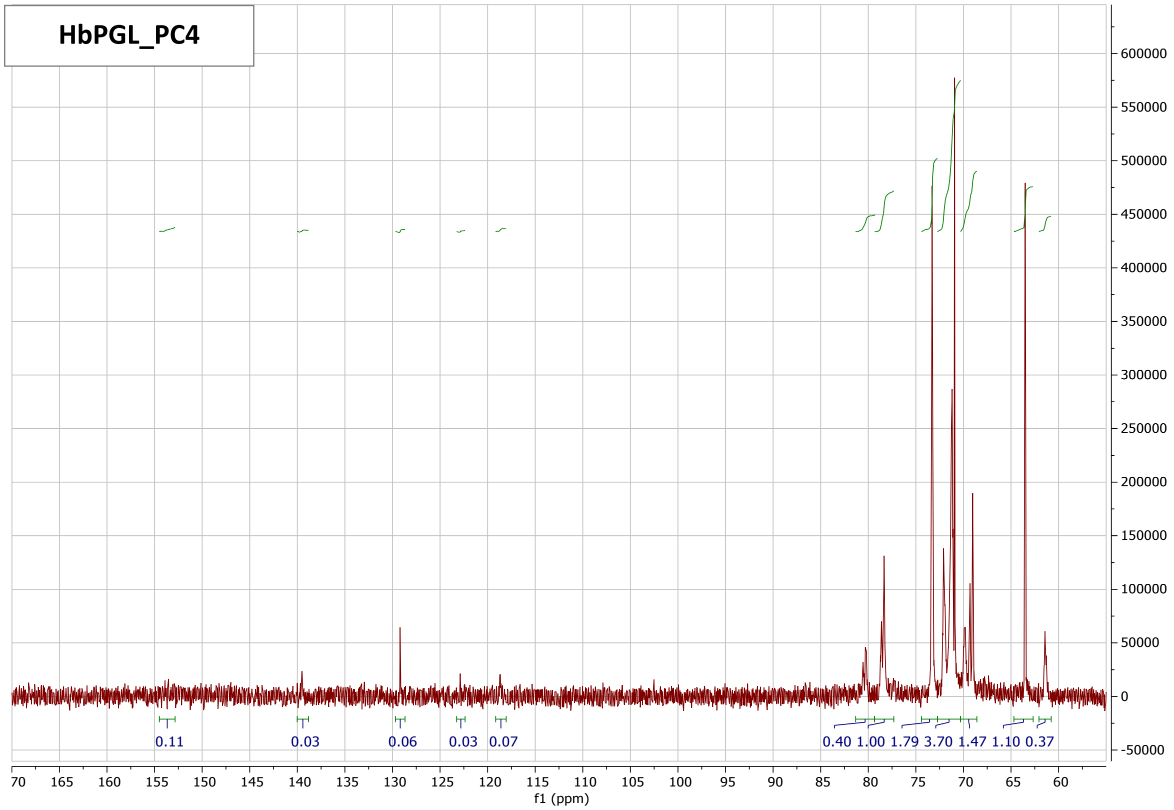

Figure S48. ^{13}C INVGATED NMR spectrum of HbPGL_PC4 recorded in DMSO-d_6

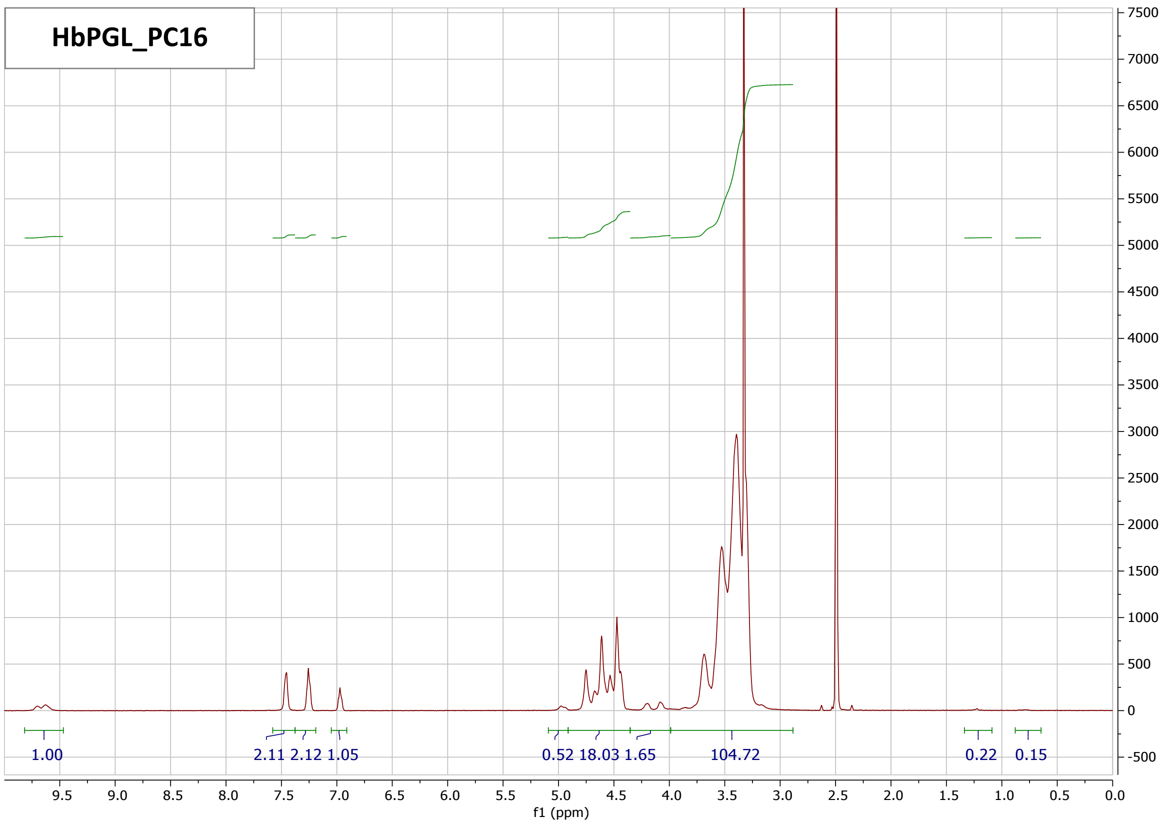

Figure S49. ^{1}H NMR spectrum of HbPGL_PC16 recorded in DMSO-d_6.

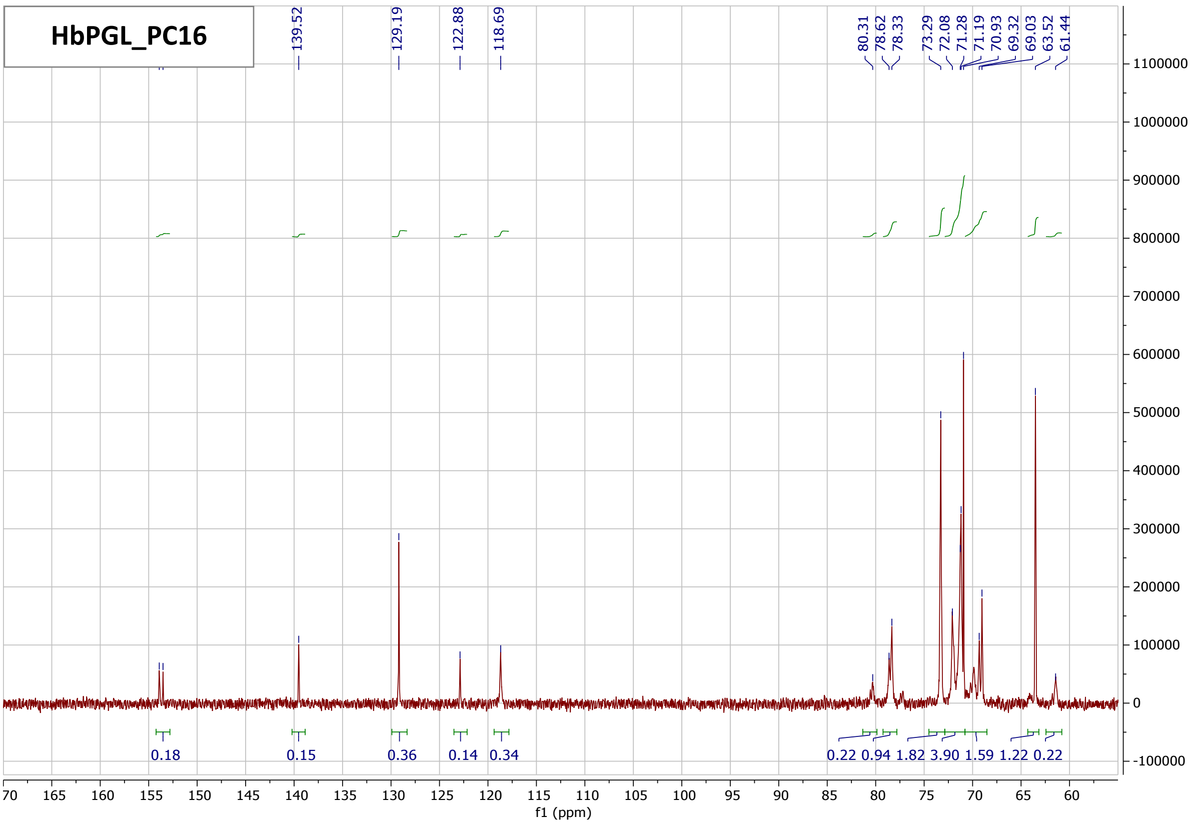

Figure S50. ^{13}C INVGATED NMR spectrum of HbPGL_PC16 recorded in DMSO-d_6.

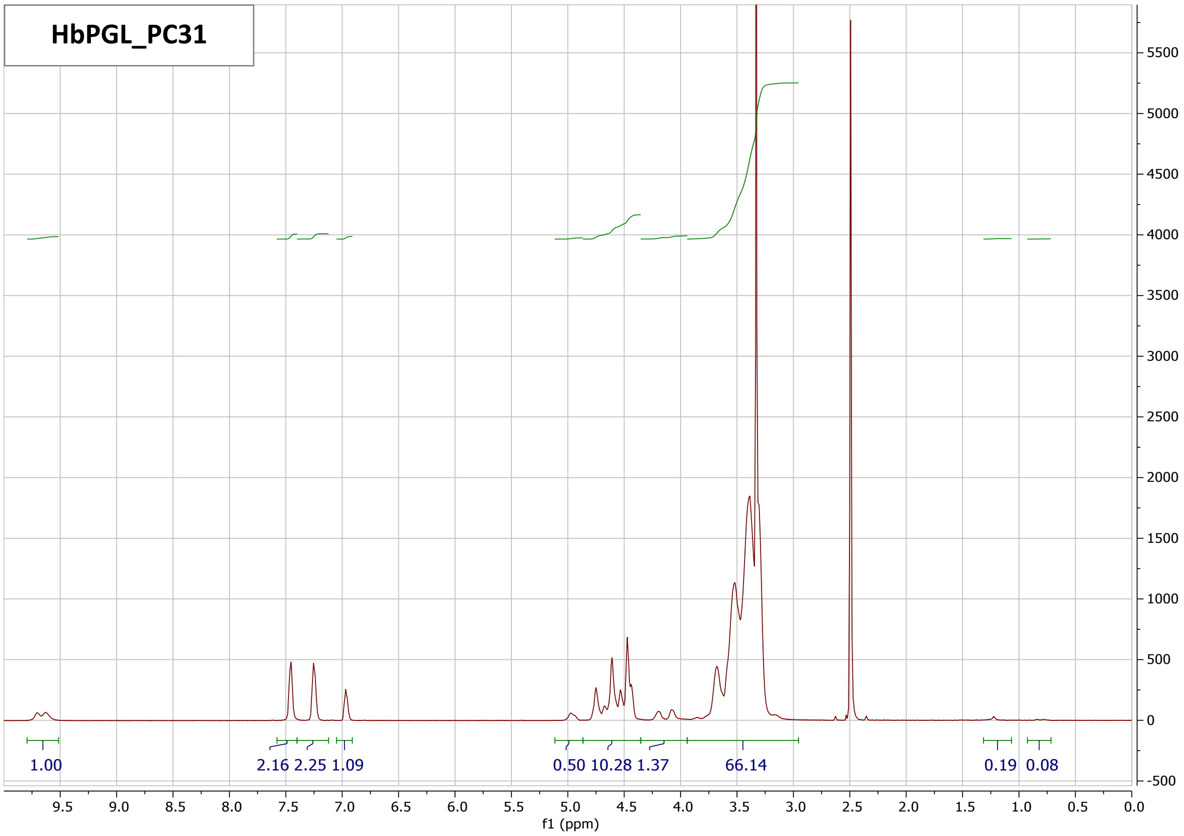

Figure S51. ^{1}H NMR spectrum of HbPGL_PC31 recorded in DMSO-d$_6$.

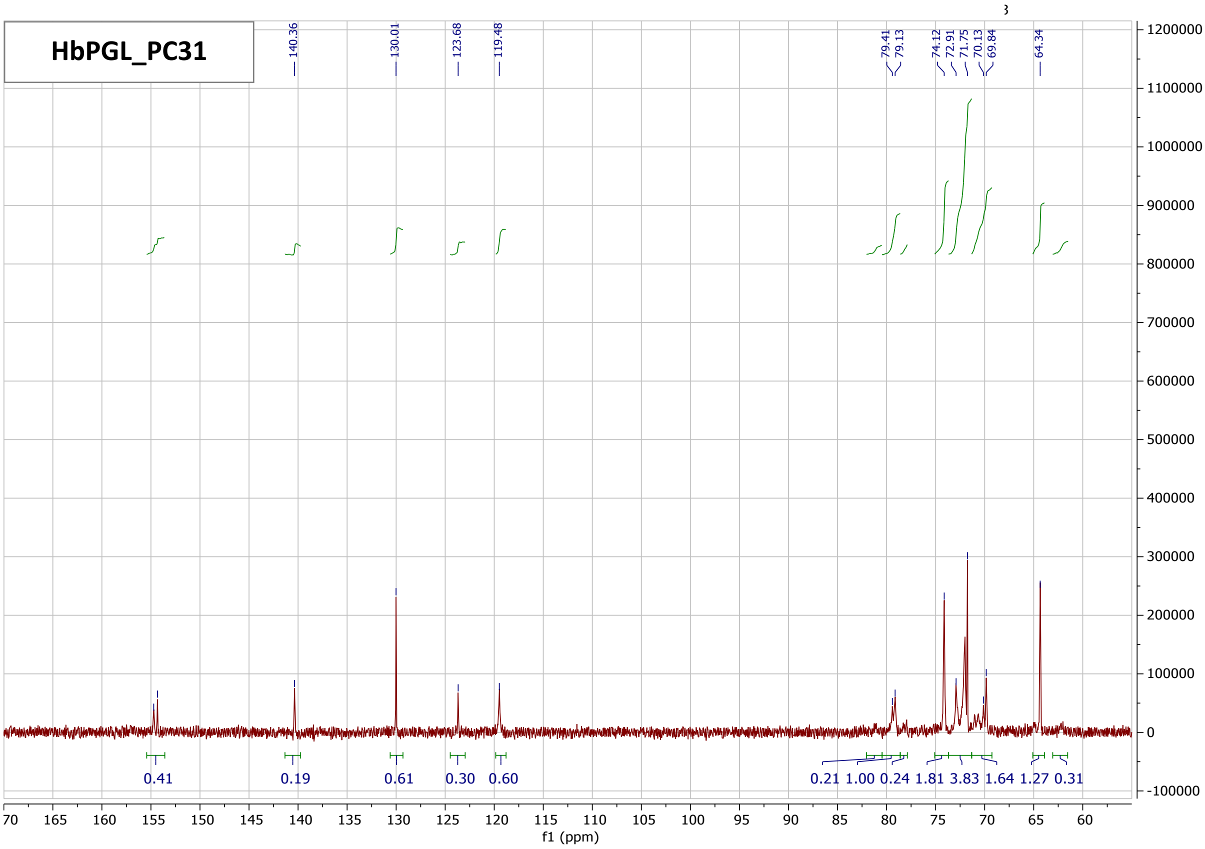

Figure S52. ^{13}C INVGATED NMR spectrum of HbPGL_PC31 recorded in DMSO-d$_6$.

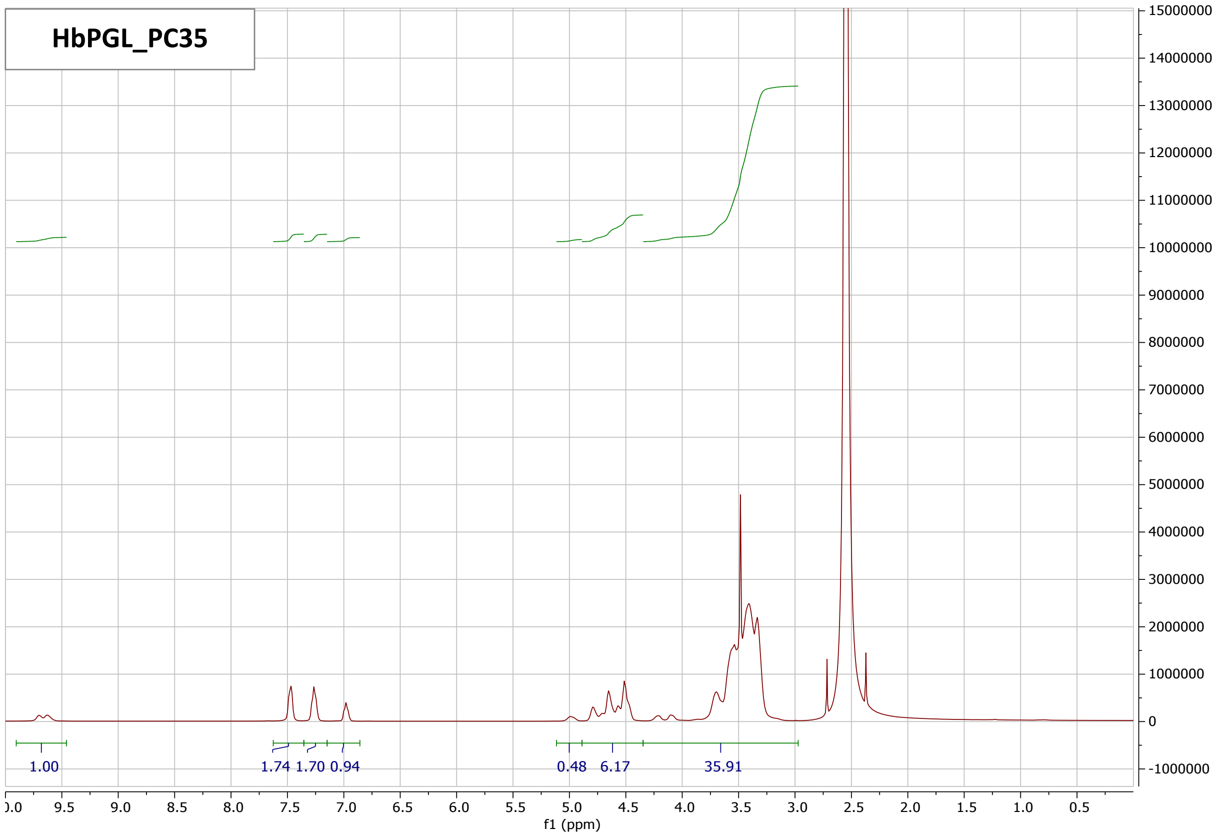

Figure S53. ^{1}H NMR spectrum of HbPGL_PC35 recorded in DMSO-d_6.

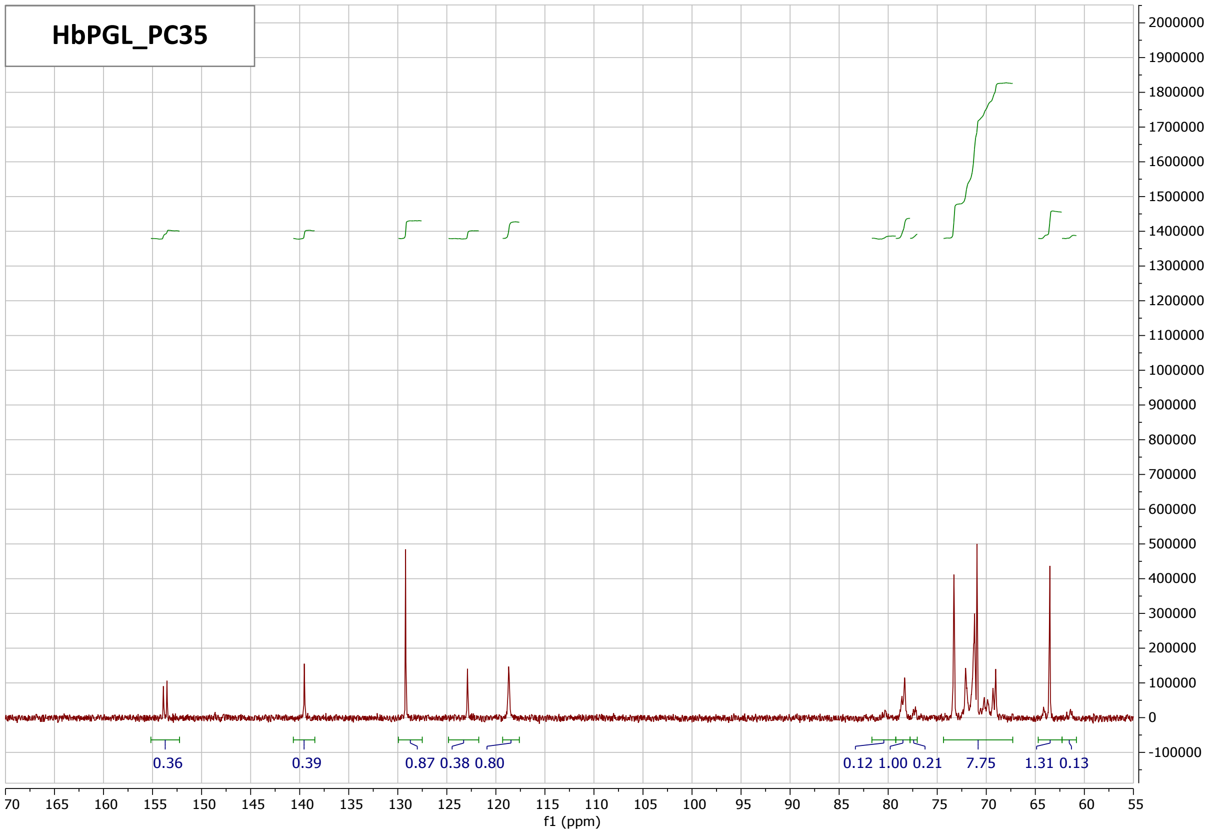

Figure S54. ^{13}C INVGATED NMR spectrum of HbPGL_PC35 recorded in DMSO-d_6.

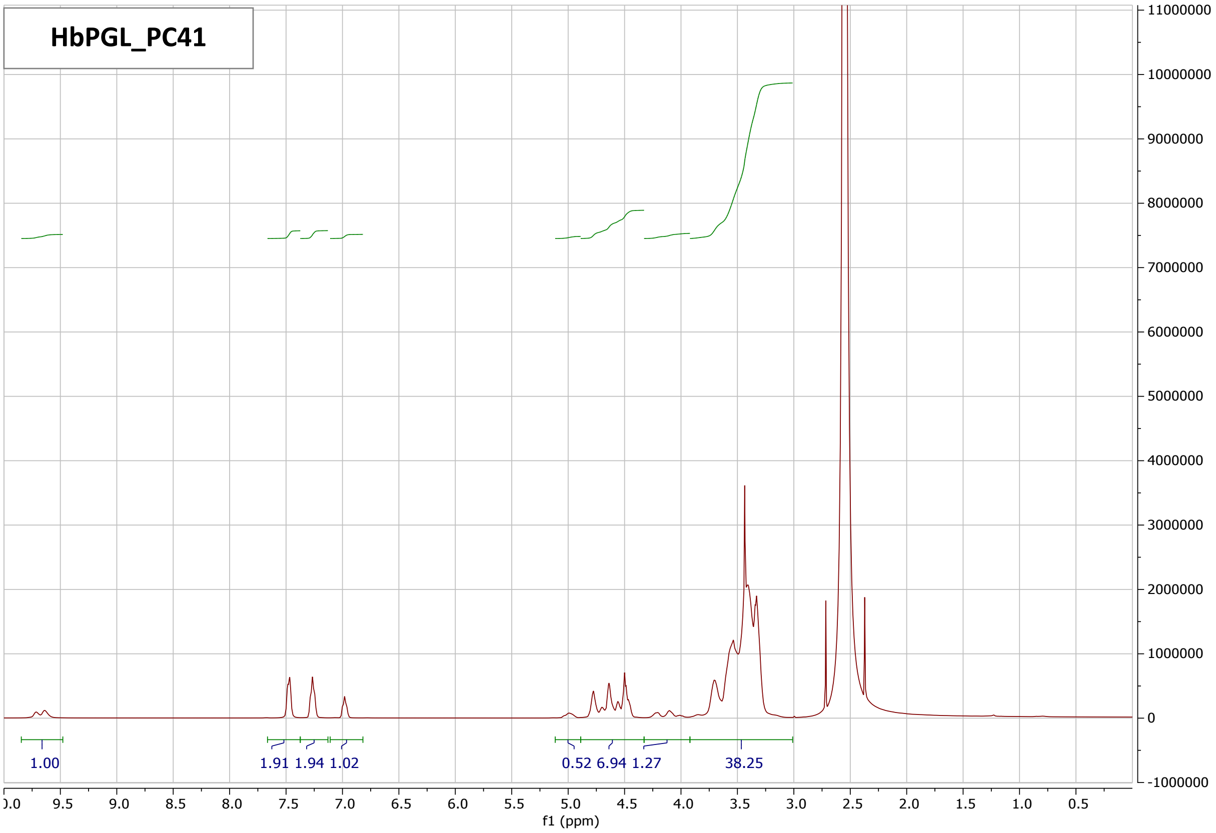

Figure S55. ^{1}H NMR spectrum of HbPGL_PC41 recorded in DMSO-d$_6$.

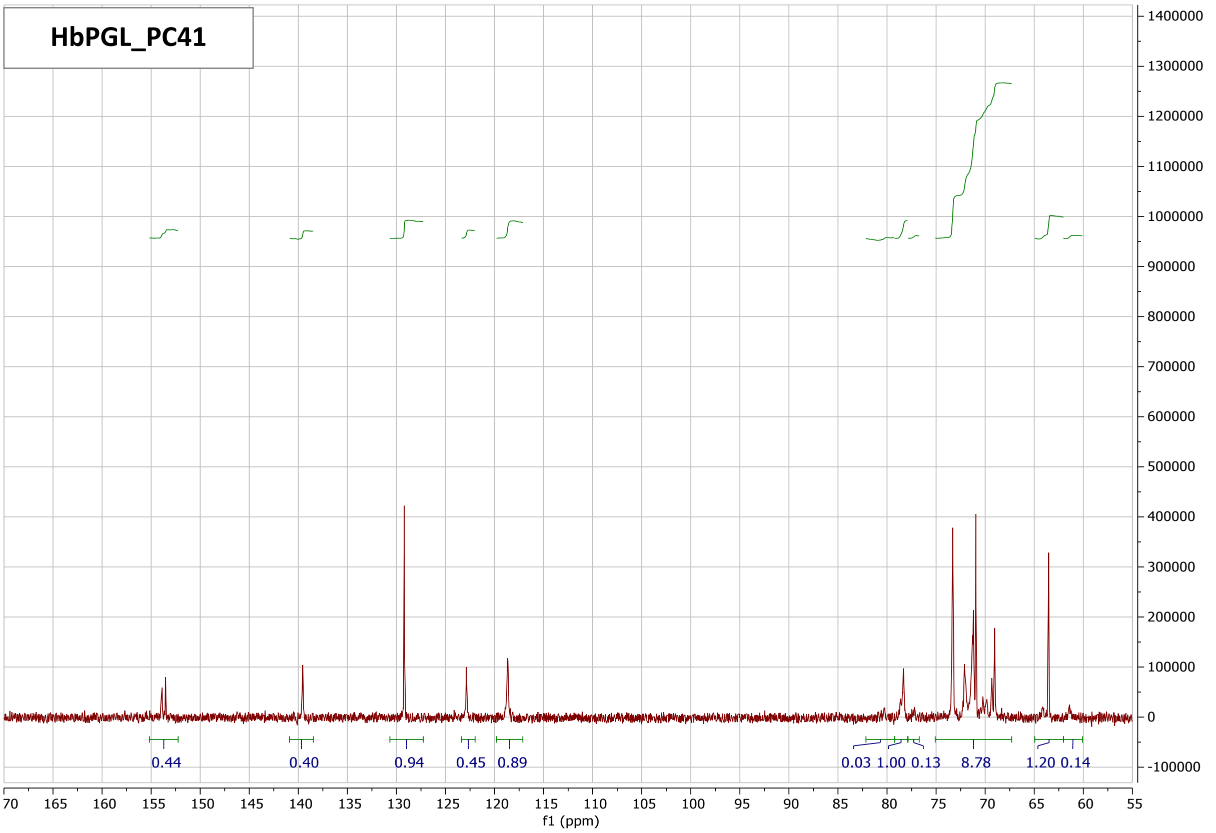

FigureS56. ^{13}C INVGATED NMR spectrum of HbPGL_PC41 recorded in DMSO-d$_6$.

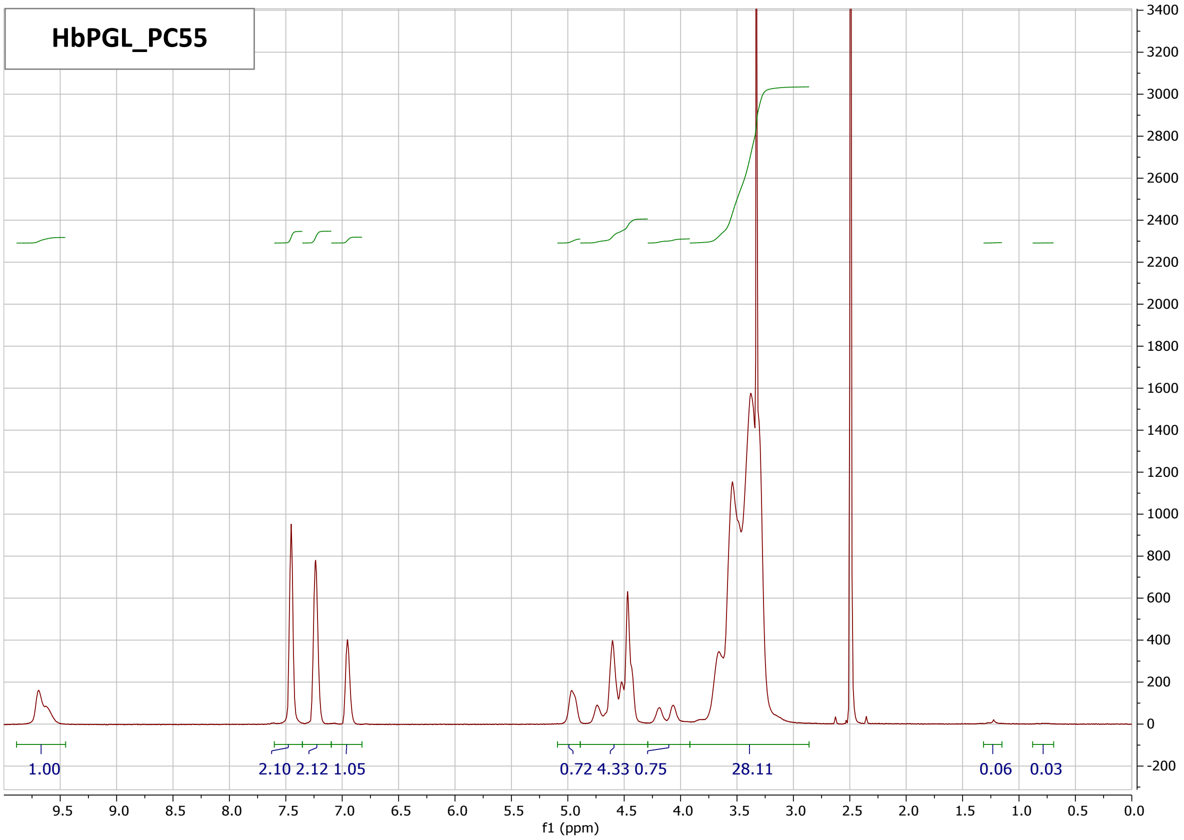

Figure S57. ^{1}H NMR spectrum of HbPGL_PC55 recorded in DMSO-d_6.

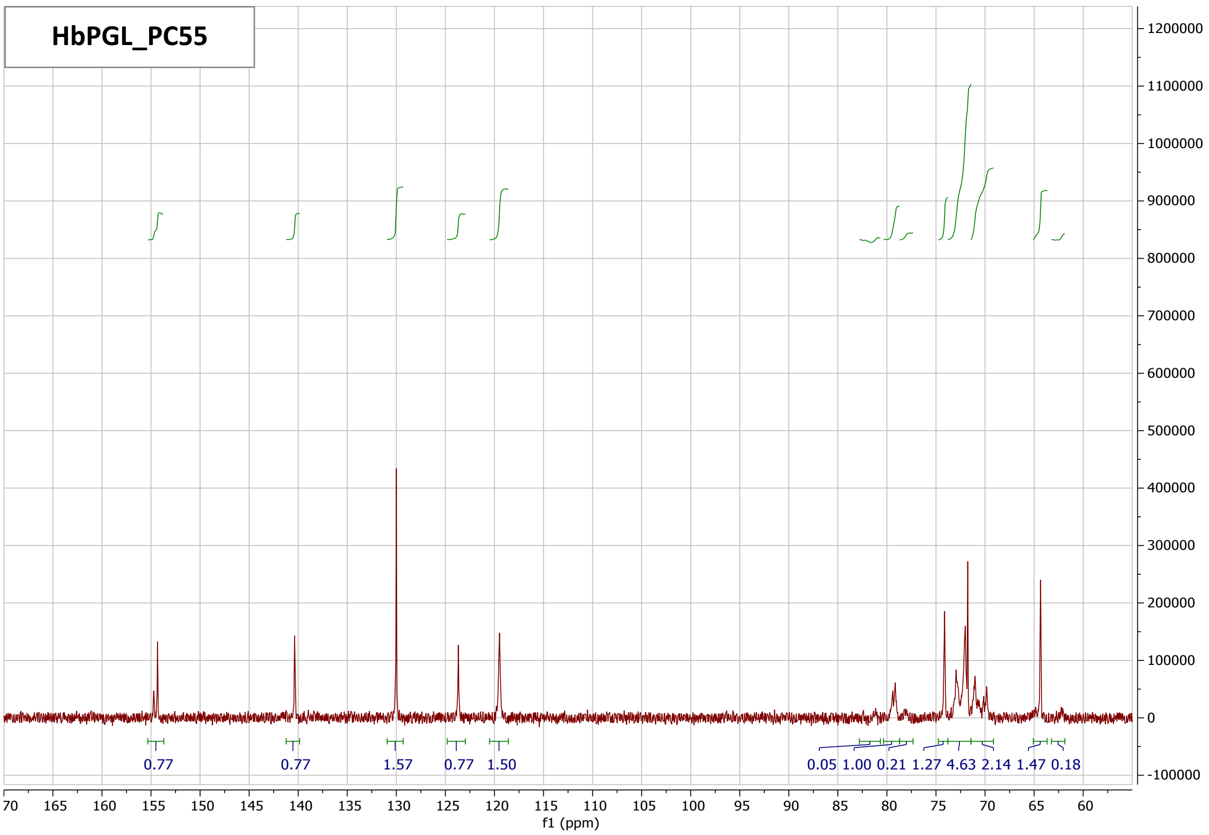

Figure S58. ^{13}C INVGATED NMR spectrum of HbPGL_PC55 recorded in DMSO-d_6.

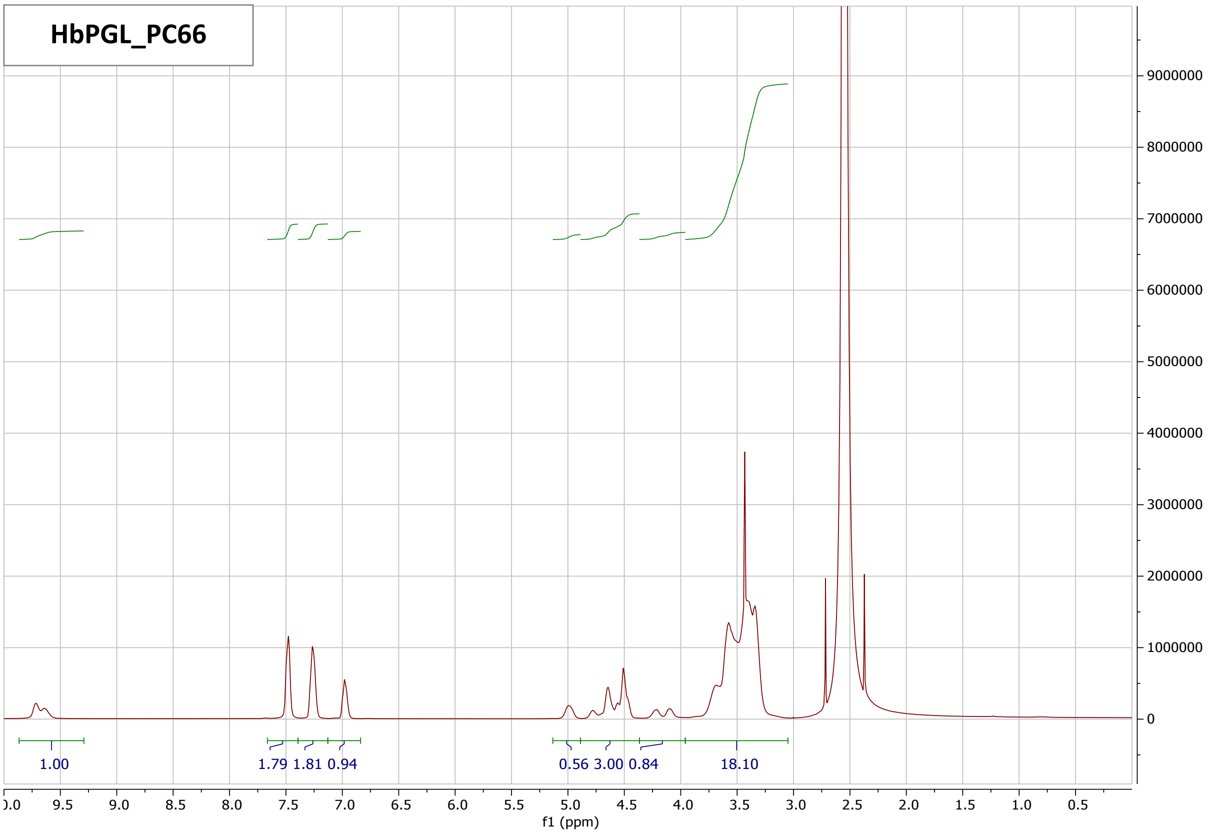

Figure S59. ^{1}H NMR spectrum of HbPGL_PC66 recorded in DMSO-d_6.

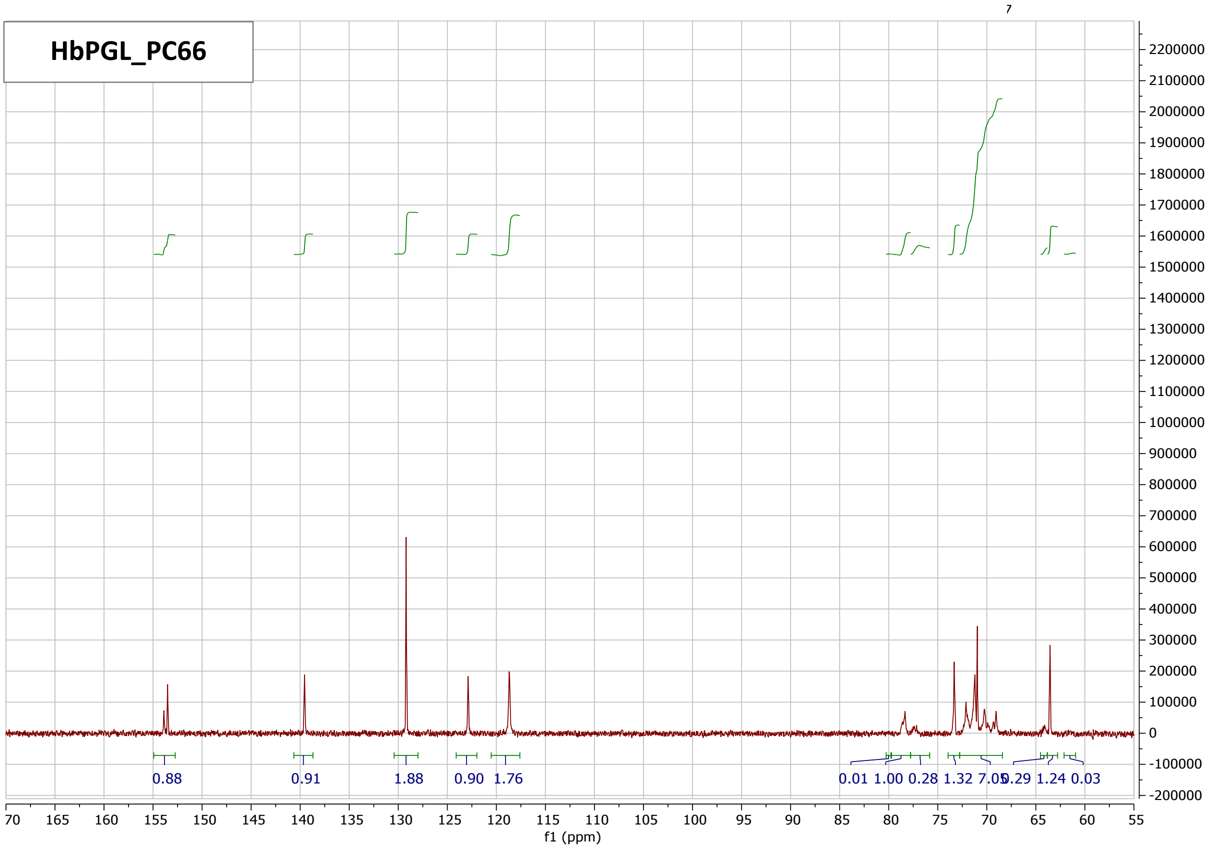

Figure S60. ^{13}C INVGATED NMR spectrum of HbPGL_PC66 recorded in DMSO-d_6.

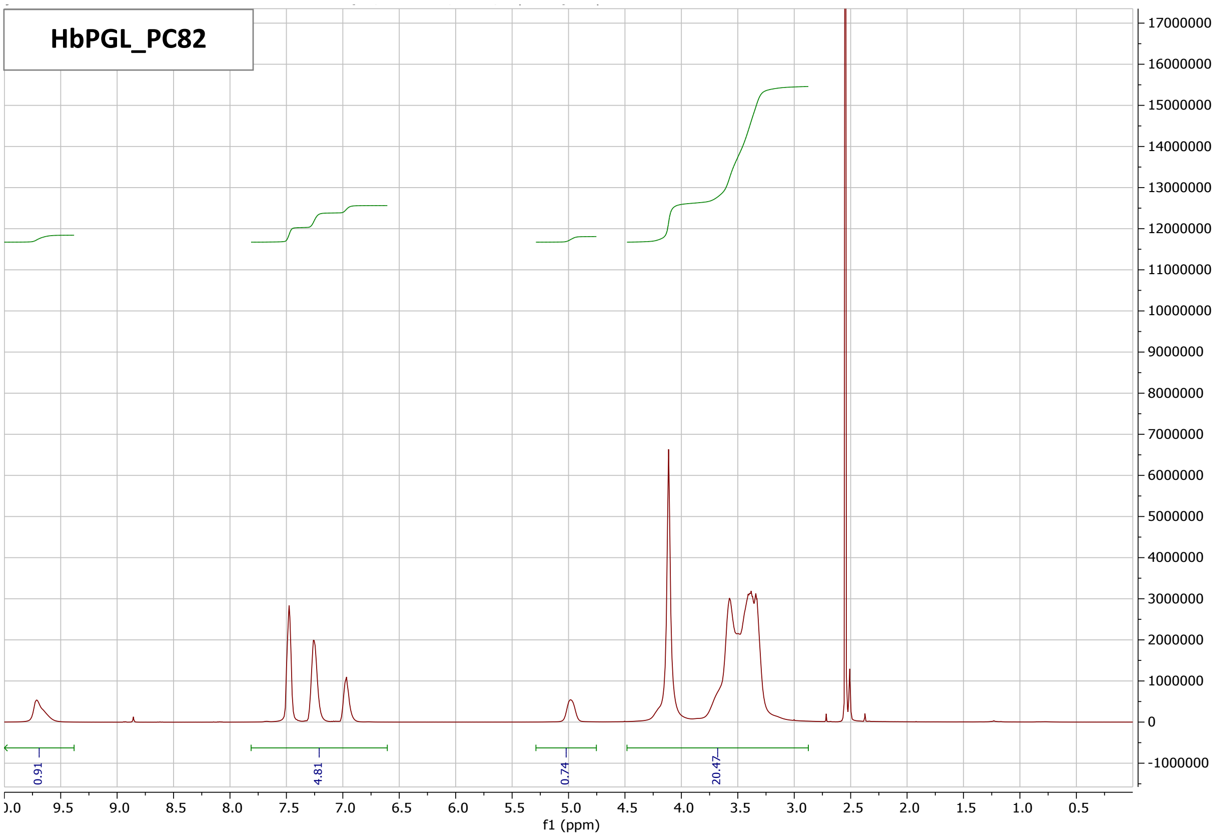

Figure S61. ^{1}H NMR spectrum of HbPGL_PC82 recorded in DMSO-d_6.

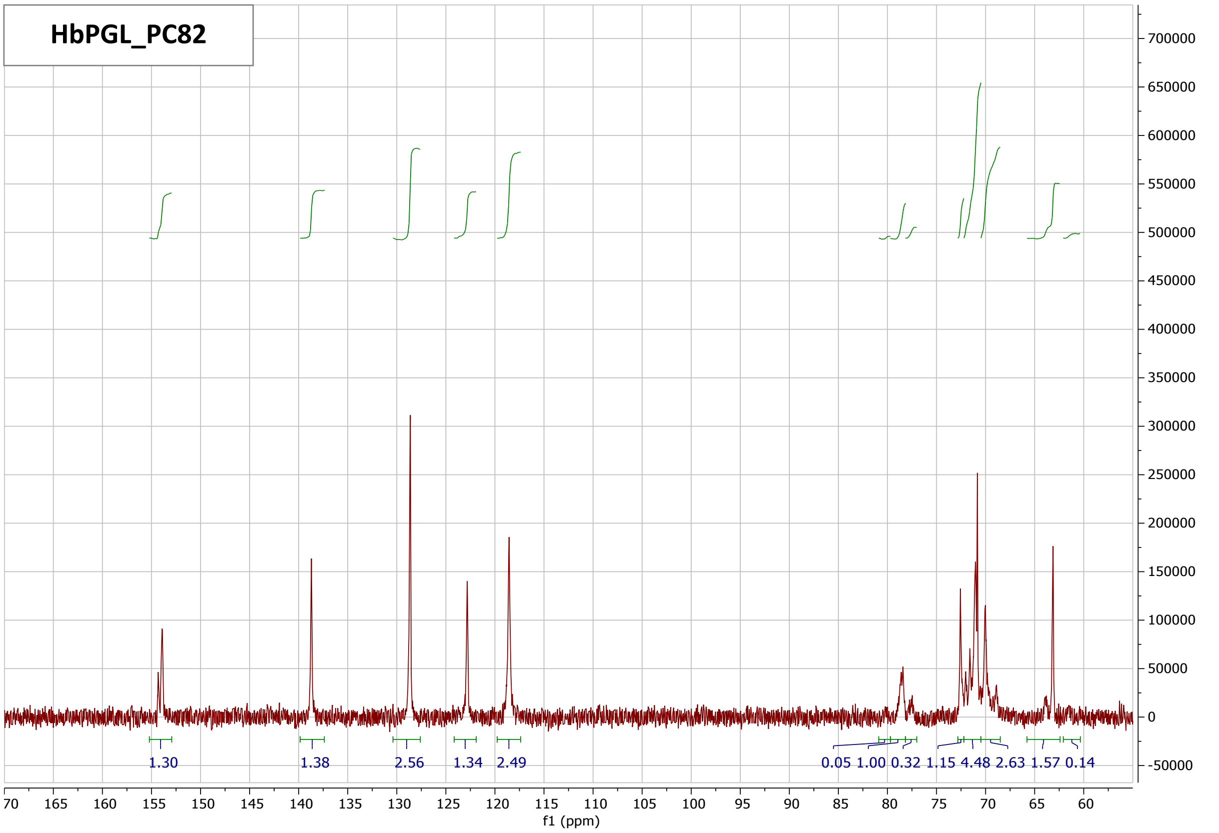

Figure S62. ^{13}C INVGATED NMR spectrum of HbPGL_PC82 recorded in DMSO-d_6.

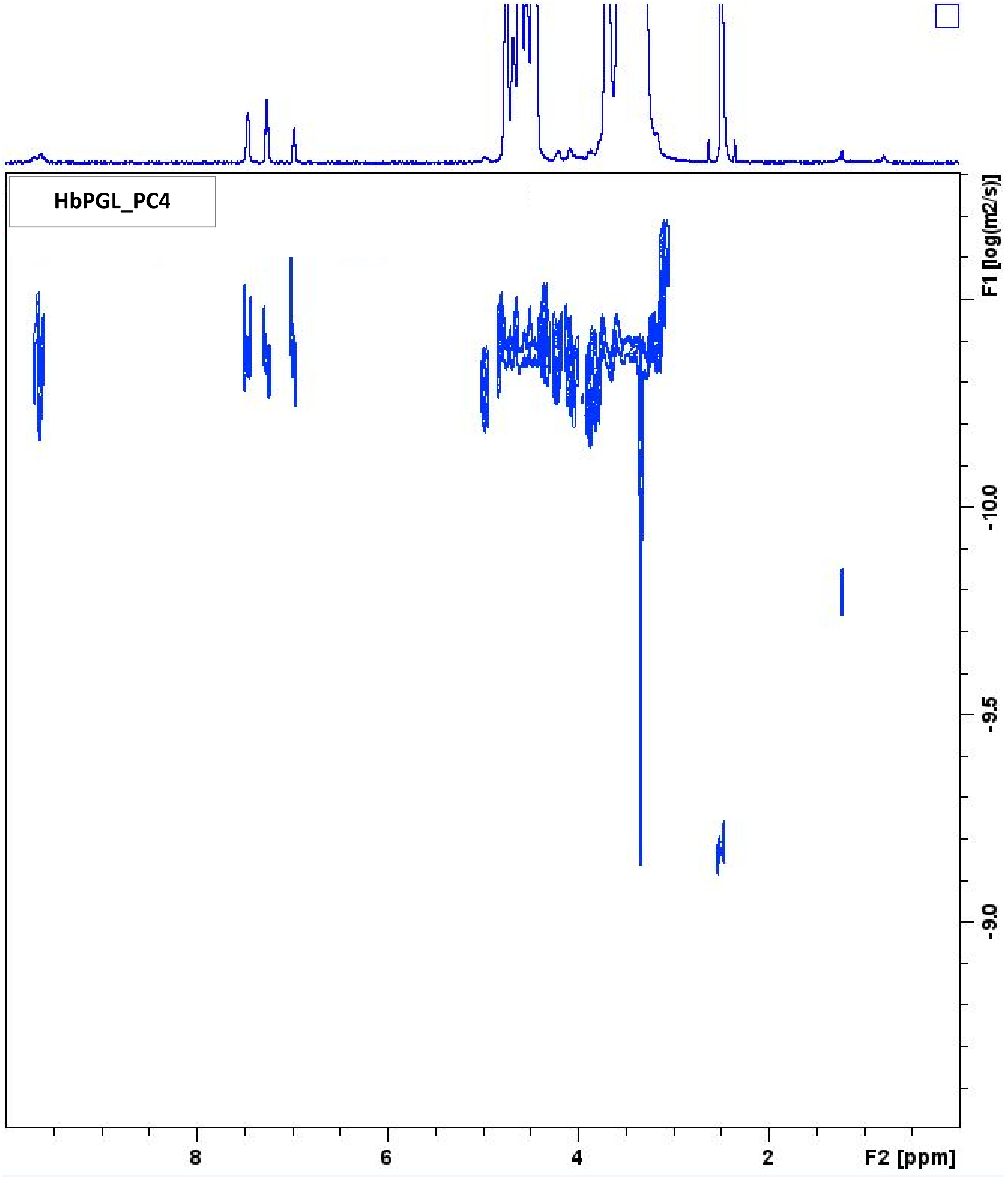

Figure S63. ^{1}H DOSY NMR spectrum of HbPGL_PC4 recorded in DMSO-d_6.

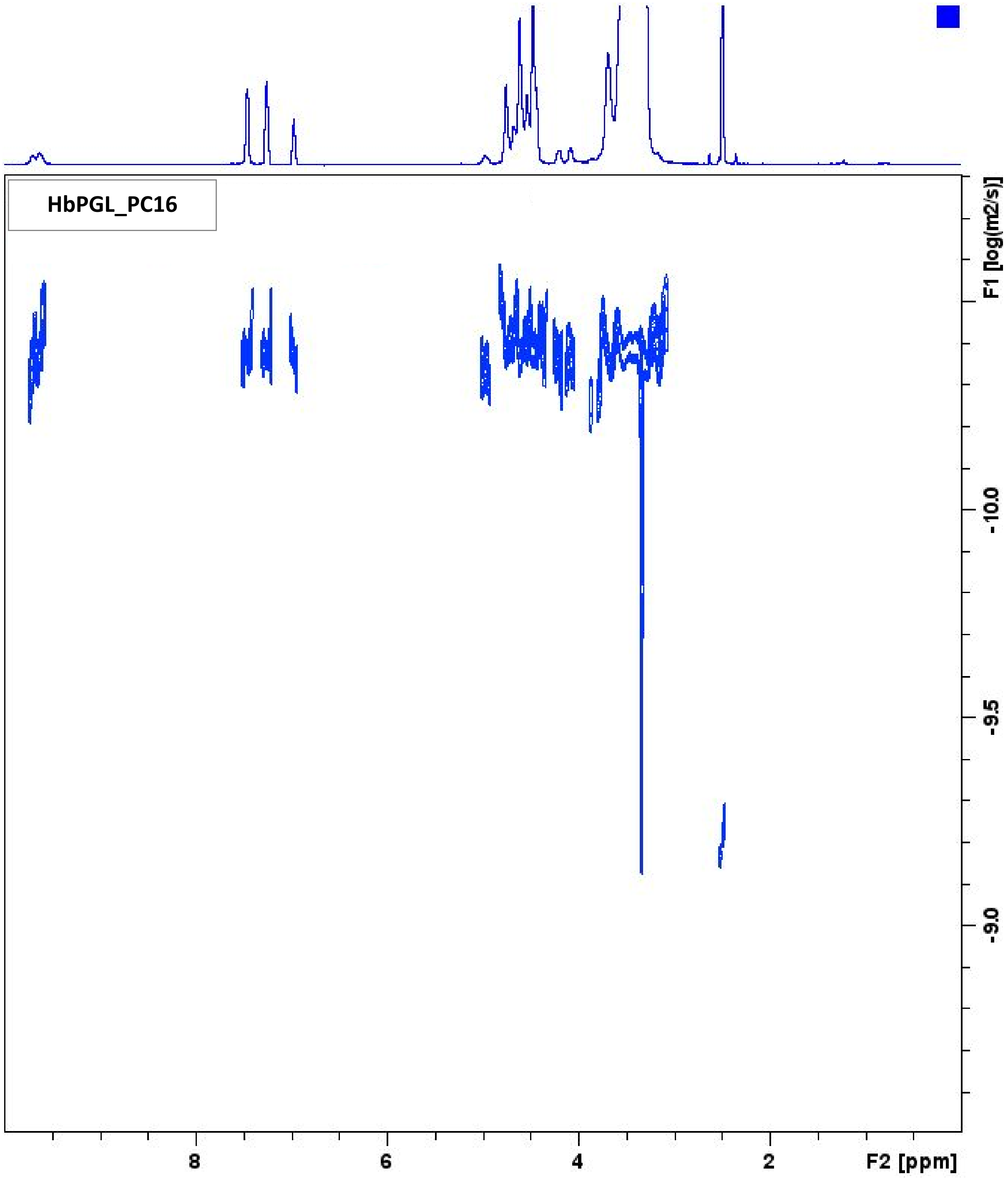

Figure S64. ^{1}H DOSY NMR spectrum of HbPGL_PC16 recorded in DMSO-d_6.

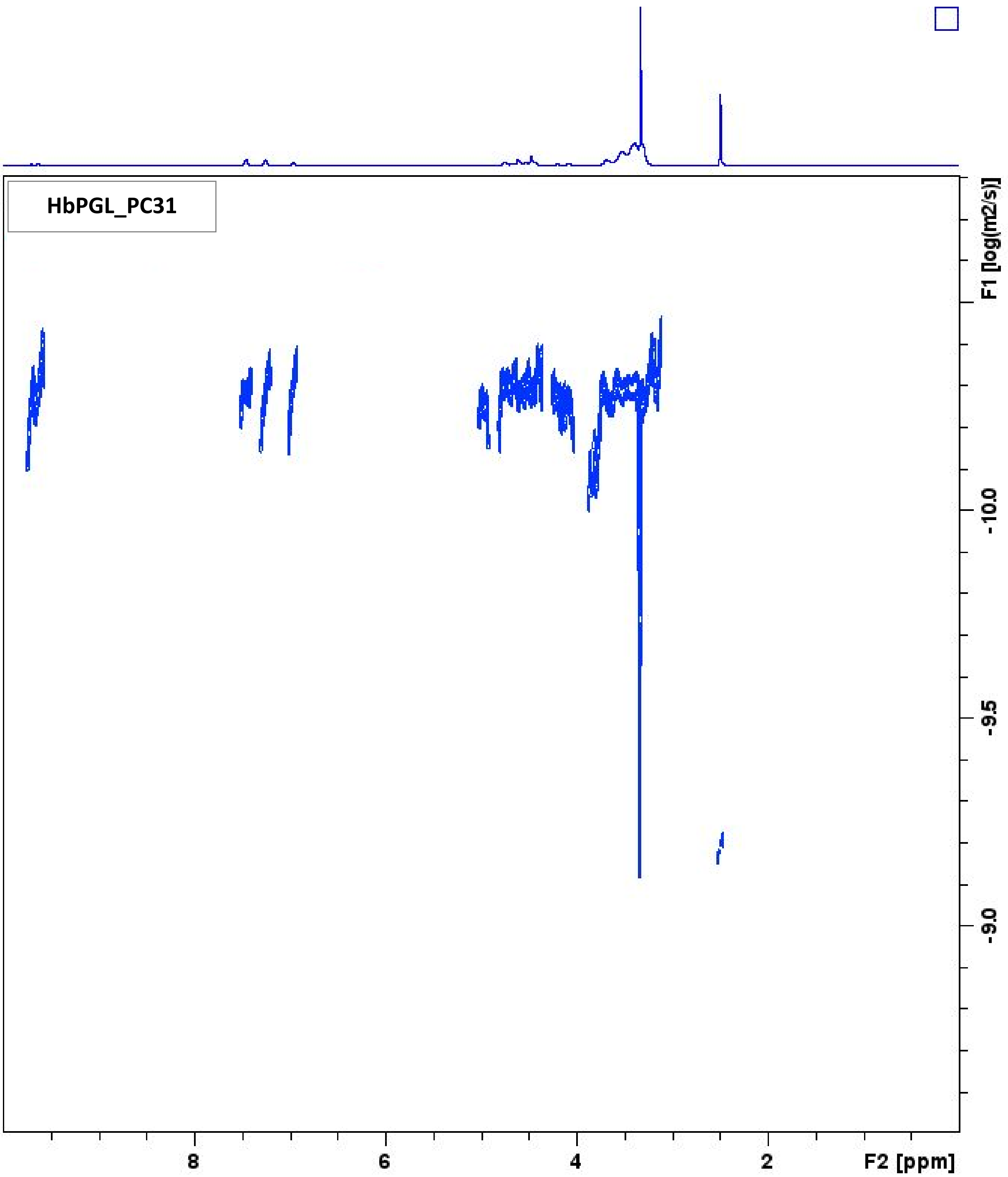

Figure S65. ^{1}H DOSY NMR spectrum of HbPGL_PC31 recorded in DMSO-d_6.

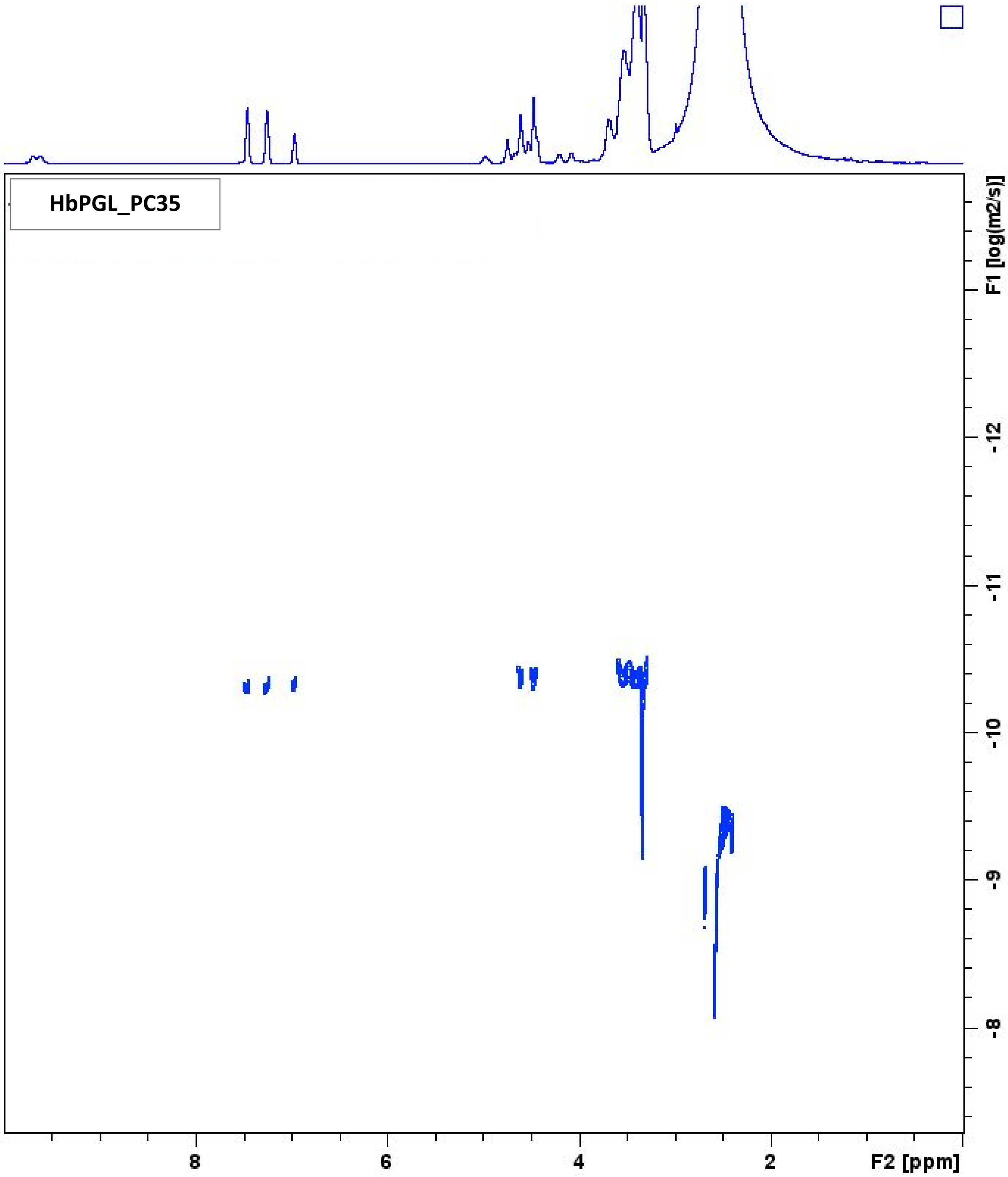

Figure S66. ^{1}H DOSY NMR spectrum of HbPGL_PC35 recorded in DMSO-d_6.

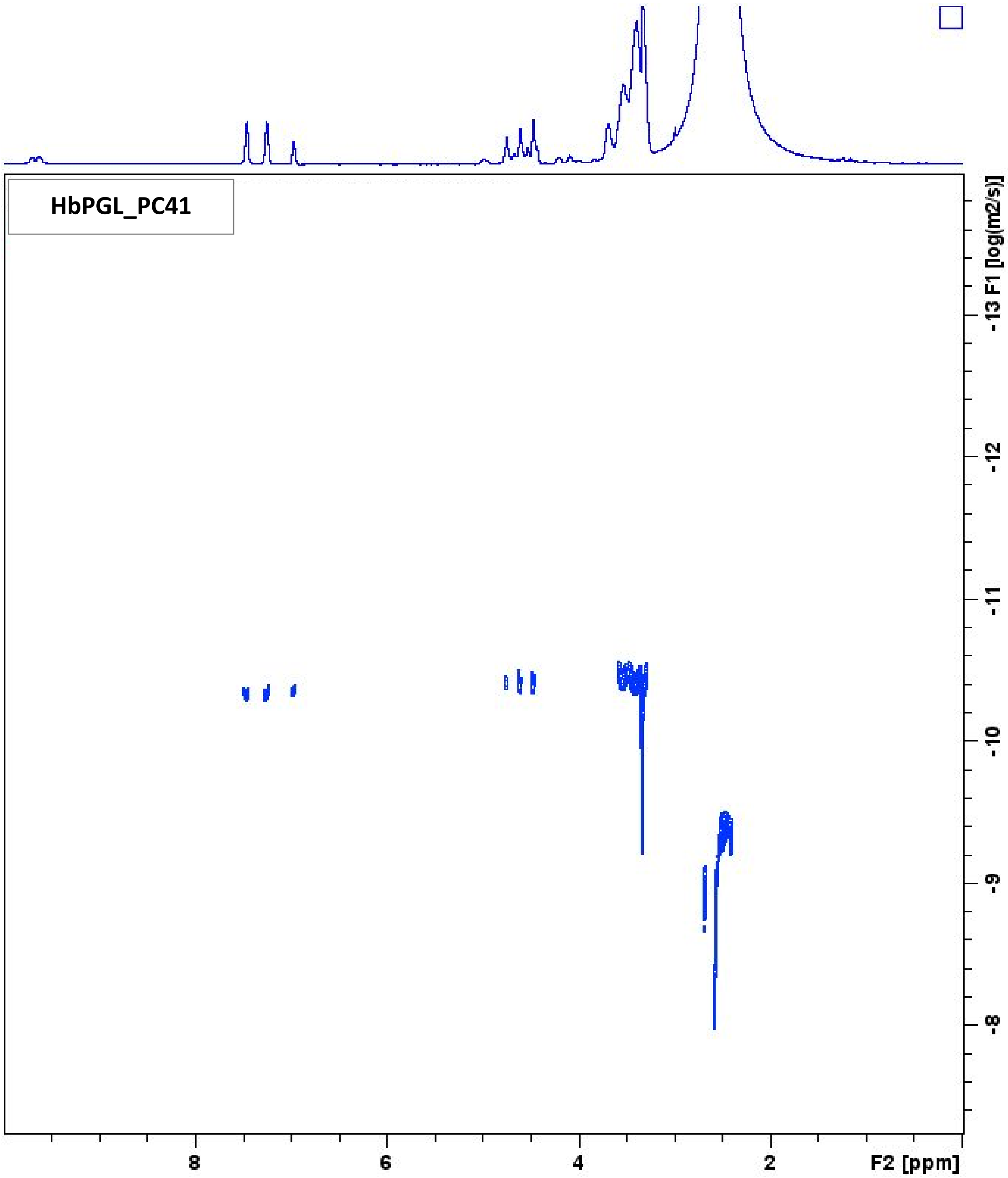

Figure S67. ^{1}H DOSY NMR spectrum of HbPGL_PC41 recorded in DMSO-d_6.

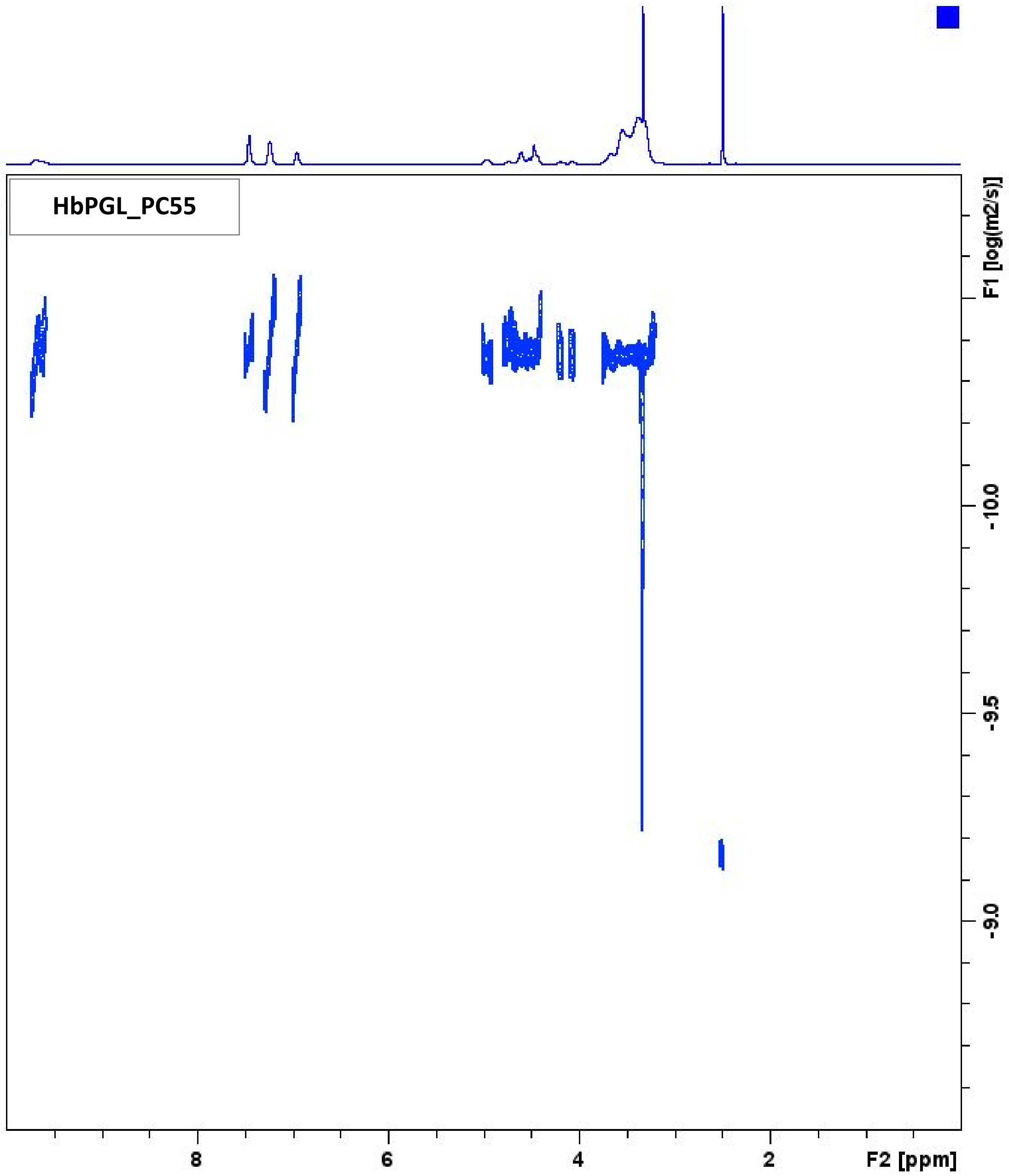

Figure S68. ^{1}H DOSY NMR spectrum of HbPGL_PC55 recorded in DMSO-d_6.

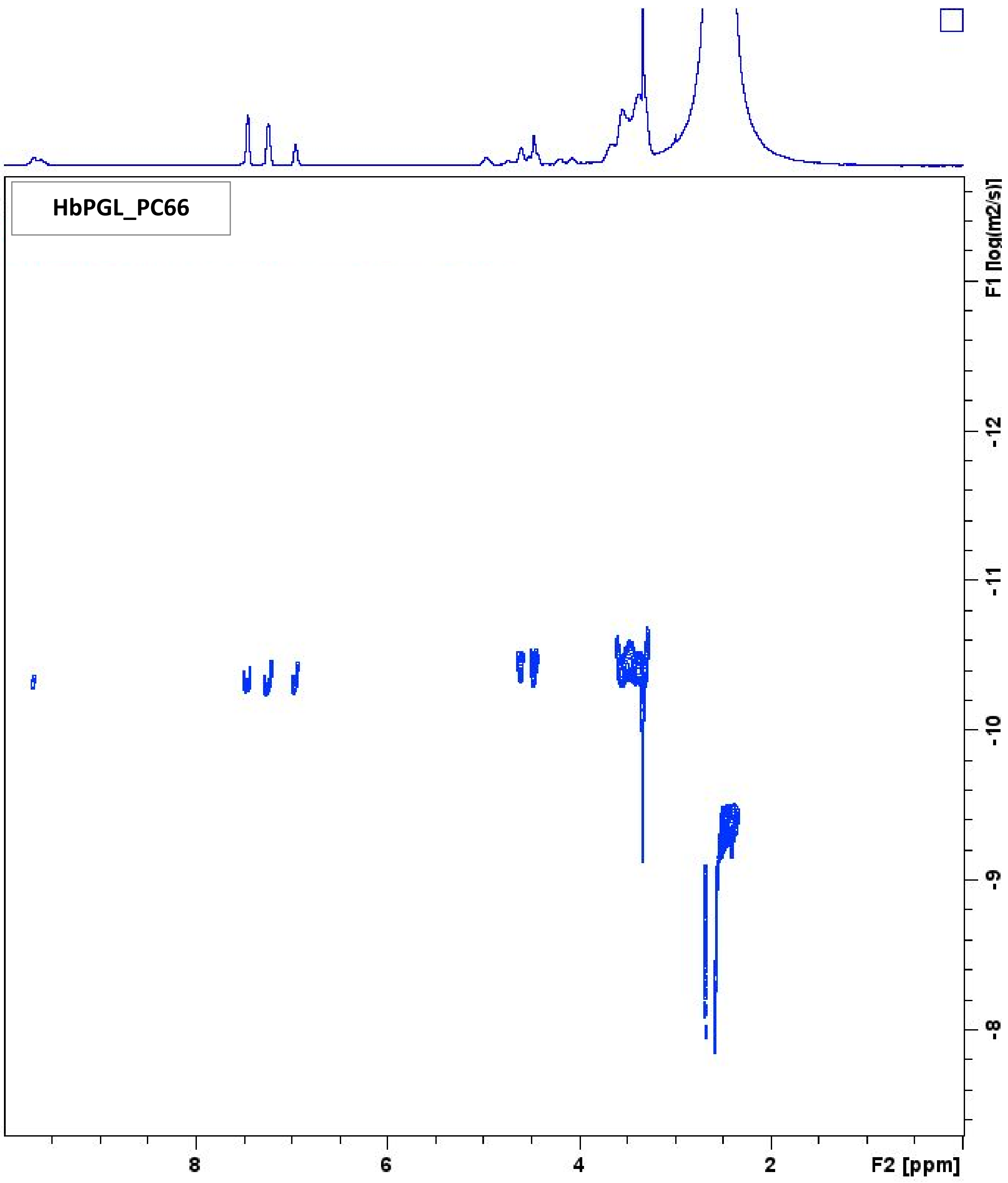

Figure S69. ^{1}H DOSY NMR spectrum of HbPGL_PC66 recorded in DMSO-d_6.

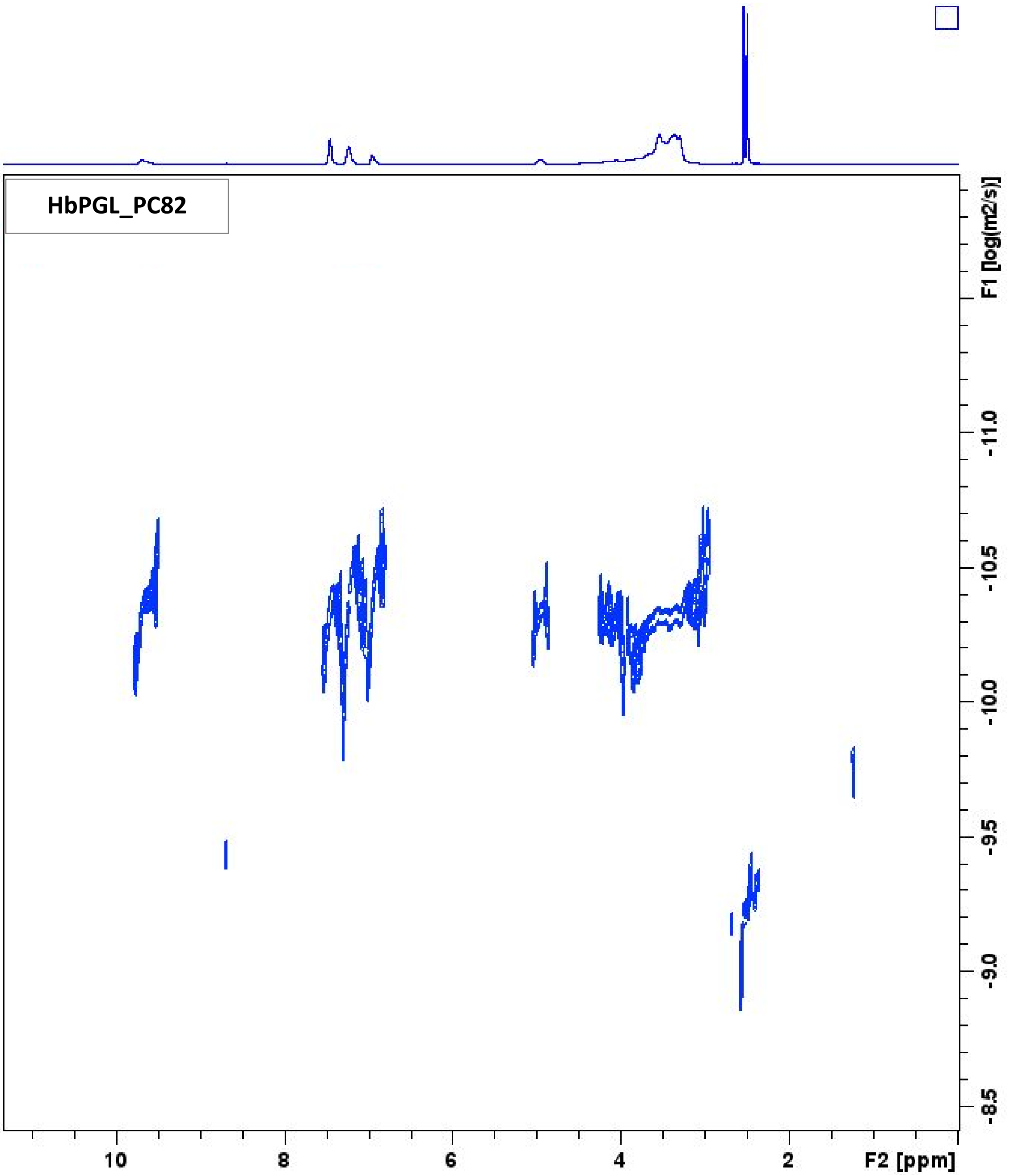

Figure S70. ^{1}H DOSY NMR spectrum of HbPGL_PC82 recorded in DMSO-d_6.

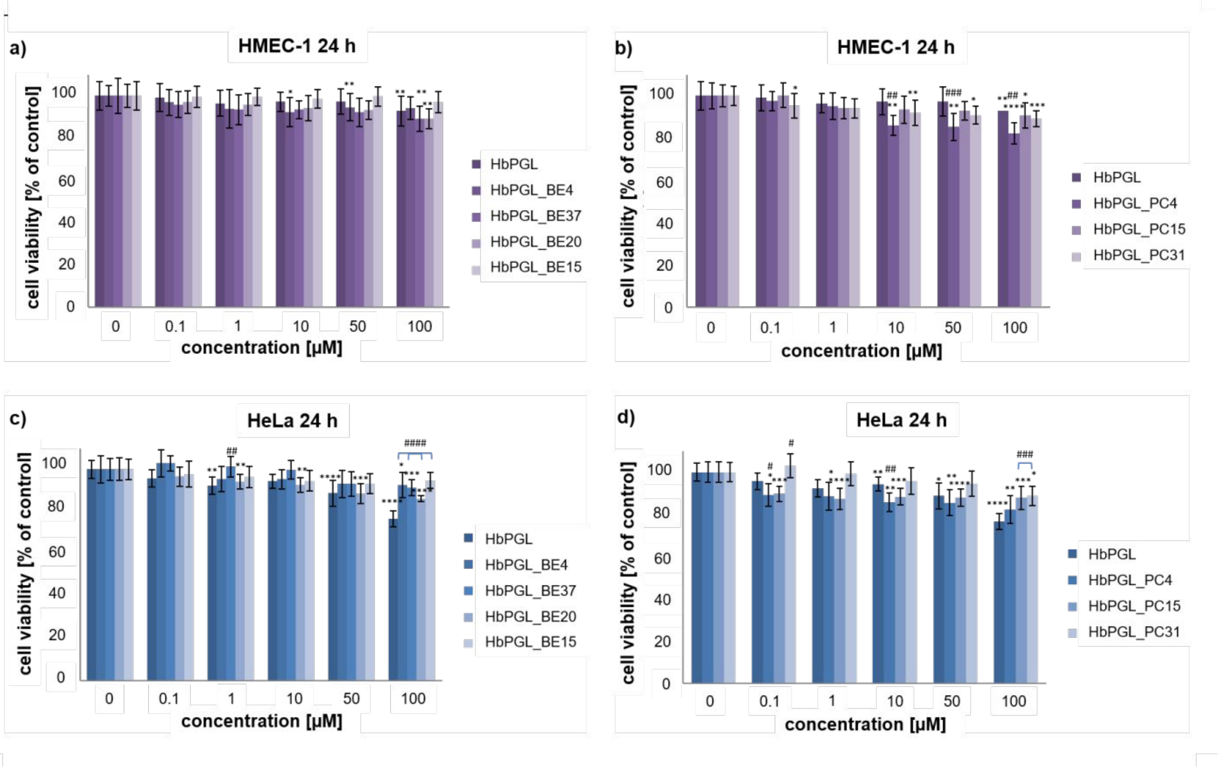

Figure S71. The influence of molar concentration of the neat hyperbranched polyglycidol and its hydrophobized derivatives on the cell viability of HMEC-1 (a, b) and HeLa (c, d), respectively after 24 incubation at 37 ^{o}C. Data are presented as a percentage of control (untreated cells) standard deviation (SD). The cytotoxicity values compared to the untreated control (* p< 0.05, **p < 0.01, ***p < 0.005, ****p < 0.0001) as well as between unmodified hyperbranched polyglycidol and modified ones at the same compound concentration (# p< 0.05, ## p < 0.01, ### p < 0.005, ####p < 0.0001).

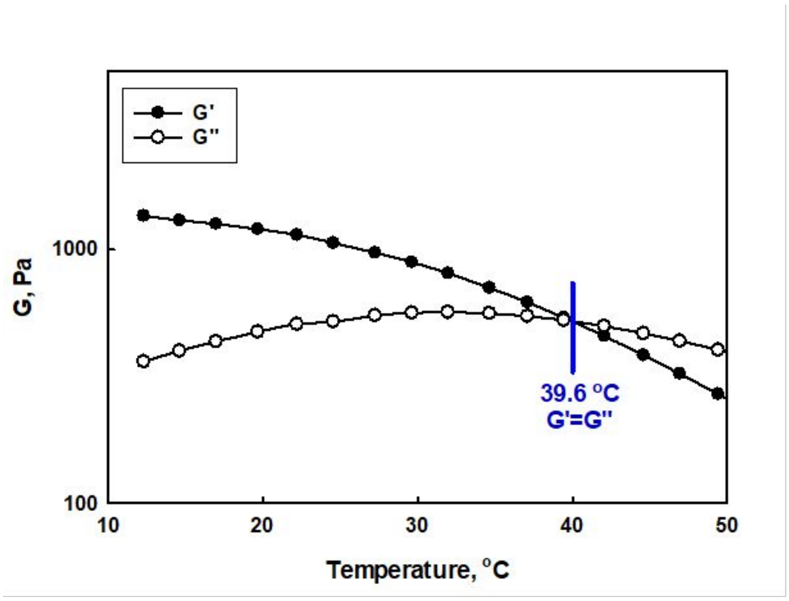

Figure S72. Temperature sweep test performed for hydrogel composed of neat HbPGL cross-linked with poly(AM-*ran*-2-AAPBA).

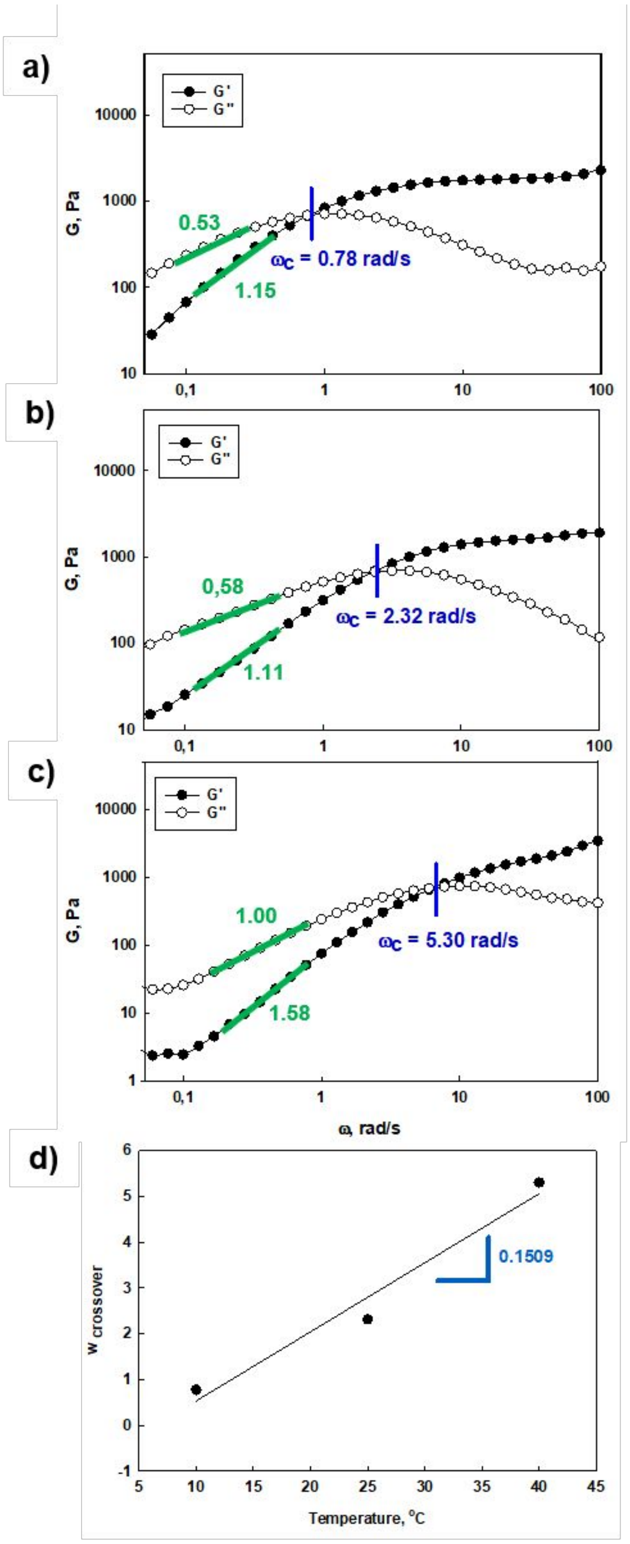

Figure S73. Frequency sweep tests performed for a hydrogel constructed of neat HbPGL cross-linked with poly(AM-ran-2-AAPBA) at 10 °C (a), 25 °C (b) and 40 °C (c) and plotted the temperature dependence of ω_c. (d).

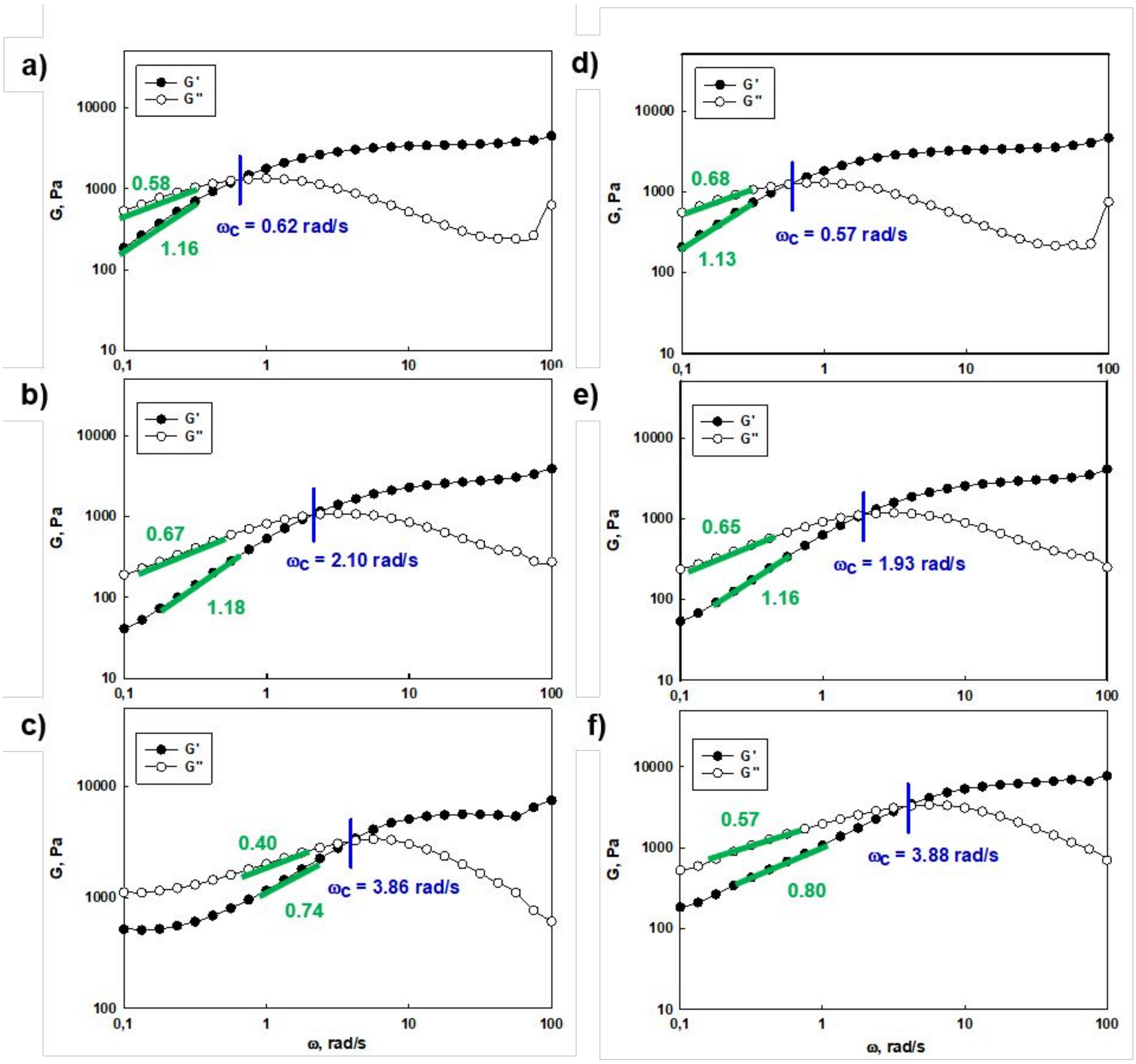

Figure S74. Frequency sweep tests performed for hydrogels constructed of poly(AM-ran-2-AAPBA) cross-linked respectively with HbPGL_BE4 recorded at 10 ^{o}C (a), 25 ^{o}C (b) and 40 ^{o}C (c) and with HbPGL_PC4 recorded at 10 ^{o}C (d), 25 ^{o}C (e) and 40 ^{o}C (f).

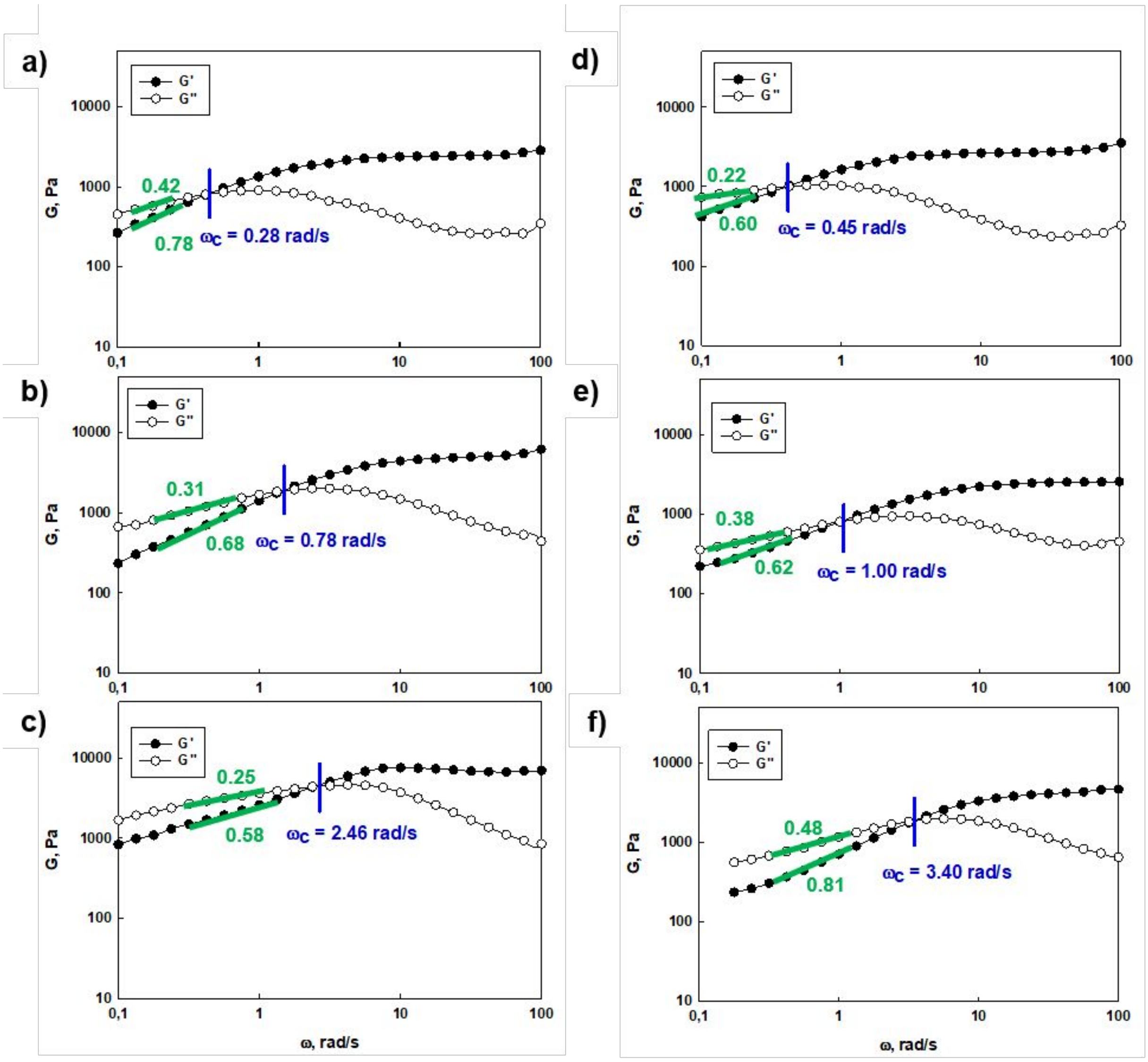

Figure S75. Frequency sweep tests performed for hydrogels constructed of poly(AM-ran-2-AAPBA) cross-linked respectively with HbPGL_BE15 recorded at 10 °C (a), 25 °C (b) and 40 °C (c) and with HbPGL_PC15 recorded at 10 °C (d), 25 °C (e) and 40 °C (f).

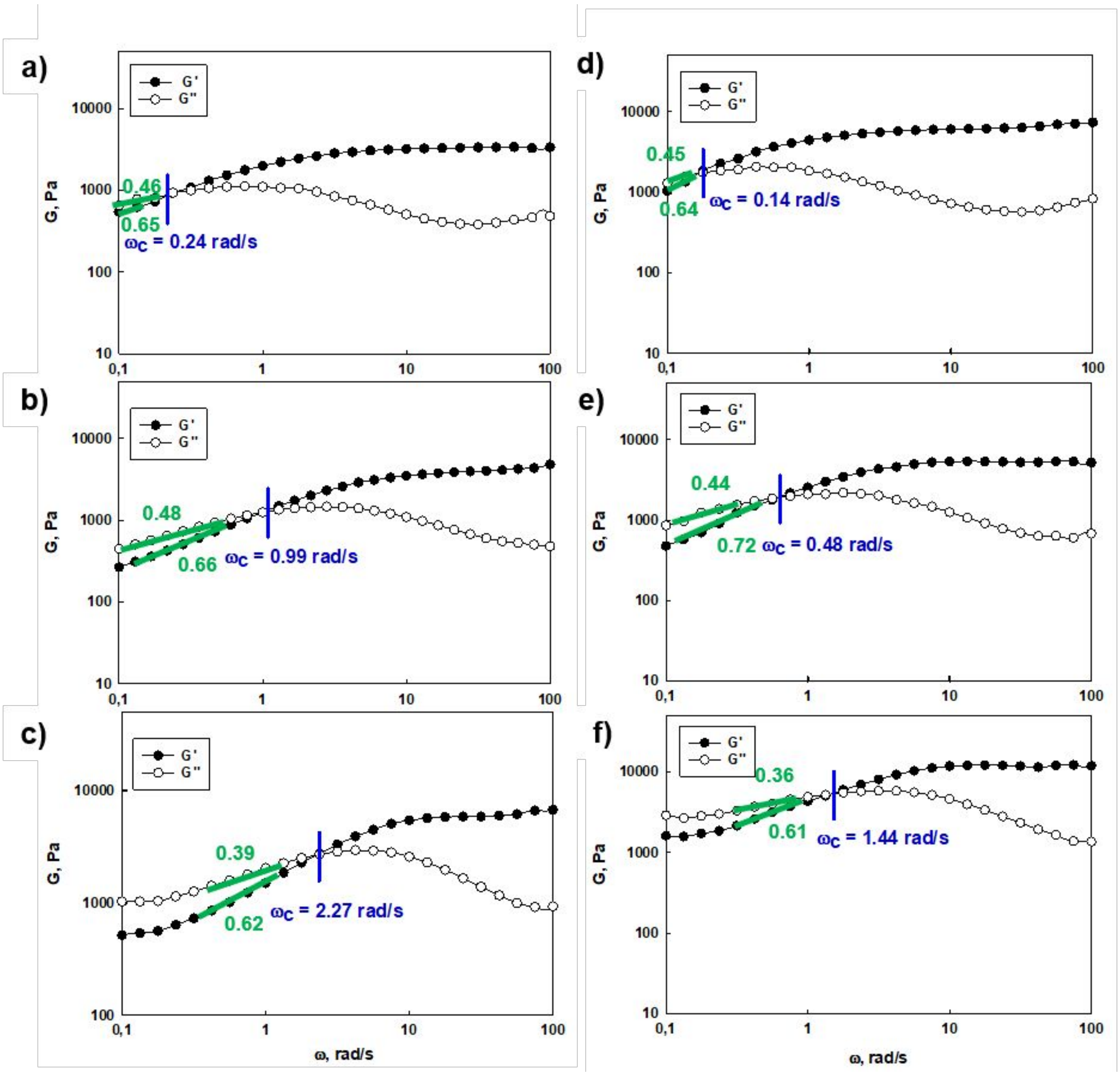

Figure S76. Frequency sweep tests performed for hydrogels constructed of poly(AM-ran-2-AAPBA) cross-linked respectively with HbPGL_BE26 recorded at 10 ^{o}C (a), 25 ^{o}C (b) and 40 ^{o}C (c) and with HbPGL_PC31 recorded at 10 ^{o}C (d), 25 ^{o}C (e) and 40 ^{o}C (f).

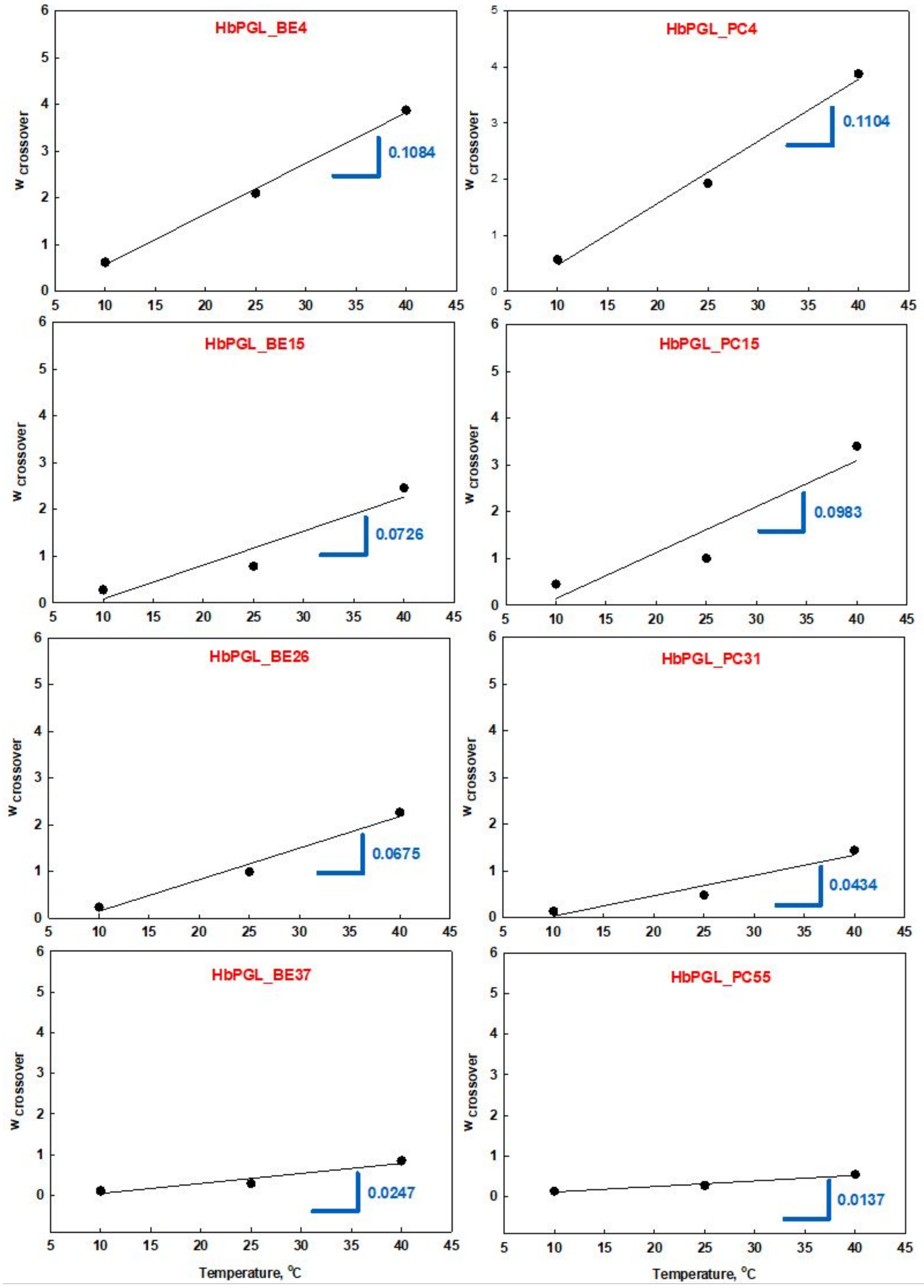

Figure S77. The temperature dependence of $\omega_{crossover}$ recorded for hydrogels constructed of hydrophobized HbPGL at different degree cross-linked with poly(AM-ran-2-AAPBA).

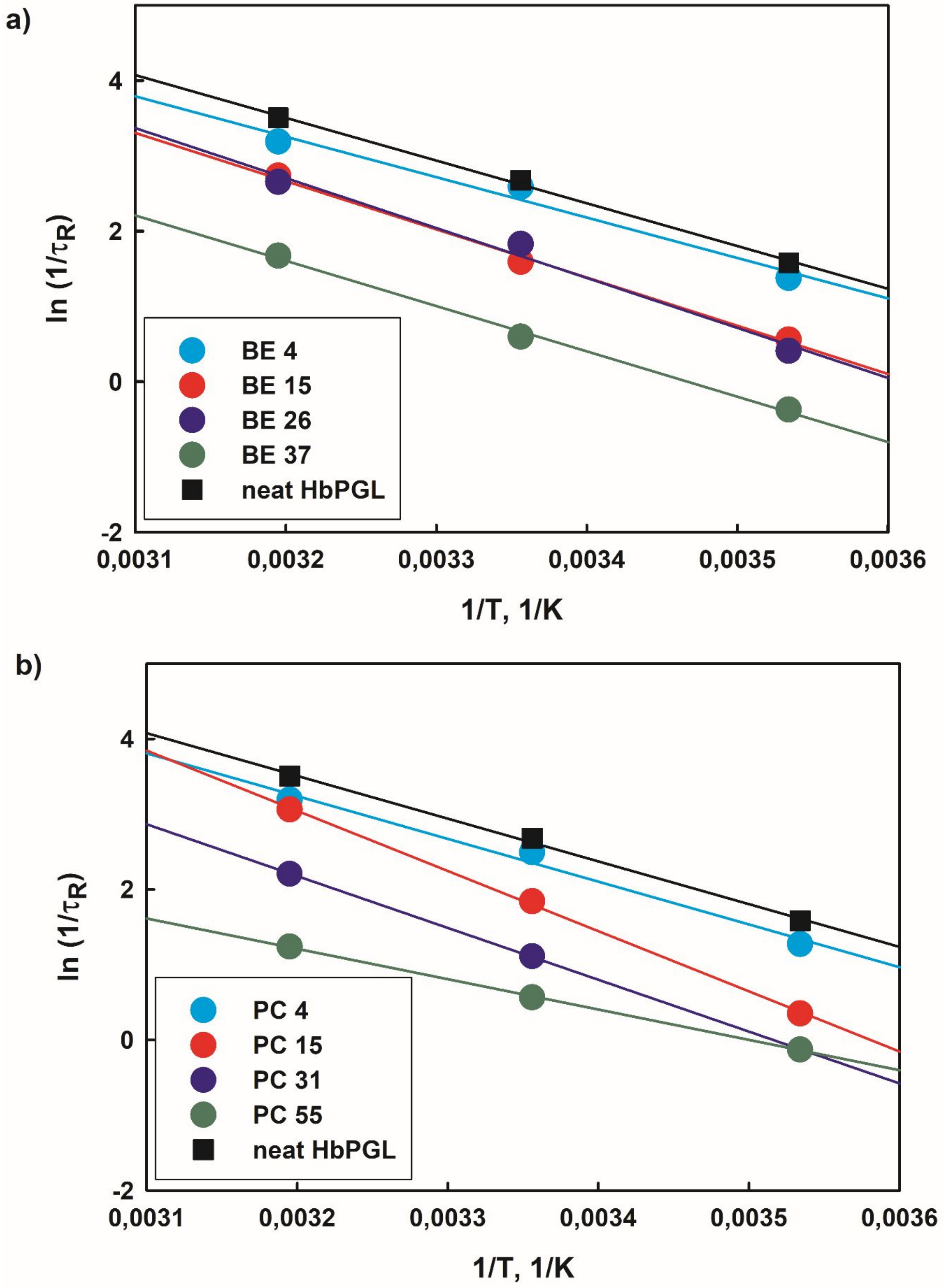

Figure S78. The dependence of $\ln(1/\tau_R)$ on 1/T determined for hydrogels constructed of HbPGL hydrophobized with phenyl units incorporated via ester (a) or urethane (b) linkages.

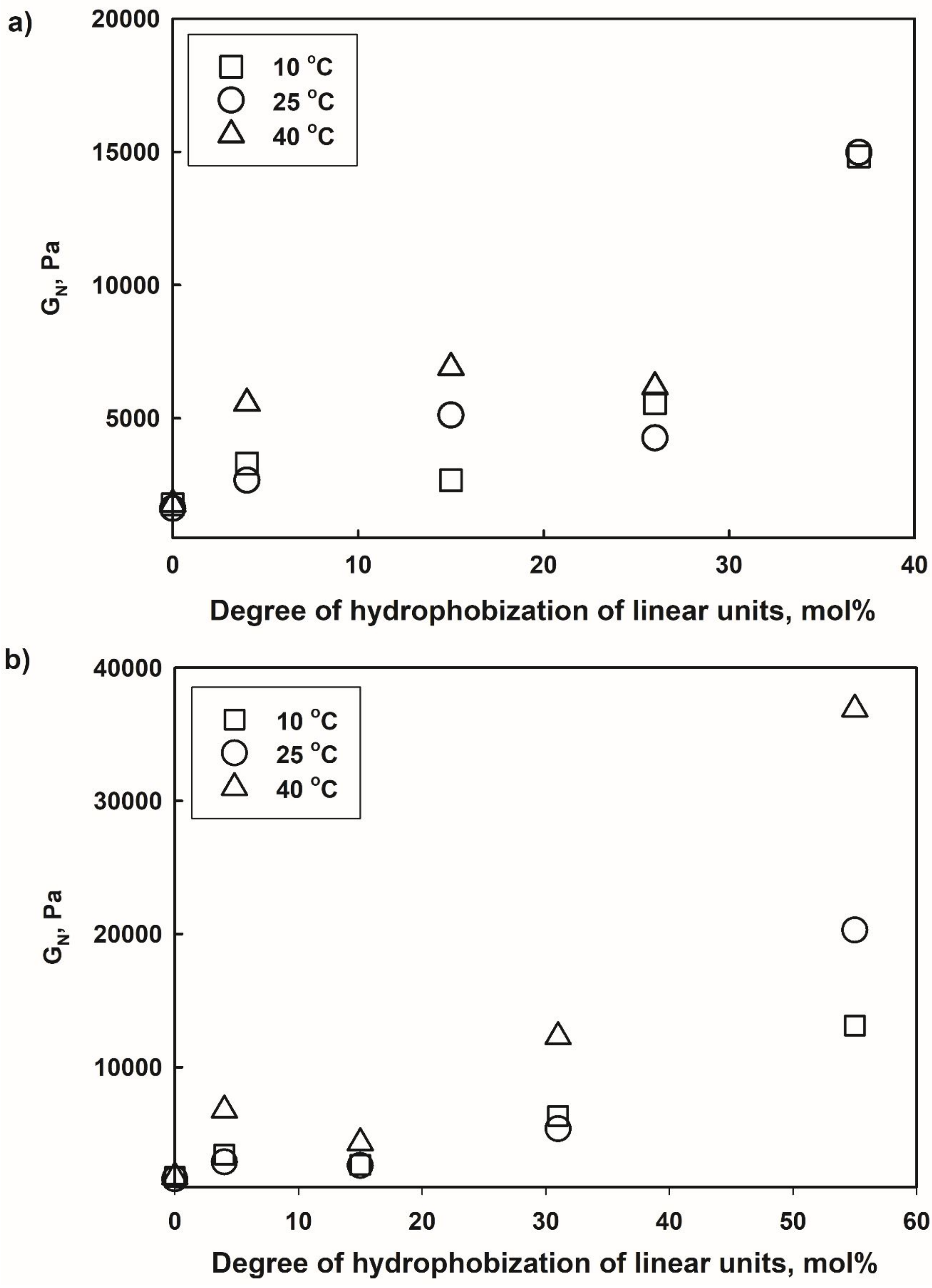

Figure S79. The dependence of G_N of hydrogels on the hydrophobization degree of used HbPGL performed via incorporation of phenyl groups via ester (a) and urethane (b) linkages.

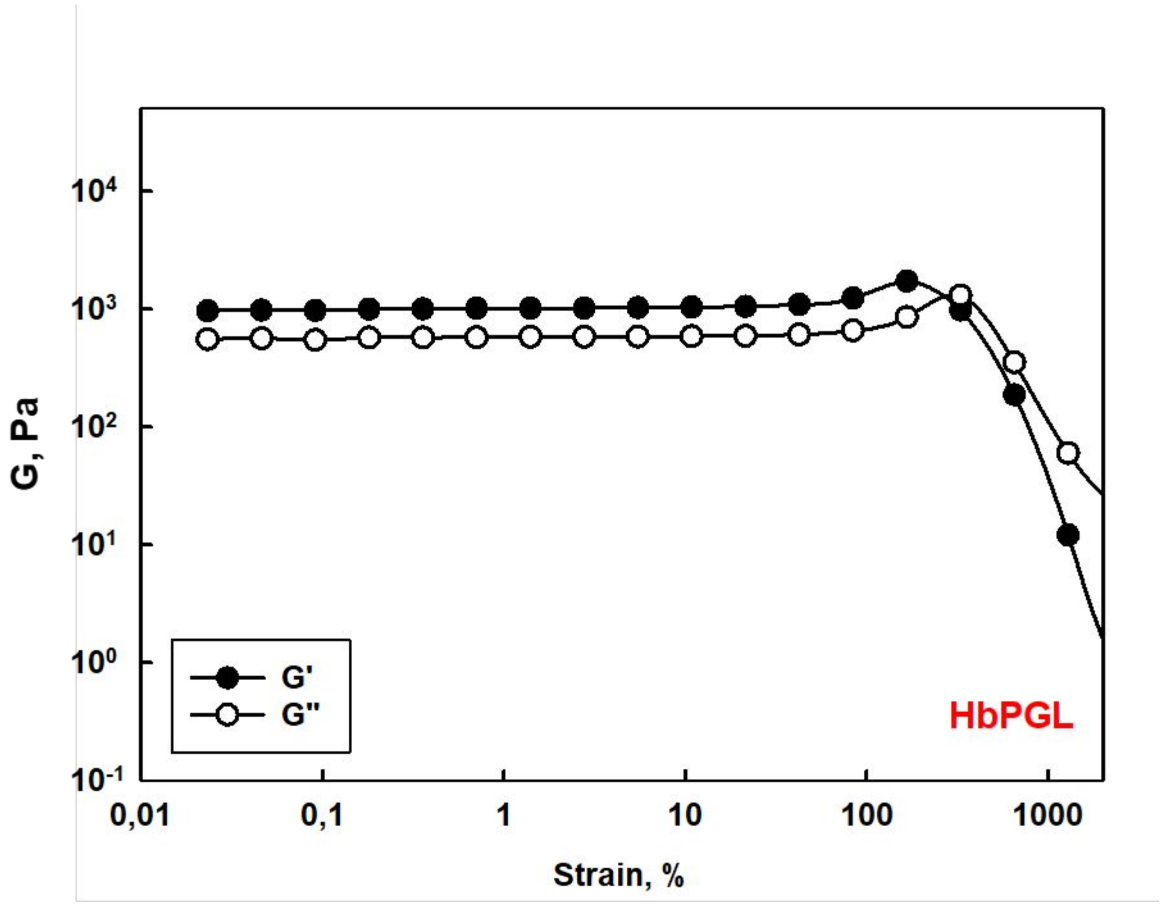

Figure S80. Strain sweep experiments performed for the hydrogel constructed of neat HbPGL cross-linked with poly(AM-*ran*-2-AAPBA).

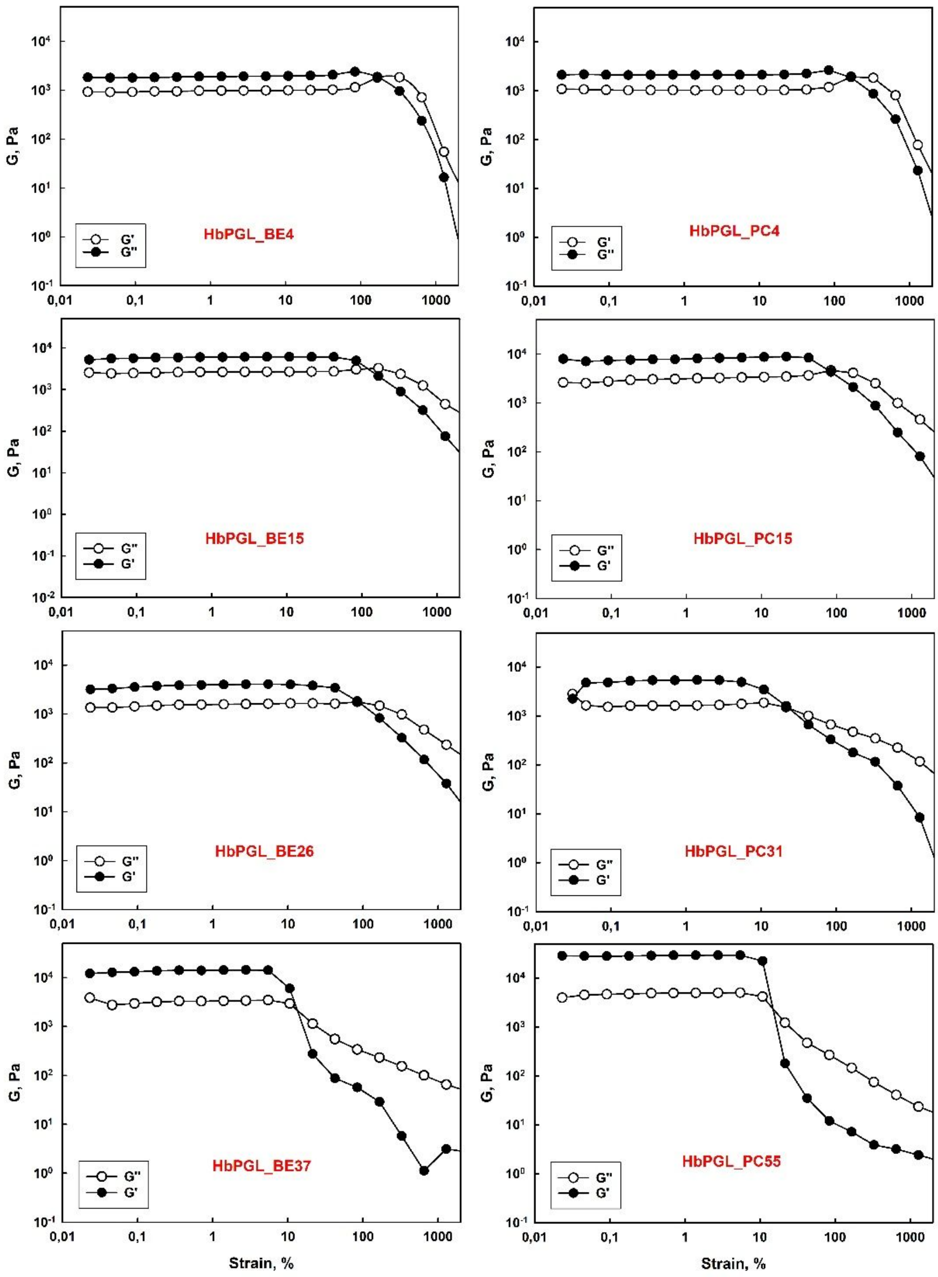

Figure S81. Strain sweep experiments performed for hydrogels constructed of hydrophobized HbPGL cross-linked with poly(AM-*ran*-2-AAPBA).

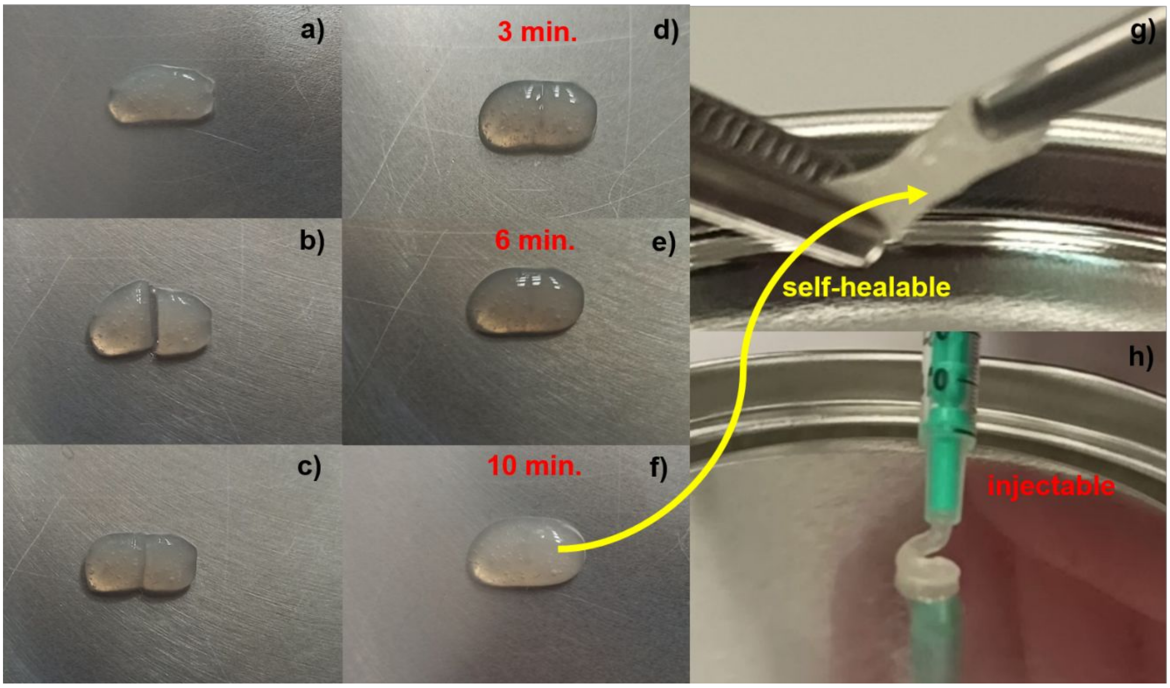

Figure S82. The illustration of experiment demonstrating the self-healable behaviour of neat HbPGL-based hydrogel. The hydrogel sample (a) was ruptured into two pieces (b), and placed in close contact (c). The sample was gradually repaired after 3 min. (d), 5 min. (e), 10 min. (f), respectively, and then the sample was raised with a couple of tweezers (g), which revealed that the sample was self-healable under ambient conditions. The hydrogel was injected via a syringe (h). The hydrogel after extrusion from the syringe preserves its integrity. In addition, a video file presenting an experiment of the hydrogel injection via a needle was attached.

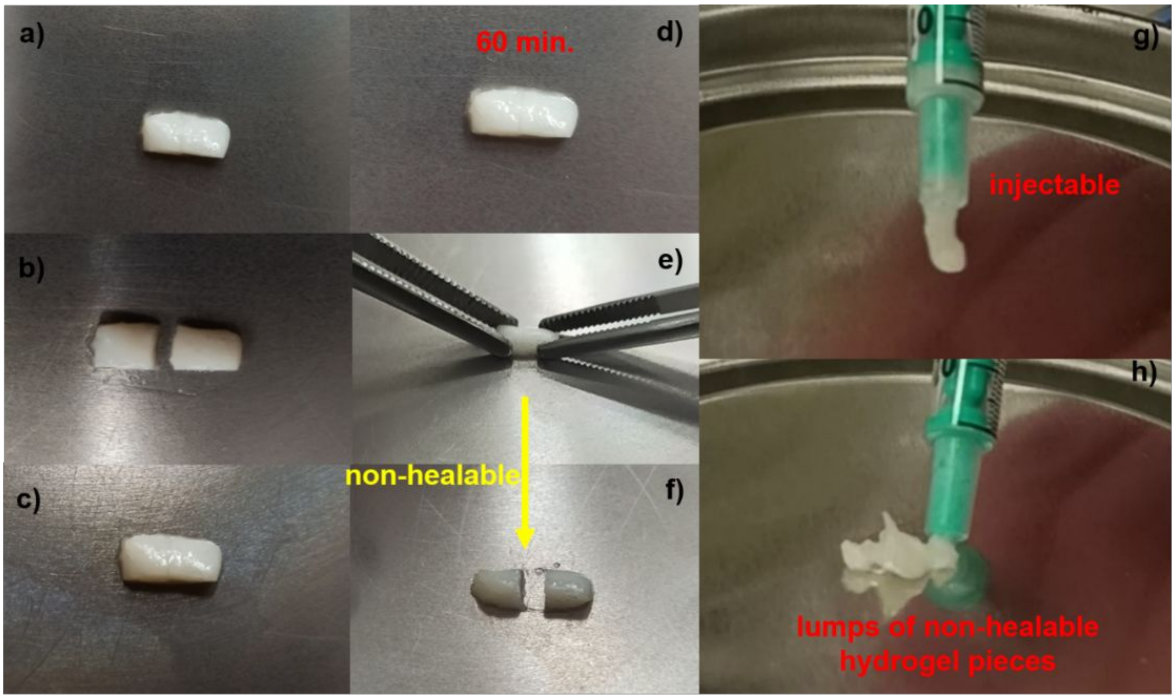

Figure S83. The illustration of experiment demonstrating the non-healable behaviour HbPGL_PC55-based hydrogel. The hydrogel sample (a) was ruptured into two pieces (b), and

placed in close contact (c). After 60 min. the sample was raised with a couple of tweezers (e), which revealed that the sample was not repaired (f). The hydrogel displayed the injectable properties, however, the sample was extruded in the form of separated non-healable lumps (h). In addition, a video file presenting an experiment of the hydrogel injection via a needle was attached.
